# Feedback, Mass Conservation and Reaction Kinetics Impact the Robustness of Cellular Oscillations

**DOI:** 10.1371/journal.pcbi.1005298

**Published:** 2016-12-27

**Authors:** Katharina Baum, Antonio Z. Politi, Bente Kofahl, Ralf Steuer, Jana Wolf

**Affiliations:** 1 Max Delbrück Center for Molecular Medicine in the Helmholtz Association, Berlin, Germany; 2 Institute for Theoretical Biology, Humboldt University of Berlin, Berlin, Germany; University of Virginia, UNITED STATES

## Abstract

Oscillations occur in a wide variety of cellular processes, for example in calcium and p53 signaling responses, in metabolic pathways or within gene-regulatory networks, e.g. the circadian system. Since it is of central importance to understand the influence of perturbations on the dynamics of these systems a number of experimental and theoretical studies have examined their robustness. The period of circadian oscillations has been found to be very robust and to provide reliable timing. For intracellular calcium oscillations the period has been shown to be very sensitive and to allow for frequency-encoded signaling. We here apply a comprehensive computational approach to study the robustness of period and amplitude of oscillatory systems. We employ different prototype oscillator models and a large number of parameter sets obtained by random sampling. This framework is used to examine the effect of three design principles on the sensitivities towards perturbations of the kinetic parameters. We find that a prototype oscillator with negative feedback has lower period sensitivities than a prototype oscillator relying on positive feedback, but on average higher amplitude sensitivities. For both oscillator types, the use of Michaelis-Menten instead of mass action kinetics in all degradation and conversion reactions leads to an increase in period as well as amplitude sensitivities. We observe moderate changes in sensitivities if replacing mass conversion reactions by purely regulatory reactions. These insights are validated for a set of established models of various cellular rhythms. Overall, our work highlights the importance of reaction kinetics and feedback type for the variability of period and amplitude and therefore for the establishment of predictive models.

## Author Summary

Rhythmic behavior is omnipresent in biology and has many crucial functions. In cells the activation levels and abundances of signaling molecules such as NF-κB, p53, EGFR or calcium repeatedly increase and decrease in response to stimuli. Such a dynamic behavior can also be observed monitoring the concentrations of mRNAs and proteins in the circadian clock and the cell cycle. Period and amplitude which are the time span between peaks and the peak height, respectively, as well as their variabilities are important features of oscillations. The circadian period is very stable allowing for proper time keeping, whereas in calcium signaling the period is very variable encoding different stimulation strengths. Our goal is to examine the origin of differences in sensitivities of periods and amplitudes using a computational approach. We use prototype oscillators and demonstrate that they can be used to derive general principles that explain the degree of robustness in period and amplitude for a set of commonly used models of cellular oscillators. Our findings imply that the robustness of oscillating systems can be influenced by feedback type and kinetic properties to which special attention should be paid when designing mathematical models of cellular rhythms.

## Introduction

Various self-sustained autonomous oscillations are found at the cellular level. Prominent examples are calcium, p53 and NF-κB oscillations in signaling systems, circadian and cell cycle oscillations in genetic networks and oxidation-reduction cycles in metabolism [1,2,3,4]. A central question is in how far these systems are able to maintain their dynamical characteristics facing environmental changes, a feature that has been termed robustness [5,6]. Mathematical models have been proposed for many oscillatory processes and the examination of their robustness is considered to give valuable indications on the organization and functioning of the respective underlying biological processes. A number of studies have focused on the size and shape of the parameter space that allows for oscillatory dynamics [6,7,8,9,10]. Yet, also the period and amplitude of oscillations may be differently robust to changes in the environment. For example, circadian oscillations endue a time-keeping function. It has been shown that their period of approximately 24 hours is temperature compensated and does not change significantly with varying pH or nutritional conditions [11,12,13,14]. In contrast, the period of intracellular calcium oscillations varies from seconds to minutes and is highly responsive to changes in temperature and agonist concentrations [15,16]. The latter is a phenomenon referred to as frequency encoding of the stimulus [17,18]. Furthermore, a robust amplitude has been shown to be important for the proper function of the cell cycle [19]. In this system, an amplitude reduction has been reported to result in disordered cell cycle events.

Mathematical models have been intensively used to analyze the period and amplitude sensitivities with respect to parameter perturbations. There have been mainly three computational approaches: (i) the viable region approach which examines the size of the parameter region of a certain period or amplitude [20,21]; (ii) the determination of the tunability of period or amplitude which captures the extent of their changes upon altering a parameter over a large range [22,23,24]; and (iii) sensitivity analyses which assess how strongly the period or amplitude changes upon small parameter perturbations, e.g. [20,25,26,27,28,29].

So far, the main goals of computational investigations have been to compare different model designs for a particular biological process [20,27], or to determine which parameters or types of parameters are the most sensitive for an oscillatory model [25,27,28]. It is, however, of particular interest which structural properties of a model render the period and the amplitude robust or sensitive. Such a knowledge is important to understand evolutionary mechanisms in multitasking systems: If certain structural properties already favor low or high period or amplitude sensitivities, the values of the parameters could be adapted during evolution with respect to other criteria, e.g. the capability of fast entrainment or specific phase relationships. Likewise, if certain structural properties are known to preferentially result in specific period or amplitude sensitivities, this knowledge could be used in the design of synthetic oscillators with requested characteristics.

A systematic analysis can be fostered by the analysis of prototype oscillators. Generally, biological oscillators have been classified into negative feedback oscillators, substrate-depletion oscillators and inhibitor-activator oscillators [30]. The period and amplitude sensitivities of a number of prototype oscillators within these classes have been investigated using the viable region approach (i) [21], the tunability assessment with respect to one particular kinetic parameter (ii) [22,24], or sensitivity analyses (iii) [26,29]. These studies have been mostly focused on the influence of the feedback type on sensitivity properties. While the viable region approach and the tunability assessment have been performed for multiple parameter sets for each model, the sensitivity analyses have been so far restricted to single parameter sets.

Here, we combined Monte-Carlo random sampling in the parameter space and sensitivity analyses with the aim to systematically investigate period and amplitude robustness for a large number of parameter sets. We characterized robustness by sensitivity measures summarizing the effect of single parameter perturbations. First, we asked whether particular period and amplitude sensitivities are inherent properties of the model and how strongly they can vary if other parameter sets are considered. To this end, we first investigated the sensitivities of one representative model for circadian and calcium oscillations each. Subsequently, we utilized a set of prototype oscillator models to determine which of the three following structural properties have an impact on period and amplitude robustness: the type of feedback; the reaction kinetics, in particular the impact of saturating interaction functions versus mass action kinetics; and the mass conservation properties, that is the impact of interactions that are governed by mass exchange, such as in metabolic conversion reactions, versus information transfer processes, as e.g. occurring in transcription and translation.

As prototype oscillators we employed chain models of length four with either a negative or a positive feedback. They represent two of three prototype oscillator classes mentioned above [30]. The chain model with a negative feedback constitutes a negative feedback oscillator. It resembles the Goodwin model [31] which has been extensively used in a number of studies, e.g. in [32,33,34]. The chain model with a positive feedback represents a substrate-depletion oscillator. The structures of our two prototype oscillators are very similar with respect to the number of species and the position of the exerted feedback. This enables a direct comparison of the impact of the structural properties of the models on the sensitivities.

Our analyses demonstrate that not only the feedback type, but also the considered kinetics and mass conservation properties have an impact on the period and amplitude sensitivities obtained in oscillating models. We validated the applicability of our results concerning feedbacks and kinetics using a set of established models of circadian and calcium oscillations. Moreover, we confirmed the effects of the kinetics for additional oscillatory models.

## Results

### Model structure and parameter choice affect the sensitivities

Biological oscillators have been studied in great detail and described by mathematical models that capture the main characteristics of the underlying processes. Here, we are interested to which extent the response towards perturbations depends on the model properties. Therefore, we examined the robustness of mathematical models with a focus on sustained oscillations and the sensitivities of period and amplitude. Environmental changes are represented by perturbations of the kinetic parameters of the models. We performed sensitivity analyses using measures which enable the comparison of models with different topologies, number of parameters, periods, and amplitudes. The non-dimensional period and amplitude sensitivities (Eqs 1 and 2, respectively) are given by

$$\sigma_{T}=\sqrt{\frac{1}{r}\sum_{l=1}^{r}{\left(R_{l}^{T}\right)}^{2}}$$ (Eq. 1)

and

$$\sigma_{A}=\sqrt{\frac{1}{r}\sum_{l=1}^{r}{\left(R_{l}^{A}\right)}^{2}},$$ (Eq. 2)

with *r* being the number of perturbed parameters [35,36]. In the equations above, the sensitivity coefficients

$$R_{l}^{T}=\frac{\Delta T/T}{\Delta {par}_{l}/{par}_{l}}$$ (Eq. 3)

and

$$R_{l}^{A}=\frac{\Delta A/A}{\Delta {par}_{l}/{par}_{l}}$$ (Eq. 4)

quantify relative changes in period *T* and amplitude *A* upon changes in a parameter *par*_*l*_ (see Methods). Here, we use the mean of the amplitude of all model variables as amplitude *A*. Thus, the sensitivities *σ*_*T*_ and *σ*_*A*_ measure how strongly period and amplitude of the oscillations are affected by parameter perturbations.

The question arises to which extent the calculated sensitivities vary for different parameter sets of the model. To address this question we employed a random sampling approach to obtain multiple parameter sets and combined it with sensitivity analyses. Specifically, we applied a bottom-up sampling in which the steady state concentrations and reaction flows are sampled directly over seven orders of magnitude. The rate coefficients are then calculated from the sampled values. Details are provided in the Methods section. For each model, parameter sets are sampled until the period and amplitude sensitivities for 2 500 different parameter sets yielding sustained oscillations for each parameter perturbation could be analyzed. Depending on the specific model between 5.9·10^4^ and 2.4·10^7^ parameter sets had to be sampled (numbers given in the S1 File).

The resulting data are depicted in scatter plots where each dot represents the period sensitivity and the amplitude sensitivity obtained for one particular parameter set. The median values of the distributions indicate the value around which the sensitivities are centered. They give an estimate of the sensitivities that can be expected for the model in general. The widths of the distributions (as measured by the 90% data range) show how the choice of a parameter set can alter the observed sensitivities and are considered as measures of variabilities. The scatter plots are accompanied by box-plots which capture important characteristics of the sensitivity distributions (see Methods).

**Fig 1:**
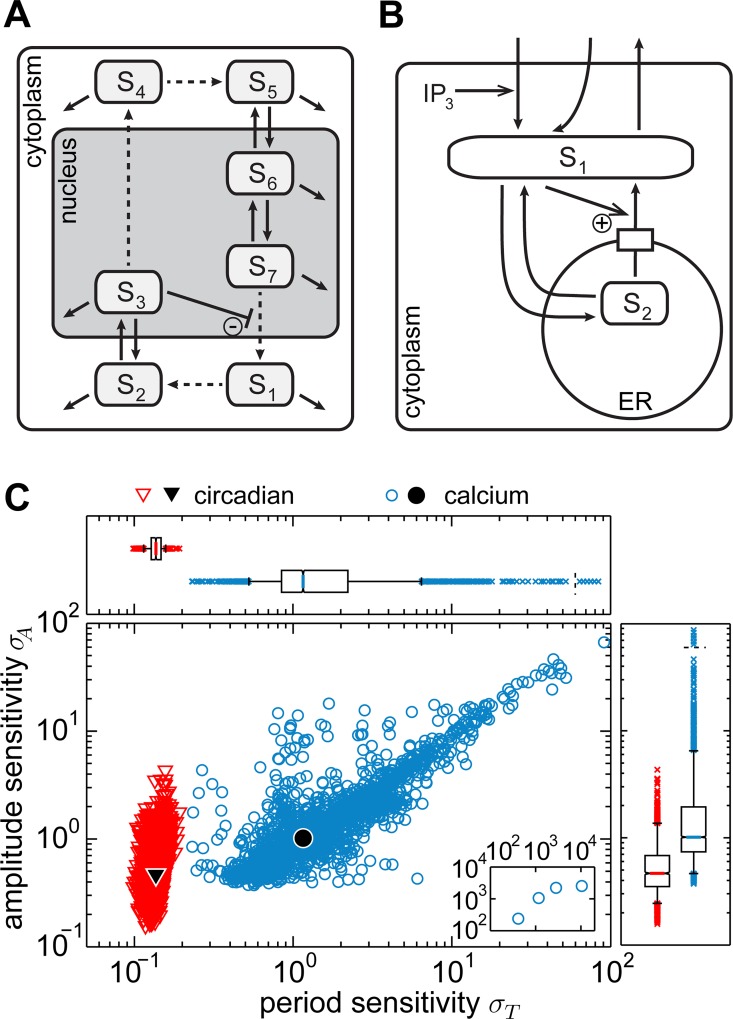
Sensitivity analysis of a circadian and a calcium oscillation model. A: Model scheme of the mammalian circadian oscillation model [37]. The basic negative feedback is marked by an encircled minus sign, purely regulatory interactions without mass flow are indicated by dashed arrows. B: Model scheme of a phenomenological calcium oscillation model [38]. The basic positive feedback is indicated by an encircled plus sign. C: Period and amplitude sensitivities for the circadian model (red triangles) and the calcium model (blue circles). Each point gives the sensitivities obtained for one parameter set. Median values are indicated by black symbols.

With this analysis work-flow, we first studied the sensitivities of a representative model of a circadian oscillator and of a representative model of a calcium oscillator. We chose the mammalian circadian rhythm model proposed by Becker-Weimann and co-workers [37] and the phenomenological model of calcium oscillations by Goldbeter and colleagues [38], respectively (model schemes in Fig 1A and 1B, model descriptions provided in the S1 Supplementary Information).

For these models, we find significant differences when comparing their period and amplitude sensitivity distributions (Fig 1C, p-values are zero, Tables B, C in the S1 File). The analysis reveals that the period sensitivity of the circadian model has a very narrow distribution, which implies that for every sampled parameter set the period sensitivity values are very similar (Fig 1C). In contrast, the period sensitivities in the calcium model vary broadly depending on the parameter set. For both models the amplitude sensitivities are variable (Fig 1C). The same findings are observed if the sensitivities for 75 000 instead of 2 500 parameter sets are determined with almost identical statistical characteristics of the sensitivity distributions (S1A Fig, Table N in the S1 File). Comparing both models, the period of the oscillations is systematically more sensitive to parameter perturbations in the calcium model than in the circadian model (Fig 1C and Tables B, C in the S1 File). In the calcium model, the median for the period sensitivities is eight-fold higher than in the circadian model and no overlap between the period sensitivity values of the two models exists regarding the level of 90% data ranges. Likewise, the amplitude sensitivities of the models are different (Fig 1C and Tables B, C in the S1 File). The calcium model exhibits a median amplitude sensitivity which is twice as large as that of the circadian model. In terms of the quartiles, there is no overlap of the amplitude sensitivities of both models (Table B in the S1 File). Similar results are obtained for altered sensitivity measures, i.e. if considering perturbations only in a specific subset of parameters, or if considering only the three largest sensitive coefficients for the overall sensitivity (S2 Fig).

Taken together, the analysis shows that the robustness of the period and the amplitude in both models is not exclusively determined by the choice of the kinetic parameter values. We find that the sensitivity distributions of the two examined models differ considerably. This indicates that the robustness is strongly affected also by the model structure.

Indeed, the biological processes and consequently the models of circadian and calcium oscillations (reviewed in [39,40,41,42]) differ in their core feedback type and their mass conservation properties. The core of the circadian oscillator is formed by a transcription-translation negative feedback loop (Fig 1A, line ending in T-shape with minus sign) whereas calcium oscillations rely on a positive feedback installed by calcium-induced calcium release (Fig 1B, arrow with plus sign). Circadian oscillation models are based on processes of transcription and translation leading to regulatory interactions without mass flow (dashed arrows in Fig 1A). In contrast, the transport processes that predominate in the calcium oscillator constitute mass conversions (solid arrows in Fig 1B). In addition to the two properties mentioned above the type of reaction kinetics applied in the models is of interest since it has already been shown that kinetics influences the steady state sensitivities [43] and the oscillation probability [7,32,34,44]. Therefore, we compare two major types of reaction kinetics which are used in both calcium and circadian models. First, linear reaction kinetics are described by the law of mass action, and second, saturating reaction kinetics are represented by Michaelis-Menten expressions. In the following, we examined the impact of these three structural properties on period and amplitude sensitivities. In order to foster a systematic analysis, we employed minimal prototype oscillator models.

### Positive feedback can enhance period sensitivity

**Fig 2:**
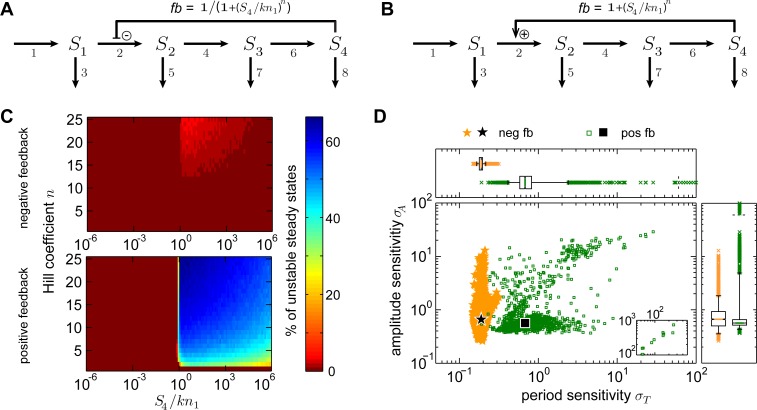
The chain model and the impact of feedback types on the sensitivity. A, B: Schemes of the prototype oscillators which are formed by an irreversible chain containing four species S_1_ to S_4_, each of which undergoes degradation. S_4_ acts on the reaction from S_1_ to S_2_ either negatively (A) or positively (B) establishing a negative or positive feedback, respectively. The according feedback terms *fb* are given above the regulatory arrow. C: Percentage of unstable steady states in dependence on *S*_*4*_*/kn*_*1*_ and the Hill coefficient *n* (according to Linear stability analysis, see Methods) for the negative feedback (A, upper panel) and positive feedback (B, lower panel) model. D: Sensitivities for the negative feedback chain model (neg fb, orange stars) and positive feedback chain model (pos fb, green squares) given in A and B, respectively.

First, we investigated the influence of the feedback type on the robustness of an oscillatory system. To this end, we considered prototype models of positive and negative feedback oscillators [29]. The models employed here describe a linear chain of four species where the last species exerts an inhibiting or activating feedback (Fig 2A or 2B, model equations provided in the S1 Supplementary Information).

A prerequisite for limit cycle oscillations is the existence of an unstable steady state. We therefore performed linear stability analyses (Methods) to examine under which conditions the chain models can exhibit sustained oscillations. We depict the percentage of unstable steady states in dependence on parameters characterizing the feedback (Fig 2C). The feedback terms *fb* (Fig 2A and 2B) are characterized by the Hill coefficient *n* and the ratio *S*_*4*_*/kn*_*1*_ which incorporates the concentration of species *S*_*4*_ and the inhibition or activation constant *kn*_*1*_. Unstable steady states occur for a Hill coefficient *n ≥ 9* for the negative feedback model (confirming results for the Goodwin oscillator [45]), and for *n ≥ 2* for the positive feedback model (Fig 2C). For both types of feedback, the percentage of parameter sets with unstable steady states is increased for larger Hill coefficients and *S*_*4*_*/kn*_*1*_ *≥ 0*.*7*. In the following, we therefore set the Hill coefficients to *n* = 9 and *n* = 2 for the negative and positive feedback chain models, respectively. We validated our sensitivity results for Hill coefficients *n = 9* in both models (S3 Fig).

For both chain models, we performed sensitivity analyses following the established work-flow (see Methods). The sensitivities of the positive and negative feedback model segregate into two populations (Fig 2D). The negative feedback chain model exhibits consistently low period sensitivities in a narrow range (median period sensitivity 0.19, 90% data range 0.05, Table D in the S1 File) but quite variable amplitude sensitivities (median amplitude sensitivity 0.66, 90% data range 1.49, Table D in the S1 File). The positive feedback chain model displays significantly higher period sensitivities in a broader range (median period sensitivity 0.68, 90% data range 1.99, p-value is zero, Tables D, E in the S1 File). The amplitude sensitivity is significantly lower in the positive feedback chain model than in the negative feedback chain model (decrease of the median value by 16%, p-value <10^−5^, Tables D, E in the S1 File) but covers a broader range (90% data range 4.48). Interestingly, the positive feedback chain model yields a subset of parameters characterized by a very low amplitude sensitivity (interquartile range of 0.06 around the median of 0.57 for the amplitude sensitivity distribution, Table D in the S1 File) while the period sensitivity is variable.

We next analyzed the underlying reasons for the observed sensitivity differences between the negative and positive feedback chain model. We chose to track the dynamical behavior of both models for selected parameter sets by bifurcation analyses. Such analyses allow the study of changes in the period and amplitude for a continuous alteration of one selected parameter, the so-called bifurcation parameter. We selected the parameter which most strongly affected the period (i.e. the parameter with highest |R^T^|) to be the bifurcation parameter. For the negative feedback chain model, the three bifurcation diagrams (S4A–S4C Fig, according to marks A-C in S4D Fig) show only slight variations of the period and smooth variations of the amplitude. For the positive feedback chain model, for each of the four parameter sets (S5A–S5D Fig, according to marks A-D in S5E Fig) the variability of the period strongly changes with the value of the bifurcation parameter. The strongest variations in the amplitudes are found for bifurcation parameter values close to the bifurcation points, while the amplitude changes considerably less elsewhere. Additionally, the bifurcation analysis reveals the possibility of transitions between limit cycles with different periods and amplitudes for bifurcation parameter changes (S5A–S5C Fig) which opens the possibility for the occurrence of high sensitivities as realized in S5C Fig, but not in S5A and S5B Fig.

Overall, although we chose parameter sets with different sensitivity values for the bifurcation analyses, the bifurcation diagrams are similar among those for the negative feedback chain model and among those for the positive feedback chain model. However, comparing the models, the bifurcation diagrams differ considerably emphasizing the importance of the feedback type for the variability in the dynamical behavior and thus also for the sensitivities of the models.

Altogether, investigating the chain models we observe that a negative feedback leads to low period sensitivities whereas a positive feedback enables higher and more variable period sensitivities. Amplitude sensitivities are highly variable for the negative feedback chain model, but less variable in terms of the interquartile range and overall lower for the positive feedback chain model.

### Using Michaelis-Menten kinetics can increase sensitivities

Having assessed the influence of the feedback type on the sensitivities, we then focused on the effect of the type of reaction kinetics as further important feature of models of biological networks. We here considered mass action kinetics with reaction rates *ν = k · S*, and Michaelis-Menten kinetics with reaction rates *ν = V · S/(S + K*_*M*_*)*.

**Fig 3:**
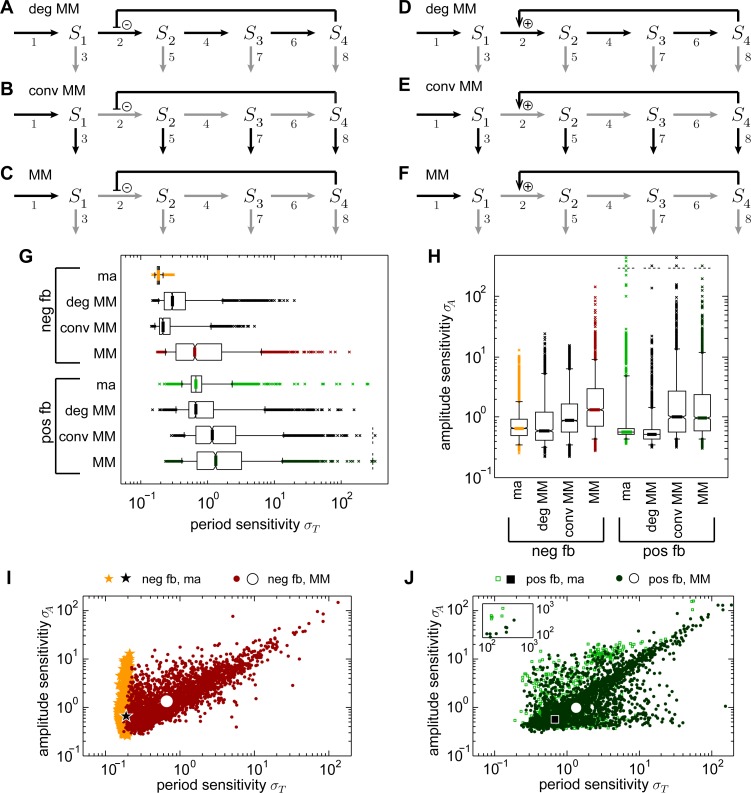
Impact of the reaction kinetics on the sensitivity of the chain model. A-F: Schemes of the chain models with negative (A-C) or positive (D-F) feedback employing Michaelis-Menten kinetics in degradation reactions 3, 5, 7, 8 (A, D, indicated in gray, deg MM), in conversion reactions 2, 4, 6 (B, E, indicated in gray, conv MM), or in conversion and degradation reactions 2–8 (C, F, MM). G, H: Box-plots of the period sensitivities (G) or amplitude sensitivities (H) of the chain models with negative and positive feedback. I, J: Sensitivities of the negative feedback (I, neg fb, dark red dots) or positive feedback (J, pos fb, dark green dots) chain model with Michaelis-Menten kinetics in reactions 2–8 as shown in C or F, respectively. For comparison the data for the models with mass action kinetics (ma) in reactions 2–8 from Fig 2D are shown in addition.

To study their effect on period and amplitude sensitivity, we adapted the chain models with positive and negative feedback which have been described with mass action kinetics so far by introducing Michaelis-Menten kinetics (marked in gray in Fig 3A–3F, equations given in the S1 Supplementary Information). We considered three different scenarios: Replacing exclusively the degradation reactions 3, 5, 7, 8 (Fig 3A and 3D), replacing exclusively the conversion reactions 2, 4, 6 (Fig 3B and 3E), and replacing all reactions but the production rate of species S_1_ (Fig 3C and 3F).

The results of the sensitivity analyses for these modified chain models are compared among each other and to those for the corresponding models with mass action kinetics (Fig 3G–3J). In the majority of cases, we observe larger medians and distribution ranges for the period and amplitude sensitivities for the chain models with Michaelis-Menten kinetics than with mass action kinetics for both types of feedback. In particular, the median period sensitivities are significantly increased compared to the corresponding chain model with mass action kinetics (between 1.2- and 3.5-fold, Fig 3G, p-values <10^−5^, Tables F, G in the S1 File), except for the positive feedback model with Michaelis-Menten kinetics only in the degradation reactions for which no significant change is revealed (p-value 0.29, Table G in the S1 File). Whether the median amplitude sensitivities increase or decrease depends on the type of reaction that employs Michaelis-Menten instead of mass action kinetics. Compared to the respective model with mass action kinetics, amplitude sensitivities are slightly decreased by 1.1-fold for both feedback types if exclusively the degradation reactions obey Michaelis-Menten kinetics (Fig 3H, p-values <6·10^−3^, Tables F, G in the S1 File). If conversion reactions obey Michaelis-Menten kinetics (Fig 3B, 3C, 3E and 3F), the amplitude sensitivities are increased compared to the corresponding model with mass action kinetics (between 1.3- and 2-fold, Fig 3H, p-values <10^−5^, Tables F, G in the S1 File). The distribution widths of the period and amplitude sensitivities are enlarged if one compares the models with mass action kinetics to those employing Michaelis-Menten kinetics (between 2.5- and 126-fold enlarged 90% data ranges, Fig 3G and 3H, Table F in the S1 File). The only exception is made by the positive feedback chain model with Michaelis-Menten kinetics only in the degradation reactions for which a 4-fold reduction of the width of the amplitude sensitivity is observed (Fig 3D and 3H, Table F in the S1 File). The comparison of the effect of the change to Michaelis-Menten kinetics in the different reaction types shows that for the negative feedback chain model, the period sensitivities are more strongly affected if altering the kinetics in the degradation reactions than in the conversion reactions. The opposite is observed for the amplitude sensitivities. For the positive feedback chain model, altering kinetics in conversion reactions has a dominant increasing effect for both period and amplitude sensitivities.

Employing Michaelis-Menten kinetics instead of mass action kinetics leads to the introduction of additional parameters, the K_M_-values, in a model. An analysis of the sensitivity coefficients shed light on the contribution of each single parameter to the overall sensitivity in the chain model with positive or negative feedback and either mass action or Michaelis-Menten kinetics in all degradation and conversion reactions (S6 Fig). Larger sensitivity coefficients indicate stronger influence of the corresponding parameter. For the models with Michaelis-Menten kinetics, we observe that the K_M_-values have a smaller impact on the periods and amplitudes than their corresponding rate coefficients (S6 Fig, compare the last two box-plots for each triple). Comparing the sensitivity coefficients of the rate coefficients between models with different kinetics, we observe an increase for the models employing Michaelis-Menten kinetics (S6 Fig, compare the first two box-plots for each triple). Hence, the increase in the sensitivities in the models with Michaelis-Menten kinetics does not solely result from the introduction of the K_M_-values but we also observe an increase in the sensitivities of the rate coefficients.

In total, using Michaelis-Menten instead of mass action kinetics in all reactions leads to increased period and amplitude sensitivities for the negative as well as positive feedback chain model. In addition, increased ranges of the sensitivity distributions in models with Michaelis-Menten kinetics depict a stronger dependence of the sensitivities on the choice of the parameter set which are found in all models except for the amplitude sensitivity in the model from Fig 3D. Employing Michaelis-Menten kinetics only in degradation or conversion reactions mostly leads to a weaker increase in the sensitivities than employing this kinetics in both.

### Mass conversion can moderately decrease sensitivities

**Fig 4:**
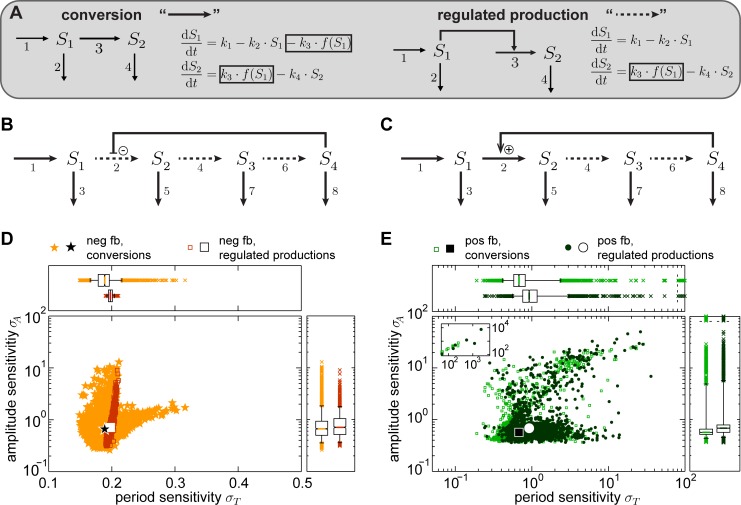
Impact of mass conservation properties on the sensitivity of the chain model. A: Schemes and equations of a mass conversion and a regulated production rate (reaction 3). For a mass conversion, the reaction rate occurs in the equation of the source species as well as of the product species (highlighted by boxes). For a regulated production rate, only the equation of the product species is affected by the reaction (highlighted by box). Regulated production rates are in the following represented by dashed arrows, mass conversions by solid lines. B, C: Schemes of the chain model with negative (B) or positive (C) feedback in that mass conversion reactions 2, 4, 6 or 4, 6, respectively, have been replaced by regulated production rates. D, E: Sensitivities for the negative (D, neg fb, red squares) and positive (E, pos fb, dark green dots) feedback chain model as shown in B and C, respectively. For comparison, sensitivities considering mass conversion reaction rates only are shown (data from Fig 2D).

We further investigated whether the mass conservation properties in a model affect its robustness with respect to period and amplitude. The two qualitatively different types of reactions are mass conversions and regulatory interactions without mass flow, referred to as regulated production rates (Fig 4A). A mass conversion mediates a mass flow from the source species of the reaction to the product species. Thereby, the source species are consumed via the reaction. Examples are metabolic processes, transport processes and modifications like phosphorylations or methylations, in which the unmodified species’ concentrations are decreased while the concentrations of the modified species increase. In contrast, regulated productions imply an information transfer, but no mass conversion, between the regulating and regulated species. Examples are translation of mRNA into protein, or kinases mediating a phosphorylation without changing their own concentration.

So far, the chain models employed mass conversions for reactions 2, 4 and 6. In the negative feedback chain model, we replaced these three mass conversions with regulated production rates (Fig 4B, dashed arrows indicate regulated production rates, model equations given in the S1 Supplementary Information). In the positive feedback chain model, only reactions 4 and 6 were replaced by regulated productions (Fig 4C, dashed arrows indicate regulated production rates). If reaction 2 is considered to be a regulated production rate in this model, it is not a substrate-depletion oscillator anymore and sustained oscillations do not occur.

For the two modified models we performed sensitivity analyses following the established work-flow (see Methods). Substituting mass conversions by regulated production rates renders the period as well as amplitude more sensitive to parameter perturbations for both feedback types (Fig 4D and 4E). For both models median period and amplitude sensitivities are increased between 1.05- and 1.37-fold (p-values <10^−5^, Tables H, I in the S1 File). The effects of employing regulated productions instead of mass conversions on the level of the individual sensitivity coefficients differ for the chain models with negative and positive feedback (S7 Fig). The ranges of the period and amplitude sensitivities become smaller for the model with regulated productions for the negative feedback, but larger in case of the positive feedback (compare values of the 90% data ranges in Table H in the S1 File).

Differences in the sensitivities introduced by employing regulated production rates instead of mass conversions remain significant but small compared to the differences originating from the different types of feedbacks or kinetics (Figs 2D and 3).

The sensitivities of the chain models including the combinations of feedback type, kinetics and mass conservation properties are summarized in S8 Fig. We considered only the cases of mass action kinetics or Michaelis-Menten kinetics in all reactions. The data for models with negative feedback N1-N3 and positive feedback P1-P3 correspond to those presented in Figs 2D, 3I, 3J, 4D and 4E. They allow for the comparison between chain models with negative and with positive feedback (N1, P1), reactions with mass action and with Michaelis-Menten kinetics in models with both feedback types (N1 to N2 and P1 to P2), or with mass conversions and with regulated production rates in negative and positive feedback models (N1 to N3 and P1 to P3). Additionally, we also analyzed the effect of employing Michaelis-Menten kinetics in combination with regulated production rates in both feedback models (N4, P4 in S8A Fig). The comparison of N3 to N4 and P3 to P4 shows a strong increase in the sensitivities if using Michaelis-Menten instead of mass action kinetics. Hence, the impact of the type of kinetics on the sensitivities is strong and appears independent of the considered assumption on mass conservations. Comparing N2 to N4 and P2 to P4 demonstrates that if employing Michaelis-Menten kinetics, the introduction of regulated production rates instead of mass conversions has rather moderate effects on the period sensitivities. The amplitude sensitivities are influenced, however, less than if changing the reaction kinetics (compare N1 to N2, P1 to P2, N3 to N4, P3 to P4).

### Analysis of further circadian and calcium oscillations models

In order to test whether our insights for the chain models are valid beyond prototype oscillators, we analyzed established models of circadian and calcium oscillations in addition to the two models that have been examined initially (Fig 1). For circadian oscillations, we selected the model for *Drosophila melanogaster* proposed by Goldbeter and colleagues [46] and the model for *Arabidopsis thaliana* by Locke and co-workers [47]. For calcium oscillations, we examined the models published by Sneyd and co-workers [48] and by De Young and Keizer [49] in order to represent an open cell model and a closed cell model (equations of all four models are provided in the S1 Supplementary Information).

**Fig 5:**
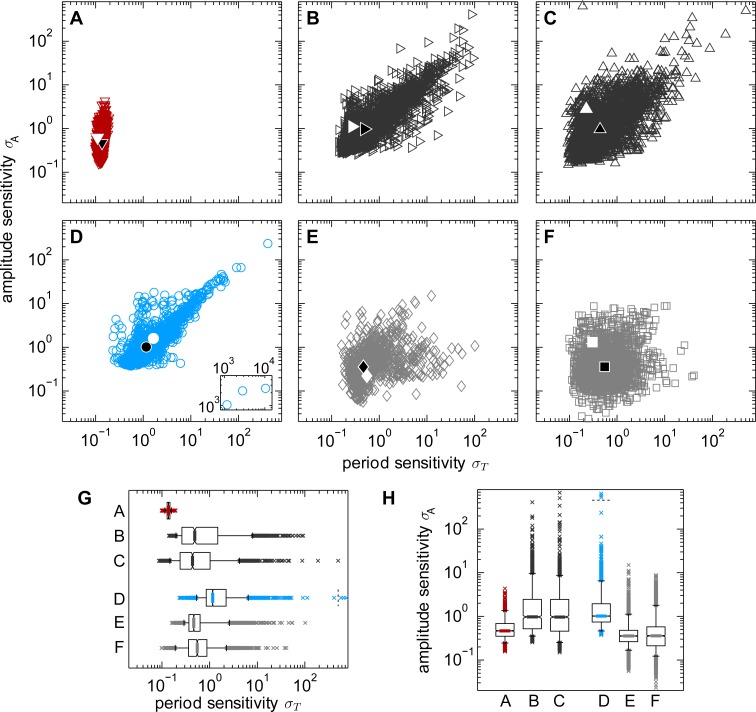
Sensitivities for models of circadian and calcium oscillations. A-C: Circadian models. (A) Model for mammalian cells [37] (compare Fig 1A and 1C red triangles); (B) model for *D*. *melanogaster* [46]; (C) model for *A*. *thaliana* [47]. D-F: Calcium models. (D) Phenomenological model [38] (compare Fig 1B and 1C blue circles); (E) open-cell model [48]; (F) closed-cell model [49]. Black symbols denote median values, white symbols the sensitivities for the parameter set published together with the model. G, H: Box-plots of the period (G) and the amplitude (H) sensitivity distributions for the models from A-F. The schemes and further details of the models are given in Fig 6.

First, the sensitivities of the circadian oscillation models are compared (Fig 5A–5C, 5G and 5H). We observe that only the mammalian circadian rhythm model (Fig 5A, see also Fig 1C) shows a low period sensitivity for almost all parameter sets. The circadian models for *D*. *melanogaster* (Fig 5B) and *A*. *thaliana* (Fig 5C) exhibit larger median period sensitivities and decisively larger distribution widths of the period sensitivities (Tables J, K in the S1 File). Similar results are obtained for the amplitude sensitivities. Hence, in those models, the sensitivities can vary depending on the chosen parameter set. Interestingly, the reference parameter sets published together with the models give rise to low period sensitivities (white symbols in Fig 5B and 5C).

**Fig 6:**
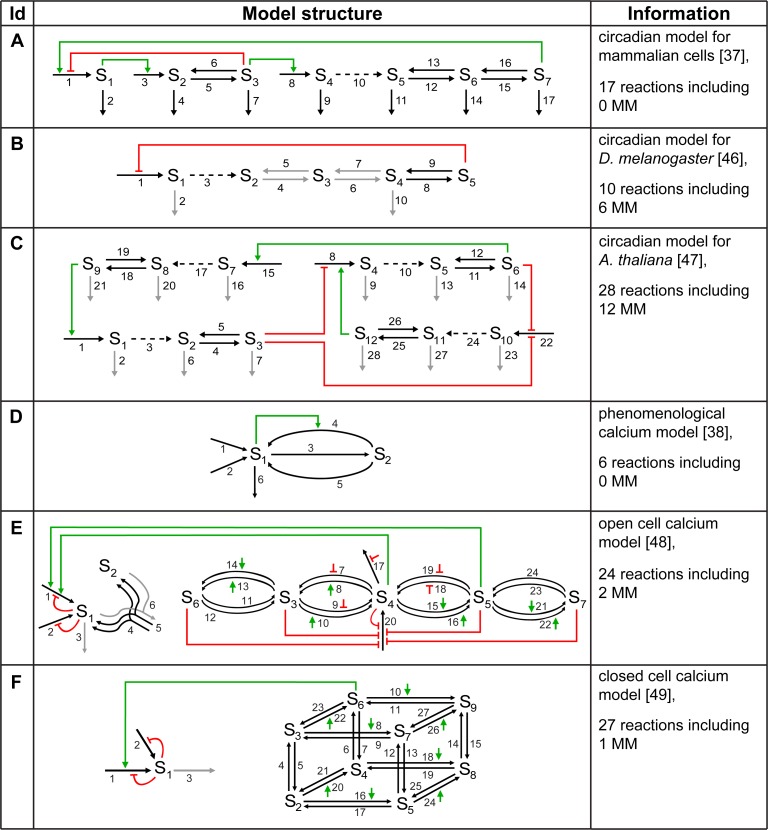
Model structures of the circadian and calcium oscillations models examined in Fig 5. The column ‘Id’ gives the identifier of the model according to Fig 5. In the model structure column, black and gray arrows denote reactions in the models, reactions with Michaelis-Menten kinetics (MM) are thereby marked in gray. Dashed arrows represent regulated productions. Red lines ending in T-shape indicate negative regulations, green arrows denote positive regulations. Green or red arrows without source (in E, F) represent regulations by species S_1_ (cytosolic calcium) on the according reaction.

We addressed the question whether the increased and more variable period and amplitude sensitivities can be explained applying the insights obtained from the analyses of the chain models. Therefore, we investigated the model structure of the circadian rhythm models with respect to the feedback type and reaction kinetics (Fig 6). We did not examine the mass conservation properties due to the rather moderate effect of regulated productions versus mass conversions on the sensitivities observed for the chain models (Fig 4D and 4E).

The sensitivity characteristics of the mammalian circadian rhythm model resemble that of the chain model with negative feedback and mass action kinetics (compare Fig 5A to the orange stars in Fig 2D). Indeed, the circadian model employs a negative feedback (negative regulation on reaction 1, Fig 6A) and mass action kinetics for all reactions except for the regulatory terms. In addition, a positive feedback acts on production reaction 1. In that case, the positive feedback does not establish a substrate-depletion mechanism which would lead to an increase of the sensitivities according to our chain model analysis (Fig 2D). Here, however, the positive feedback seems not to influence the sensitivities.

The model for circadian oscillations in *D*. *melanogaster* relies on a negative feedback (Fig 6B) and frequently employs Michaelis-Menten kinetics in both conversion as well as degradation reactions (gray arrows in Fig 6B). Thus, the model is comparable to the chain model with negative feedback applying Michaelis-Menten kinetics in all reactions (compare Fig 5B to the dark red dots in Fig 3I). From these model structure properties one would expect considerably larger median period and amplitude sensitivity values and ranges for the *D*. *melanogaster* model compared to the mammalian circadian model which are in fact obtained (compare Fig 5B to Fig 5A, p-values <10^−5^, Tables J, K in the S1 File). The circadian model for *A*. *thaliana* encompasses positive and negative regulations which result in only negative feedbacks (Fig 6C), and all degradation reactions obey Michaelis-Menten kinetics (gray arrows in Fig 6C). Thus, it resembles the negative feedback chain model with Michaelis-Menten kinetics in the degradation reactions (Fig 3A, 3G and 3H). One would expect enlarged median period sensitivities and distribution ranges and slightly decreased amplitude sensitivities compared to the mammalian circadian model. We observe the former, but also increased amplitude sensitivities (compare Fig 5C to Fig 5A, p-values <10^−5^, Tables J, K in the S1 File), which could result from the overall different structures of the two models (Fig 6A and 6C). Taken together, in most cases we can explain the sensitivity distributions of the examined circadian models [37,46,47] using insights gained from the analyses of the chain models. Like in the prototype oscillator models the use of Michaelis-Menten kinetics influences the sensitivity distributions of circadian oscillation models.

The median period sensitivities of the calcium oscillation models (Fig 5D–5G) are high compared to those of the mammalian circadian model (Fig 5A, p-values are 0, Table K in the S1 File). Among the three calcium models, the initially chosen model (Fig 5D) shows the highest median period and amplitude sensitivities (Fig 5D, 5G and 5H, p-values are 0, Table K in the S1 File). The median period sensitivities of the open and closed cell calcium models (Fig 5E and 5F) are similar to that of the circadian model of *D*. *melanogaster* (Fig 5B and 5G, p-values 0.13 and 0.20, Table K in the S1 File) while their median amplitude sensitivities are lower than for all other examined models (Fig 5H, p-values <3·10^−5^, Table K in the S1 File). The reference parameter sets published with the three calcium models have not been selected for high or low period sensitivity and lead to values above as well as below the median values (compare white and black symbols in Fig 5D–5F).

We analyzed the structures of the calcium models in order to elucidate the underlying reasons for the observed sensitivity distributions. The phenomenological calcium model (Fig 6D, Fig 5D) includes a positive regulation acting on a conversion reaction thus establishing a substrate-depletion motif. This motif has been shown to result in increased period sensitivities (Fig 2D) giving an explanation for the high period sensitivities in the calcium model compared to the mammalian circadian model (Fig 5A).

The other two calcium oscillation models exhibit regulatory structures very different from those of the chain models (Fig 6E and 6F). Most variables of the open cell and the closed cell model represent opening probabilities of receptor states leading to highly connected models. Cytosolic calcium occurs in 17 out of 24 reactions for the open cell model and in 11 out of 27 reactions for the closed cell model. Consequently, explaining the obtained sensitivities based on the examinations of the lowly connected chain models is difficult. Indeed, the period and amplitude sensitivity distributions of these calcium models do not resemble any of the patterns observed in the chain models (Fig 5E and 5F).

Hence, insights obtained from the chain models can be used to explain the calculated sensitivity distributions of the circadian models and the phenomenological calcium model. In contrast, conclusions for the distributions computed for the open and closed cell calcium models cannot be drawn because their structures strongly differ from those of the analyzed chain models.

### Analysis of additional models of biological oscillations

**Fig 7:**
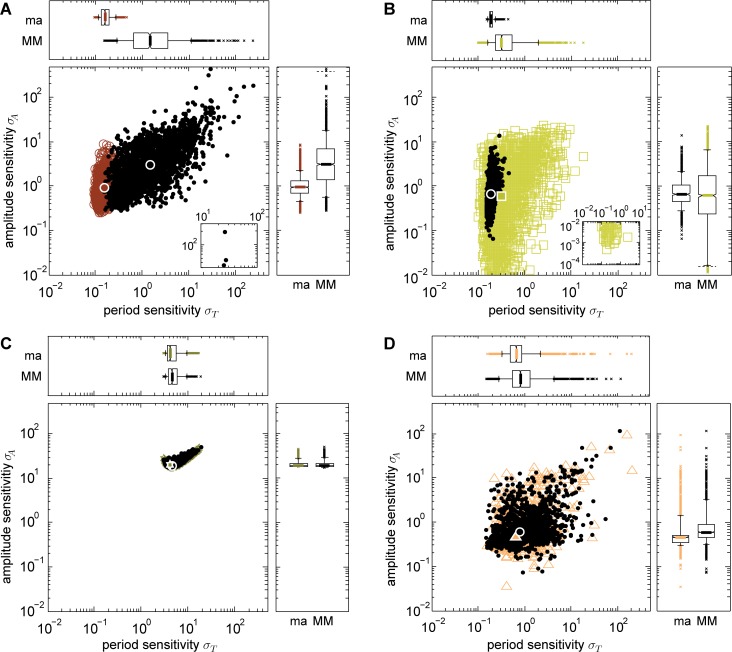
Sensitivities of models for various biological oscillations. A: Model of the repressilator [50] (brown circles). B: Model of the MAPK pathway [51] (yellow squares). C: Model of the glycolysis [52,53] (olive stars). D: Model of the cell cycle [54] (salmon triangles). White framed symbols denote median values. The schemes and further details of the models are given in S9 Fig. The originally published kinetics were altered employing Michaelis-Menten kinetics instead of mass action kinetics in A, C, D and mass action kinetics instead of Michaelis-Menten kinetics in B (black dots, black circle as median values). ma: mass action kinetics, MM: Michaelis-Menten kinetics.

For the chain models we have demonstrated that employing Michaelis-Menten instead of mass action kinetics can result in both increased medians and increased distribution widths of the period and amplitude sensitivities (Fig 3), and made similar observations for models of circadian oscillations (Fig 5). Here, we investigated whether this also holds beyond the chain models and the models of circadian oscillations. Therefore, we chose further models of different biological oscillations and analyzed their sensitivities using the originally published type of reaction kinetics. In addition, we replaced all kinetics with the respective other type and compared the resulting sensitivity distributions. We selected the repressilator model [50] (sensitivities in Fig 7A) and a model of MAPK signaling [51] (sensitivities in Fig 7B) representing negative feedback oscillators and models describing the glycolysis [52,53] (sensitivities in Fig 7C) and the cell cycle [54] (sensitivities in Fig 7D) as representatives for substrate-depletion oscillators, model structures are given in S9 Fig, equations of the models are provided in the S1 Supplementary Information. In the original publications, the MAPK model employs Michaelis-Menten, all other three models consider mass action kinetics.

Also in these models of biological oscillations, the employed type of kinetics has an impact on the medians and the distribution widths of the period and amplitude sensitivities. If Michaelis-Menten instead of mass action kinetics are considered, median period sensitivities increased in all four oscillator models (between 1.1- and 9.3-fold, Fig 7A–7D, p-values <10^−5^, Tables L, M in the S1 File). Median amplitude sensitivities increased 3.3-fold for the repressilator (Fig 7A) and 1.3-fold for the cell cycle model (Fig 7D) but slightly decreased for the MAPK and the glycolysis models (1.1- and 1.02-fold, respectively, p-values <3·10^−4^, Tables L, M in the S1 File). For the repressilator, the MAPK and the cell cycle model, the distribution widths of both the period and the amplitude sensitivities enlarged if Michaelis-Menten instead of mass action kinetics are used (between 2.1- and 70-fold increased 90% data ranges, Table L in the S1 File). Regarding the glycolysis model, the distribution width of the period sensitivity is decreased 1.04-fold while the width of the amplitude sensitivity distribution is increased 1.4-fold (Table L in the S1 File).

Hence, for the four considered models of biological oscillations the conclusion holds that employing Michaelis-Menten instead of mass action kinetics leads to increased median period sensitivities, and to increased period sensitivity distribution widths (except for the glycolysis model). Also, the widths of the amplitude sensitivity distributions enlarged for all four models which agrees with the corresponding observations in the chain models. Regarding the amplitude sensitivities, the analyses of the chain models have already revealed that they are differently affected depending on the type of reaction whose mass action kinetics is replaced by Michaelis-Menten kinetics (Fig 3). We also found ambiguous effects for the four additional models of which two show increased median amplitude sensitivities (Fig 7A and 7D), two show decreased median amplitude sensitivities (Fig 7B and 7C). All in all, also in the models of various biological oscillations, the employed type of reaction kinetics strongly influences the sensitivity distributions.

## Discussion

In this work, we studied the robustness of period and amplitude of cellular oscillations by investigating a variety of model systems known to exhibit sustained oscillations. In order to distinguish properties that are inherent to the model structure from those resulting from the choice of parameter values, we examined the sensitivities for 2 500 randomly sampled parameter sets for each of the models.

**Fig 8:**
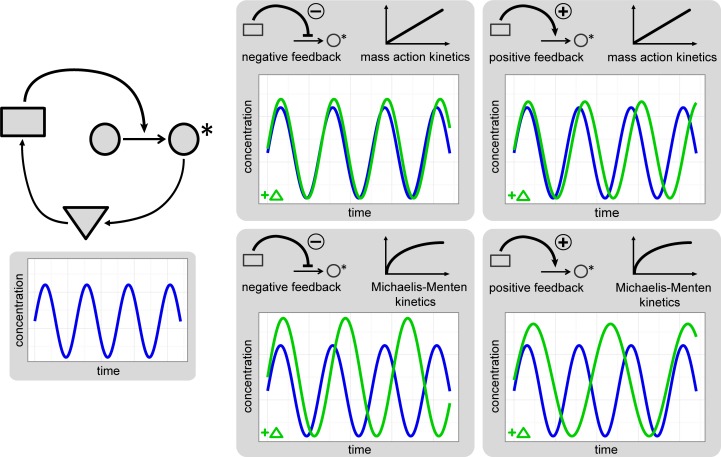
Effect of structural characteristics on the sensitivities. Schematically, the effect of negative feedback (left) or positive feedback (right) and mass action kinetics (upper) or Michaelis-Menten kinetics (lower) on the period and amplitude sensitivities are depicted. The respective sensitivities are indicated by how much the period or amplitude of the perturbed system (oscillation for an example perturbation +Δ is shown in green) deviate from these characteristics in the unperturbed system (blue).

To investigate the impact of structural characteristics on robustness, we used prototype oscillator models which allow for a dissection of the influence of feedback type, reaction kinetics and mass conservation properties. In many studies, prototype models have successfully been used to analyze the impact of design principles on system characteristics [24,55]. Very often, minimal models are used as a first approach to describe new biological observations (e.g. [31,52,56]) implying that our insights can be directly used to design models with specific robustness properties. Using our prototype models, we found the feedback type and the reaction kinetics to be of major importance for the period and amplitude sensitivities, illustrated in Fig 8. Employing positive feedback instead of negative feedback leads to an increase in the period sensitivities. If Michaelis-Menten kinetics instead of mass action kinetics are considered in all conversion and degradation reactions, the period and amplitude sensitivities are increased. Thereby, the different types of reactions exert different impacts. The period sensitivities are influenced moderately by the mass conservation properties, whereas the amplitude sensitivities are altered to a similar extent as by feedback types and reaction kinetics. We found these conclusions on the effect of feedback type and kinetics to be valid when extending the analysis to several representative models of circadian and calcium oscillations as long as the model structure bears some resemblances to the prototype models. The effect of the type of the kinetics on the period sensitivities was also confirmed for further models of various biological oscillations.

Previous computational approaches have demonstrated that saturating kinetics in degradation reactions can increase the oscillation probability, especially in negative feedback oscillators [7,34,44]. Taking this effect of saturating kinetics together with its herein observed effect of increasing the period sensitivities might imply a robustness trade-off between different characteristics of an oscillating system: the higher robustness of periods for negative feedback models with linear reaction kinetics may come at cost of a smaller region in the parameter space in that the systems exhibit oscillations.

Our results for the period and amplitude sensitivity can be compared to those obtained for the tunability of these characteristics in oscillating models relying on a negative feedback with or without an additional positive feedback [24]. In the former study, an individual parameter has been varied over a broad range and changes in period and amplitude have been followed. It has been found that the negative feedback model delivers stable periods and tunable amplitudes while adding a positive feedback results in tunable periods and stable amplitudes [24]. Despite the different feedback implementations and robustness measures, those results and the results of our study are in agreement (compare Fig 2D). Since our approach examines small changes in all parameters, it avoids bias towards the choice of varied parameters. As both approaches result in comparable observations the conclusion is emphasized that indeed the model structure significantly affects period and amplitude sensitivities.

While we observe overall low amplitude sensitivities in the positive feedback chain model, a detailed inspection of Fig 2D also reveals parameter sets with high amplitude sensitivities. Interestingly, in the previously published study [24] only low amplitude sensitivities have been found in the model including a positive feedback. This might result from the fact that therein the maximal amplitude has been defined as a parameter which has been set to unity. During the sampling procedure it has not been varied which might lead to an underestimation of the variation in the amplitude in their investigation.

In order to select parameter sets for each of the examined models, we used a bottom-up sampling approach. Thereby the steady state concentrations and flows were sampled and the rate coefficients were calculated from them. This method allows a very fast detection of steady state stability and the exclusion of parameter sets not yielding sustained oscillations. In terms of computational costs, it therefore outperforms top-down approaches in which kinetic parameters are sampled and steady state stability is not determined (for details see Methods). Moreover, in systems in which steady state concentrations and flows of participating species are more reliably characterized than reaction rate coefficients, it is more straightforward to sample these values in the determined intervals. For the examination of the period and amplitude sensitivities, the results remained unaffected by the specific sampling interval due to the consideration of relative changes (see Methods). We here assumed that the sampling intervals for concentrations and K_M_-values or inhibition and activation constants are similar which enables the occurrence of all possible regulatory modes of non-linear regulatory terms (see Methods).

Overall, our results extend an earlier study in which only the feedback type has been held responsible for the sensitivities of period and amplitude [24]. We here demonstrate that both the feedback type and the reaction kinetics influence the range of the sensitivity values considerably. Consequently, the model structure determines which influence the choice of the parameter set can have on the sensitivities. In the light of evolution this might be important for biological rhythms being subject to different constraints with respect to their input-output characteristics. For example, the negative feedback oscillator structure, together with linear reaction kinetics, enables the occurrence of low period sensitivities regardless of the choice of the parameter set. Thus, the parameter values of the model representing reaction velocities and binding constants can be tuned such that the oscillator can exhibit other important characteristics, as e.g. entrainability by light cues in the case of circadian rhythms. In contrast, substrate-depletion oscillators and oscillators with saturating kinetics display wide period and amplitude sensitivity distributions. This opens the possibility that these oscillators show different period and/or amplitude sensitivities and thus satisfy cell-type-, tissue- or organism-specific demands by only adapting reaction velocities while keeping the wiring of the participating species unchanged.

Our approach can be extended in multiple directions. First, it might be of interest to elucidate the impact of additional design principles of the model structure on the sensitivities. Earlier studies have shown that also the delay affects the occurrence of oscillations as well as the obtained amplitudes and periods [34,57,58,59]. The role of delay on the sensitivity of the period for parameter perturbations has been examined for one parameter set in the chain model [29] for which increasing delay (i.e. increasing chain length) decreased period sensitivities. It would be interesting to investigate whether the effects of delay hold for the models for many randomly sampled parameter sets. Second, the work-flow is not restricted to the analysis of models based on the description of biochemical reactions but can also be applied to models developed to generate oscillations with specific properties. This allows dissecting the influence of characteristics not captured in biochemical-based models or to expand the analyses towards more theoretical aspects determining the specific oscillatory behavior. As proof of concept, we computed the sensitivities of the FitzHugh-Nagumo model of neural dynamics [60] and of a specific λ-ω system [61]. The FitzHugh-Nagumo model delivers high period and low amplitude sensitivities, the selected λ-ω system shows variable period sensitivities and nearly constant, low amplitude sensitivities (S10 Fig).

Furthermore, in the last years, new measurement techniques have elucidated oscillations on the level of single cells which underlie intrinsic fluctuations, stressing the importance to analyze stochastic models. The effect of noise on the robustness of oscillations has been computationally studied so far for a model of calcium oscillations showing that the sensitivity of the period towards external signals can be reduced by intrinsic noise [62]. Moreover, an enhancing effect of intrinsic fluctuations on the occurrence of oscillations has been demonstrated for circadian models and the NF-κB system [63,64,65].

## Methods

**Fig 9:**
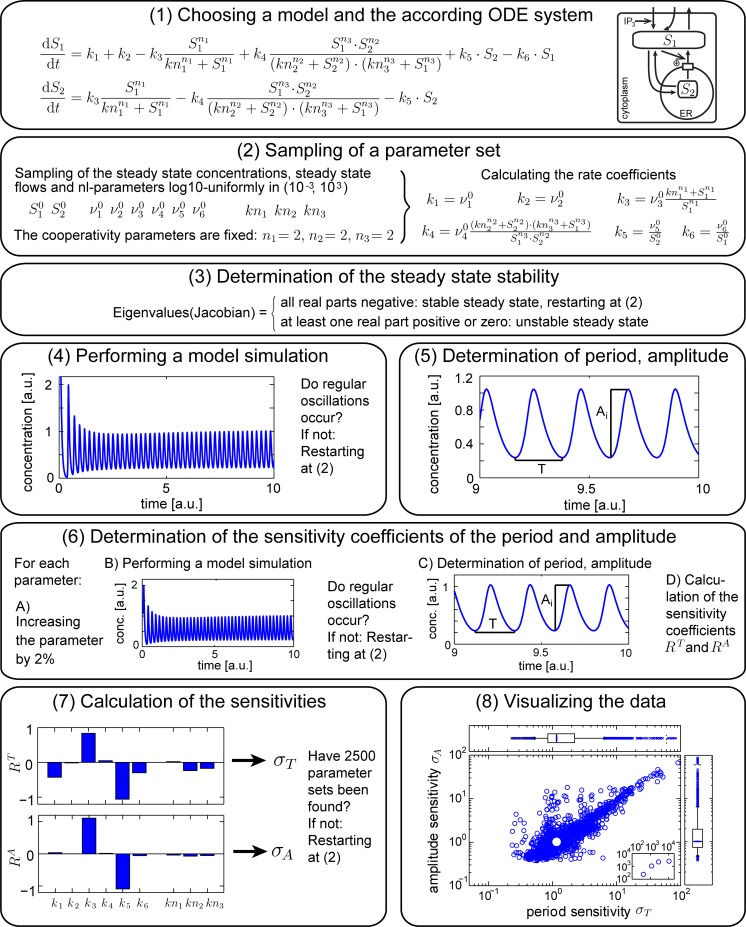
Work-flow of the analysis for the example of a calcium oscillations model [38]. The analysis can be divided into eight parts. Details are described in Methods.

The work-flow of the analysis is shown in Fig 9 using the model of Goldbeter et al. [38] as an example. Details of each part are given in the following.

### Mathematical models (Fig 9, box 1)

For each examined oscillatory process a mathematical model given by an ordinary differential equation (ODE) system was chosen. Other model approaches such as network analysis, stoichiometric analysis and structural kinetic modeling do not provide enough detail with respect to the oscillatory properties [66].

In the ODE approach the concentration *S*_*i*_ of the *i*th intermediate is determined by

$$\frac{dS_{i}}{dt}=\sum_{j=1}^{M}\eta_{ij}\nu_{j}$$ (Eq. 5)

for *i = 1*,*…*, *m*, with *m* the number of variables, *η*_*ij*_ the stoichiometric coefficients and *M* the number of flows *ν*_*j*_. The flows *ν*_*j*_ are always positive. Their magnitude is determined by the kinetic parameters and intermediate concentrations. We here distinguished between three types of parameters: (i) Each flow has a corresponding rate coefficient that enters linearly, e.g. maximal reaction velocities. The flows can also depend non-linearly on the intermediate concentrations and kinetic parameters. Those parameters can be either (ii) cooperativity parameters, e.g. a Hill-coefficient, defining the non-linearity of the rate laws, or (iii) kinetic parameters such as Michaelis-Menten constants, which are called nl-parameters.

All model parameters are considered dimensionless since for each model system an appropriate non-dimensionalization is used.

### Parameter sampling (Fig 9, box 2)

In order to allow a comparison of models of biological processes acting on various time scales and in different concentration ranges an unbiased parameter sampling approach is necessary. During the sampling procedure we did not vary the characteristics of the mechanistic interactions between species, that is, stoichiometry, cooperativity parameters, and functional form of the rate laws are not altered.

In order to derive sets of kinetic parameters we employed a bottom-up approach: We performed a random sampling of steady state concentrations, flows and nl-parameters. Each of the sampled sets characterizes a unique state of the model. In principle, the random sampling procedure allows to obtain all possible states. From each set of sampled steady state concentrations, flows, and nl-parameters the according set of rate coefficients was directly calculated.

In detail, we sampled the steady state concentrations in the range (10^−3^, 10^3^) such that the decadic logarithms are uniformly distributed (referred to as log10-uniform distribution in the following). We used the log10-uniform instead of a uniform distribution to take into account that intermediate concentrations within one system may occur at very different orders of magnitude [67]. The log10-uniform distribution allows for an equal representation of each of the seven orders of magnitude for the intermediate concentrations. For variables denoting probabilities in the calcium models [48,49], we adapted the interval to (10^−3^, 1).

Next, we chose the steady state flows such that each flow is in the interval (10^−3^, 10^3^) and the steady state condition

$$0=\sum_{j=1}^{M}\eta_{ij}\nu_{j}$$ (Eq. 6)

is fulfilled for each variable *i = 1*,*…*, *m*. In all investigated models, the number of flows, *M*, is higher than the number of variables, *m*. Therefore, the system of *m* equations and *M* unknowns given by the steady state condition (Eq 6) yields (*M-m)* linearly independent flows. Those were chosen in a random manner from the *M* flows and sampled log10-uniformly in the interval (10^−3^, 10^3^), details given in S11 Fig. From the (*M-m*) sampled flows we computed the *m* depending flows. If necessary, the sampling was repeated until all of the computed flows reached a value in the interval (10^−3^, 10^3^).

Finally, having determined steady state concentrations and flows, we sampled all nl-parameters log10-uniformly in the range (10^−3^, 10^3^).

From the sampled values we calculated the rate coefficients using the equations defining the flows (for example, see the equations for the calcium model by Goldbeter and colleagues [38] in the S1 Supplementary Information). The set of rate coefficients can be derived as a unique solution since each rate coefficient depends linearly on its corresponding flow.

Note that while our approach ensures a log10-uniform distribution for all possible cell states with respect to the steady state concentrations, flows and nl-parameters, it does not necessarily imply that the obtained rate coefficients follow the same distribution or lie within a specific interval.

In the following the chosen sampling intervals are discussed briefly. Since we are interested in relative changes of the oscillatory properties for relative perturbations of the parameters, the results are independent of the choice of the sampling interval. This can be shown numerically by setting the sampling region to (10^−1^, 10^5^) for all quantities. In that case the sensitivity statistics remain unaltered (S12 Fig), even though the obtained amplitudes of the intermediates change.

Moreover, we assumed that the sampling intervals for concentrations and nl-parameters are equal. Generally, nl-parameters characterize the affinity of an enzyme or channel for its substrate or regulatory intermediate. Sampling the affinity from the same interval as the intermediate concentrations allows covering all possible regulatory modes, e.g. values close to 0 and close to 1 for Michaelis-Menten or Hill terms. As an example, we show that the median value of a sampled Michaelis-Menten term is 0.5 (S13 Fig), implying that high and low values of those terms are represented equally well in the sampling process.

We found the bottom-up approach described so far advantageous over a top-down approach in which all kinetic parameters are directly randomly sampled (e.g. as applied in [24]). A top-down approach requires either simulating the ODE system for all sampled parameter sets or solving a set of non-linear equations to determine the steady state which leads to problems in cases of multi-stationarity. Our approach circumvents this issue by directly sampling the steady state concentrations. We compared the number of parameter sets for which the ODE system had to be simulated and the sensitivity results obtained for the proposed bottom-up sampling with those for a top-down sampling without previous examination of the steady state stability (Table A in the S1 File, S14 Fig). Our bottom-up approach requires simulating considerably less frequently (Table A in the S1 File) and thus yields lower computational costs than the top-down sampling approach. The obtained sensitivity results are similar for both sampling methods (S14 Fig).

### Determining steady state stability (Fig 9, box 3)

For each parameter set sampled as described in Parameter sampling we calculated the Jacobian matrix and computed its eigenvalues to assess the stability of the sampled steady state. It is stable if all eigenvalues have negative real parts, if there is at least one eigenvalue with a positive real part the steady state is unstable. We only continued the analysis for parameter sets yielding an unstable steady state.

### Numerical integration of the models (Fig 9, box 4)

For the numerical integration, starting values *S(0)* close to the steady state vector (*S*^*0*^) were used, specifically *S(0) = 0*.*95·S*^*0*^. Exceptions were models which include conserved moieties (model [49]) in which the conservation conditions had to be taken into account.

For the simulations we used MATLAB [Release 2010b, The MathWorks, Inc., Natick, Massachusetts, United States] and therein the integration methods for ordinary differential equations *ode45* (general solver) and *ode15s* (designated for stiff systems). For each parameter set the model integration was performed for 2 seconds using both methods. The method which delivered the solution to a longer integration interval was selected for further computation. The absolute and relative integration error tolerances were set to 10^−8^ and 10^−6^, respectively. The integration was performed until regular oscillations were detected (see next paragraph). Otherwise, integration was terminated if either 20 000 time units or 3 minutes (for systems with ≤10 variables) or 5 minutes (for systems with >10 variables) of calculation were reached without detecting regular oscillations. In the latter case the parameter set was discarded.

### Detection of regular oscillations and determination of period and amplitude (Fig 9, box 5)

We analyzed the numerical solution with respect to occurring maxima and minima. If the intermediate with largest difference between maximum and minimum had five consecutive equal maxima (relative precision of 10^−6^) we studied the time intervals between them. If they were equal (relative precision of 10^−4^) we classified a solution as regular oscillation and amplitude and period were calculated as described below. The system was integrated again with the end-point of the previous calculation as a starting point, using 100-fold reduced calculation tolerances until 100-fold of the calculated period was reached. The period and amplitudes of this more precise solution were determined. If the oscillatory properties of initial and precise simulations agreed, the parameter set was used for sensitivity analysis, if not, it was discarded.

The period *T* is defined as the time interval between two maxima with identical values. The amplitude *A*_*i*_ of an intermediate is the concentration difference between the largest and the lowest concentration value of the species during one period. Since each of the analyzed oscillatory models consisted of more than one species, we examined the arithmetic mean value of the amplitudes *A*_*i*_ of all species of a model as amplitude *A*.

### Sensitivity analysis and data visualization (Fig 9, boxes 6–8)

In the sensitivity analysis we are interested in the changes in period (*ΔT = T*_*perturbed*_ *- T*_*unperturbed*_) and amplitude (*ΔA = A*_*perturbed*_ *- A*_*unperturbed*_) due to changes in relevant kinetic parameters (*Δpar = par*_*perturbed*_ *- par*_*unperturbed*_). Therefore, for each sampled parameter set the rate coefficient and nl-parameters, which are maximal reaction velocities, inhibition or activation constants and Michaelis-Menten constants, were individually increased by 2%. The model with the parameter set including one perturbed parameter was again integrated and period and amplitude were determined as described above. This allowed for computing the sensitivity coefficients according to Eqs (3) and (4) (Fig 9, box 7).

The overall sensitivities per sampled parameter set were calculated from the sensitivity coefficients *R*^*A*^ and *R*^*T*^ according to Eqs (1) and (2). It was sampled until the sensitivity values for 2 500 parameter sets could be calculated. The number of parameter sets required to be sampled for that analysis is indicated for each model in the S1 File.

The period and amplitude sensitivities are depicted in scatter plots, with each dot representing the sensitivities of one parameter set (Fig 9, box 8). The median values of the corresponding sensitivity distributions are given (white circle). Moreover, the characteristics of the sensitivity distributions are captured in box-plots giving the median (line as central value), the 95% confidence interval of the median (notch, [68]), the first and third quartiles (box), the 5th and 95th percentiles (end of whiskers). In addition, the utmost 10% of the data are given as crosses, either according to their actual values if they are between the end of the whiskers and the broken line, or as arbitrary values if beyond that line. The distance between the 5th and 95th percentiles is called 90% (data) range and is used as the measure of variability.

Comparing the results of the sensitivity analysis for 2 500 to 75 000 parameter sets for two models reveals almost identical characteristics with respect to median, interquartile range, and 90% data range (S1A Fig, Table N in the S1 File). Moreover, we tracked the sensitivity characteristics for increasing sample size and found decreasing variabilities of median values and 90% data range limits (S1B Fig). This illustrates that 2 500 parameter sets are sufficient to get adequately precise information on the statistical characteristics.

In addition, we checked whether the magnitude of the parameter perturbations had an impact on the sensitivities. We obtained similar results for other small parameter perturbations (1%, 5%, 10% and -2%, S15 Fig).

The described work-flow was applied to all examined models.

### Comparison of sensitivity distributions

For all comparisons of sensitivity distributions between different models the Mann-Whitney-U test was used.

### Linear stability analysis of the chain models in Fig 2C

For the analysis of steady state stability of the chain models we examined the eigenvalues of the Jacobian matrix. We sampled until 10 000 parameter sets with *S*_*4*_*/kn*_*1*_ in (10^*a*^, 10^*a*+0.1^) for each *a = -6*, *-5*.*9*,*…*, *6* were obtained for every integer Hill coefficient *n* in the interval [1,25].

## Supporting Information

S1 Fig Influence of the sample size on sensitivities. A: Sensitivities of the phenomenological calcium model (dark blue circles) and the mammalian circadian model (dark red triangles) for sampling up to 75 000 oscillating sets are depicted (black symbols as median values). Note that this required sampling of 2 100 000 and 19 200 000 parameter sets, respectively. The sensitivities are compared to those obtained for sampling up to 2 500 oscillating sets as in our proposed analysis (red and light blue dots, white dots as median values). Box-plots of the respective sensitivities obtained for sampling up to 2 500 or 75 000 parameter sets are very similar (Table N in the S1 File) indicating that we gather the essential information already with the sample size of 2 500. B: Statistical values for increasing sample size. Following our work-flow, we performed sensitivity analyses for the mammalian circadian model (first and second column) and the phenomenological calcium model (third and fourth column) with sample sizes of 25, 50, 100, 250, 500 and 1 000 sets. For each sample size, we performed 15 independent sensitivity analyses and depicted the median values (upper row), the 95th percentiles (middle row) and the 5th percentiles (lower row) versus the sample size (black dots). In addition, the according values for sample size 2 500 and 75 000 are included (symbols as in A). For all three statistical characteristics and all considered sensitivity distributions, the variance in the obtained statistical measures decreases with increasing sample size. This indicates that also the precision increases with increasing sample size.
(TIF)

S2 Fig Impact of the sensitivity measure definition on sensitivity results. The original sensitivity distributions of the mammalian circadian model [37] are depicted in red triangles, those of the phenomenological calcium oscillation model [38] in blue circles. Median values are given by black symbols. A: Sensitivities averaging the sensitivity coefficients of the rate coefficients only (one for each flow) are given by black dots, white symbols for the corresponding median values. B: Sensitivities averaging the three largest absolute sensitivity coefficients among rate coefficients and nl-parameters are given by dark red triangles (circadian model) or dark blue circles (calcium model). White symbols indicate the corresponding median values. For altered sensitivity measures, the sensitivities of both models also segregate into different populations with similar characteristics as for the overall sensitivity employed in the manuscript.
(TIF)

S3 Fig Impact of the Hill coefficient on the sensitivities of the chain models. Sensitivities of the chain models with negative feedback (orange stars, median values given by the black star) and with positive feedback and Hill coefficient *n =* 2 (dark green squares, median values given by the black square) are compared to the sensitivities of the positive feedback chain model with Hill coefficient *n =* 9 (dark dots, median values given by the white square). Thus, overall tendencies of the sensitivities are kept for the comparison of the negative and positive feedback chain model if altering the Hill coefficient.
(TIF)

S4 Fig Bifurcation diagrams for the chain model with negative feedback. For each of the parameter sets marked by a black star in panel D, a bifurcation analysis (with XPPAUT [69]) is performed using the parameter with largest absolute period sensitivity coefficient as bifurcation parameter. Given are plots of species S_1_ and the period versus the bifurcation parameter. Small panels (right hand side) show details for specific intervals of the bifurcation parameter. Red lines denote stable steady states, dotted black lines unstable steady states. Green dots (often melting to a line) denote stable limit cycles which arise from Hopf bifurcations in A-C. Blue circles denote unstable limit cycles. The dotted gray vertical lines indicate the original value of the bifurcation parameter and its perturbation (+2%) for the parameter set examined. Note that the examined parameter sets leading to sensitivities in B and C occur rarely for the model as they lie outside the 90% data range (end of whiskers, indicated by dashed lines in D).
Overall, the periods vary only slightly thus resulting in low period sensitivities (middle panels). The amplitudes vary smoothly between the bifurcations. In panel B, the change in the amplitude is more pronounced if the bifurcation parameter yields values close to the bifurcations. For the parameter set in B, this results in a large amplitude sensitivity. Overall, the amplitude sensitivity obtained for a particular parameter set is dependent on the distance of the parameter values to the according bifurcations.
(TIF)

S5 Fig Bifurcation diagrams for the chain model with positive feedback. For each of the parameter sets marked by a black square in panel E, a bifurcation analysis (with XPPAUT [69]) is performed using the parameter with largest absolute period sensitivity coefficient as bifurcation parameter. Given are plots of species S_1_ and the period versus the bifurcation parameter. Small panels (right hand side) show details for specific ranges of the bifurcation parameter. Red lines denote stable steady states, dotted black lines unstable steady states. Green dots (often melting to a line) denote stable limit cycles, blue circles unstable limit cycles. The dotted gray vertical lines indicate the original value of the bifurcation parameter and its perturbation (+2%) for the parameter set examined. Note that the examined parameter sets leading to sensitivities in B-D occur rarely for the model as they lie outside the 90% data range (end of whiskers, indicated by dashed lines in E).
In general, the periods change strongly for bifurcation parameter values close to bifurcations (e.g. for parameter sets B-D) but less for intermediate bifurcation parameter values (parameter set A). This results in high, low or intermediate period sensitivities depending on the distance of the parameter values to according bifurcations. The amplitudes vary strongly in a small range of the bifurcation parameter (mainly close to bifurcations, except for parameter set D) and remain nearly constant for a large range of the bifurcation parameter (further away from bifurcations). Therefore, in the positive feedback chain model, many parameter sets display rather low amplitude sensitivities. Bifurcation parameter variation can lead to transitions between limit cycles with different periods and/or amplitudes (panels A, B, C). This results in very high period and amplitude sensitivities for the specific parameter sets. Also regions of birhythmicity can be found (see panels A, B).
(TIF)

S6 Fig Impact of reaction kinetics on the sensitivity coefficients. Sensitivity coefficients of the models with Michaelis-Menten kinetics in all degradation and conversion reactions compared to the models with mass action kinetics for negative feedback (panels A, B) or positive feedback (panels C, D). The absolute period sensitivity coefficient |R^T^| distributions (panels A, C) or absolute amplitude sensitivity |R^A^| distributions (panels B, D) are shown as box-plots. In each panel, the results for the rate coefficient k_i_ of the reaction for the model with mass action kinetics (medians given in orange, light green), for the maximal reaction velocity V_i_ and, if applicable, for the K_M_-value of the reaction K_i_ for the model with Michaelis-Menten kinetics (medians given in black) are given next to each other to allow for direct comparison.
The increase in the sensitivities in the models with Michaelis-Menten kinetics does not solely result from the introduction of the K_M_-values but also from an increase in the sensitivities of the rate coefficients.
(TIF)

S7 Fig Impact of mass conservation properties on the sensitivity coefficients. Distributions of absolute period sensitivity coefficients |R^T^| (A, C) and absolute amplitude sensitivity coefficients |R^A^| (B, D) for the rate coefficients and nl-parameter of the chain model with negative feedback (A, B) or with positive feedback (C, D). The distributions are given for the models with reactions 2, 4 and 6 being conversions (A-D, left panels) or for the models with all three of these reactions being regulated productions for the negative feedback chain model (A, B, right panels) or with reactions 4 and 6 being regulated productions for the positive feedback chain model (C, D, right panels).
For the negative feedback chain model, substituting conversions by regulated productions leads to a shift in sensitivities among the parameters: In the model with regulated productions, the influence of rate coefficients 4 and 6 is redistributed to rate coefficients 5 and 7. This implies that alterations in the degradation part of a reaction are affecting the period and amplitude more than alterations in the production part. The less symmetrical distribution of sensitivity values among the parameters leads to a slight increase in the overall sensitivities which are composed as quadratic mean of the sensitivity coefficients.
For the positive feedback chain model, a strong increase in sensitivity coefficients of parameters 4–7 are observed for the model with regulated productions compared to the model with reactions 4 and 6 being conversions. The reason is the decoupling of production and degradation processes in the model with regulated productions. The period and amplitude are more sensitive to changes in one individual species as in degradations or regulated production, whereas perturbations in conversions which combine the decrease of the preceding species and the increase of the subsequent species can be compensated.
(TIF)

S8 Fig Summary of the sensitivities obtained for the chain models. A: Sensitivities of the chain models with negative feedback (N1-N4) or positive feedback (P1-P4) with mass conversions only and mass action kinetics (N1, P1), with mass conversions only and Michaelis-Menten kinetics in all reactions (N2, P2), with regulated productions and mass action kinetics (N3, P3) or with regulated productions and Michaelis-Menten kinetics in all reactions (N4, P4). B: Box-plots of the period and amplitude sensitivity distributions from A.
The introduction of reactions with Michaelis-Menten kinetics leads to increased median period as well as amplitude sensitivities irrespective of the type of feedback and the mass conservation properties of the chain model (compare N1 to N2, N3 to N4, P1 to P2 and P3 to P4). In the negative feedback chain model, the impact of altering the mass conservation properties is dependent on the implemented kinetics. In particular, for Michaelis-Menten kinetics, the model with regulated productions (N4) exhibits slightly lower sensitivities than the model including conversion reactions (N2) while the effect is a slight increase for linear kinetics (compare N1 to N3, or see Fig 4D). However, the impact of altering the mass conservation properties remains moderate irrespective of the applied kinetics (compare N1 to N3, N2 to N4, P1 to P3, P2 to P4).
(TIF)

S9 Fig Model structures of the models examined in Fig 7. The column ‘Id’ gives the identifier of the model according to Fig 7. In the model structure column, black and gray arrows denote reactions in the models, reactions with Michaelis-Menten kinetics (MM) are thereby marked in gray. Red lines ending in T-shape indicate negative regulations, green arrows denote positive regulations.
(TIF)

S10 Fig Shape of the oscillations and sensitivities for the FitzHugh-Nagumo model and a λ-ω system. A, B: Depicted are the solutions to the model equations (see S1 Supplementary Information) following the limit cycle at the reference parameter sets of the FitzHugh-Nagumo model for neural dynamics [60] (A) and of the chosen λ-ω system (sometimes referred to as A-B system) as canonical Hopf bifurcation oscillator [61] (B). C, D: Sensitivities of the FitzHugh-Nagumo model (C) and the λ-ω oscillator (D). For comparison, the box-plots of the sensitivities of the chain models with mass action kinetics and negative feedback (orange, neg) or positive feedback (green, pos) are shown.
The FitzHugh-Nagumo model and the λ-ω oscillator are not based on descriptions of biochemical reactions, but are of phenomenological nature evoking oscillatory curves of different shapes that can even take negative values. While the FitzHugh-Nagumo model delivers relaxation oscillation curves, the λ-ω system evokes regular, sine-oscillation curves (A, B and S1 Supplementary Information).
We compared their sensitivities to those of the chain models with mass action kinetics. The FitzHugh-Nagumo model has higher period sensitivities and lower amplitude sensitivities than both the negative feedback chain model and the positive feedback chain model (4.1- and 1.15-fold increased median period sensitivity, respectively, 1.2- and 1.04-fold reduced median amplitude sensitivity, respectively, p-values <10^−5^, Tables R, S in the S1 File). Thus, the FitzHugh-Nagumo model displays rather low amplitude sensitivities and high period sensitivities.
The implementation of the λ-ω oscillator shown here has period sensitivities which are higher than those of the negative feedback chain model and lower than those of the positive feedback chain model (3.3-fold increased and 1.1-fold reduced median period sensitivity, respectively, p-values <10^−5^, Tables R, S in the S1 File). The amplitude sensitivities are lower than for both chain models (1.5- and 1.3-fold reduced median amplitude sensitivity, respectively, p-values are 0, Tables R, S in the S1 File). Hence, the λ-ω oscillator shows intermediate, variable period sensitivities and low amplitude sensitivities.
(TIF)

S11 Fig Illustration of the sampling of the steady state flow vector *ν*^*0*^ *= ν*. Basis is the condition *N·ν = 0* for *ν* being the flow vector at steady state for the stoichiometric *m×M*-matrix *N = (η*_*ij*_*)* which is formed by the stoichiometric coefficients. The order in which the flows are set has to be chosen randomly in order to avoid bias upon the sampling results. This procedure is explained in the decision diagram. To accelerate the sampling process, independent parts of the stoichiometric matrix *N* and according sets of flows can be sampled separately.
(TIF)

S12 Fig Influence of the choice of the sampling interval on the results of the sensitivity analysis. Examined is the phenomenological calcium oscillation model [38]. Blue circles, median values given by black circles: original sampling procedure in the interval (10^−3^, 10^3^), black dots, median values given by white diamonds: results when sampling in the interval (10^−1^, 10^5^). A: Period *T* versus period sensitivity *σ*_*T*_. B: Amplitude *A* versus amplitude sensitivity *σ*_*A*_. C: Period sensitivity *σ*_*T*_ versus amplitude sensitivity *σ*_*A*_. Altering the sampling interval results in only minor alterations of the obtained periods (A) but increased amplitudes (B). The sensitivities are not influenced by the sampling interval, since they are derived from relative changes of period and amplitude (C, Tables T, U in the S1 File).
(TIF)

S13 Fig Values obtained by the sampling method when sampling 10^5^ parameter sets. A: Histogram for a sampled steady state species concentration *S*^*0*^. B: Histogram for a sampled *K*_*M*_-value. C: Calculated value of the ratio between steady state concentration *S*^*0*^ and *K*_*M*_-value. D: Calculated Michaelis-Menten term *S*^*0*^*/(S*^*0*^ *+ K*_*M*_*)* and the estimated cumulative probability function (solid line). A cumulative probability of 0.5 is indicated by the dashed line.
The sampling ensures a log10-uniform distribution for the steady state concentrations and the nl-parameters. By choosing the same sampling interval and sampling distribution for these quantities, their ratios are symmetrically distributed with a central value of 1 (see C). The resulting Michaelis-Menten terms take values between 0 and 1. They are in 50% of the sampling cases lower and in 50% higher than 0.5 (see D).
(TIF)

S14 Fig The impact of the sampling procedure on the sensitivity results. Given are the sensitivities of the mammalian circadian model [37] (A), the phenomenological calcium model [38] (B), the negative feedback chain model with linear kinetics (C) and the positive feedback chain model with linear kinetics (D) for the sampling procedure as proposed in the manuscript (colored symbols, median values are given by colored symbols with white frame) compared to the sensitivities of 1 000 parameter sets sampled using a top-down sampling approach (black dots, median values given by white circles). The top-down approach includes (i) sampling the nl-parameters, rate coefficients and initial concentrations log10-uniformly in the interval (10^−3^, 10^3^), (ii) performing simulations starting from the sampled initial conditions, (iii) checking for oscillations, and (iv) perturbing the individual parameters followed by simulations to estimate the sensitivity coefficients. The box-plots show the distribution characteristics of the period sensitivities (top) and amplitude sensitivities (right) for 1 000 parameter sets.
As can be seen in panels A-D the distribution characteristics are similar for both sampling approaches. The regions occupied in sensitivity space, these are the regions filled by the scatter plots, are highly consistent between the two approaches. The only slight deviation between the approaches is found for the median values in panel B. Since the sampling approach proposed here makes use of stability properties of the steady states, it requires much less effort concerning numerical integration (compare Table A in the S1 File) and is thus the more efficient method.
(TIF)

S15 Fig Impact of the parameter perturbation value on the sensitivity results. A: Results of the sensitivity analysis for the model of mammalian circadian oscillations and the phenomenological calcium model with a parameter perturbation *Δp =* 2% (blue: calcium model, red: circadian model, median values given by black symbols) or *Δp =* 10% (black dots, median values given by white diamonds). B and C: Period and amplitude sensitivity distributions, respectively, as box-plots for different parameter perturbation values (*Δp =* 1%, 2%, 5%, 10%). The according distribution for *Δp =* 2% is depicted in color (red: circadian model, blue: calcium model). D: Results of the sensitivity analysis for both models with *Δp =* 2% (red: circadian model, blue: calcium model, median values given by black symbols) or *Δp =* −2% (black dots, median values given by white diamonds). E and F: Period and amplitude sensitivity distributions, respectively, from panel D as box-plots. For all analyses, we compared sensitivity values for 250 parameter sets.
Altering the parameter perturbation does not induce significant changes concerning the calcium model or the amplitude sensitivities of the circadian model (with a confidence level of 0.01, p-values of the Mann-Whitney-U test >0.035, Table W in the S1 File). The median period sensitivity of the circadian rhythm model is only slightly altered by 2.9% for *Δp =* -2%, and it is affected for *Δp =* 10%. However, no substantial gain in information is obtained for different parameter perturbations therefore only perturbations of *Δp =* 2% are applied.
(TIF)

S1 File Supplementary Tables. The characteristics for the sensitivity distributions and the detailed statistics of the Mann-Whitney-U tests are given.
(PDF)

S1 Supplementary Information Model descriptions and equations. The descriptions and equations for the three circadian models, the three calcium models, the chain models, the repressilator, the models for MAPK, glycolysis and the cell cycle, as well as the FitzHugh-Nagumo model and the λ-ω system are given.
(PDF)
