# Supplementary figures and images for: Feedback, Mass Conservation and Reaction Kinetics Impact the Robustness of Cellular Oscillations
Source: PLoS Comput Biol. 2016 Dec 27;12(12):e1005298. doi: 10.1371/journal.pcbi.1005298 (PMC5226835; doi:10.1371/journal.pcbi.1005298)

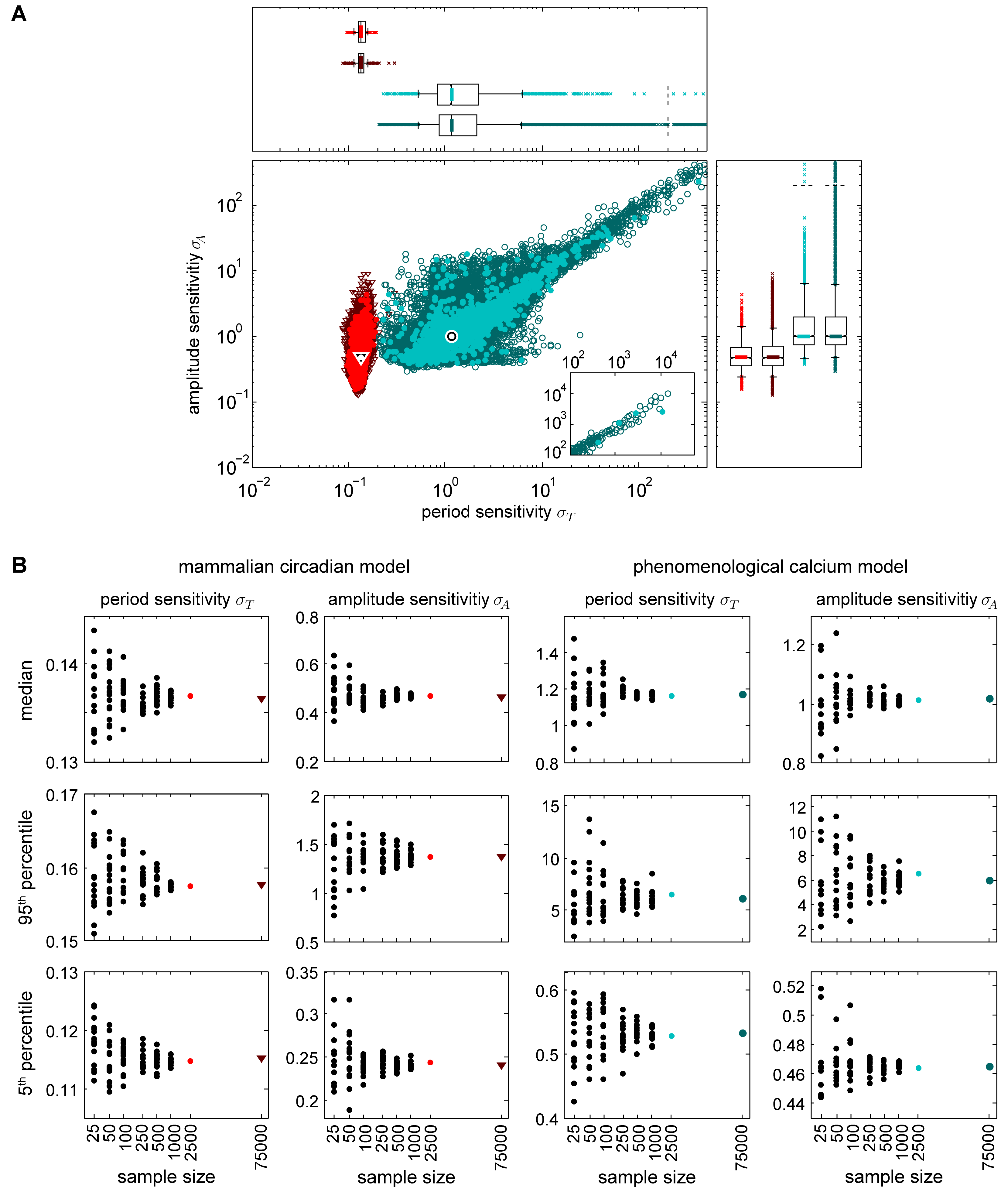

Supplement: S1 Fig — A: Sensitivities of the phenomenological calcium model (dark blue circles) and the mammalian circadian model (dark red triangles) for sampling up to 75 000 oscillating sets are depicted (black symbols as median values). Note that this required sampling of 2 100 000 and 19 200 000 parameter sets, respectively. The sensitivities are compared to those obtained for sampling up to 2 500 oscillating sets as in our proposed analysis (red and light blue dots, white dots as median values). Box-plots of the respective sensitivities obtained for sampling up to 2 500 or 75 000 parameter sets are very similar (Table N in the S1 File) indicating that we gather the essential information already with the sample size of 2 500. B: Statistical values for increasing sample size. Following our work-flow, we performed sensitivity analyses for the mammalian circadian model (first and second column) and the phenomenological calcium model (third and fourth column) with sample sizes of 25, 50, 100, 250, 500 and 1 000 sets. For each sample size, we performed 15 independent sensitivity analyses and depicted the median values (upper row), the 95th percentiles (middle row) and the 5th percentiles (lower row) versus the sample size (black dots). In addition, the according values for sample size 2 500 and 75 000 are included (symbols as in A). For all three statistical characteristics and all considered sensitivity distributions, the variance in the obtained statistical measures decreases with increasing sample size. This indicates that also the precision increases with increasing sample size. (TIF) [file pcbi.1005298.s001.tif]

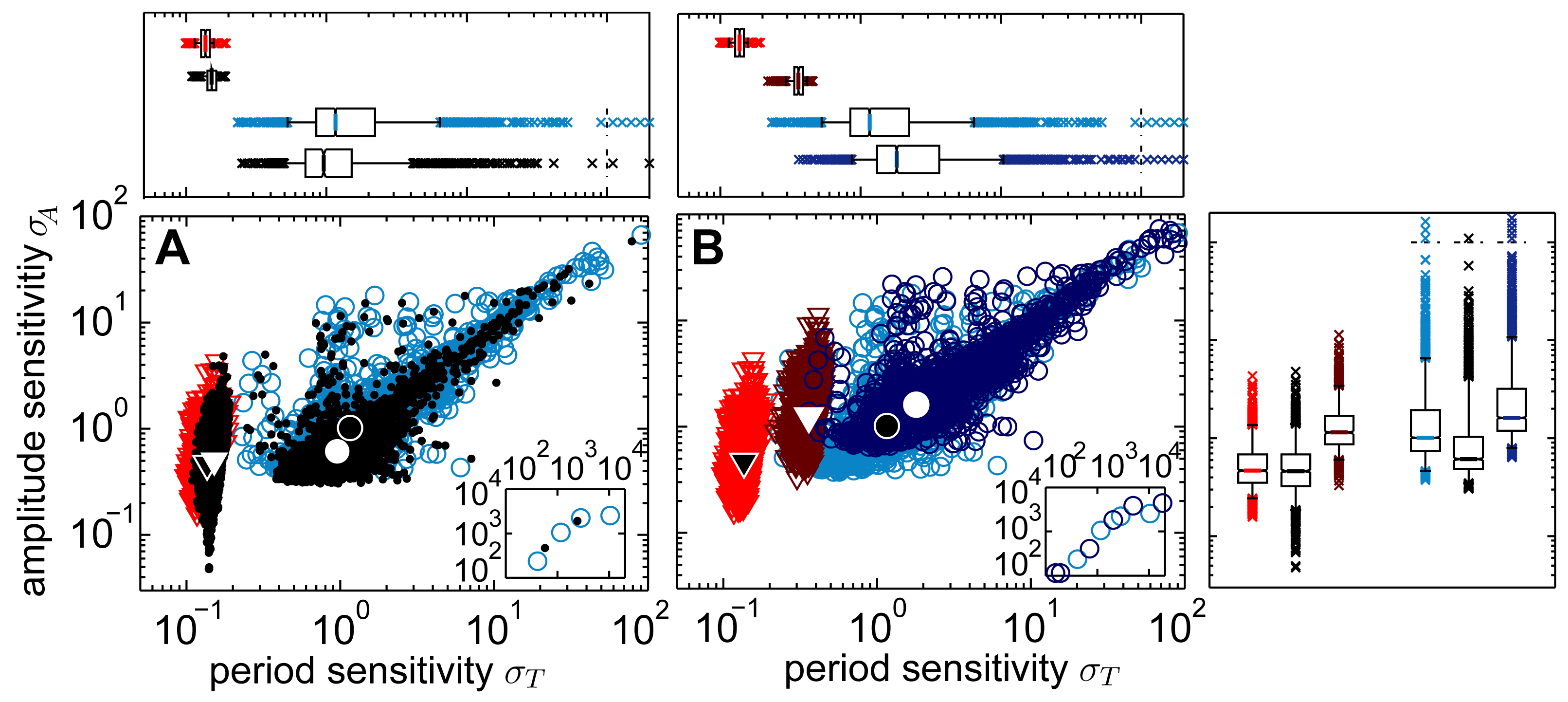

Supplement: S2 Fig — The original sensitivity distributions of the mammalian circadian model [37] are depicted in red triangles, those of the phenomenological calcium oscillation model [38] in blue circles. Median values are given by black symbols. A: Sensitivities averaging the sensitivity coefficients of the rate coefficients only (one for each flow) are given by black dots, white symbols for the corresponding median values. B: Sensitivities averaging the three largest absolute sensitivity coefficients among rate coefficients and nl-parameters are given by dark red triangles (circadian model) or dark blue circles (calcium model). White symbols indicate the corresponding median values. For altered sensitivity measures, the sensitivities of both models also segregate into different populations with similar characteristics as for the overall sensitivity employed in the manuscript. (TIF) [file pcbi.1005298.s002.tif]

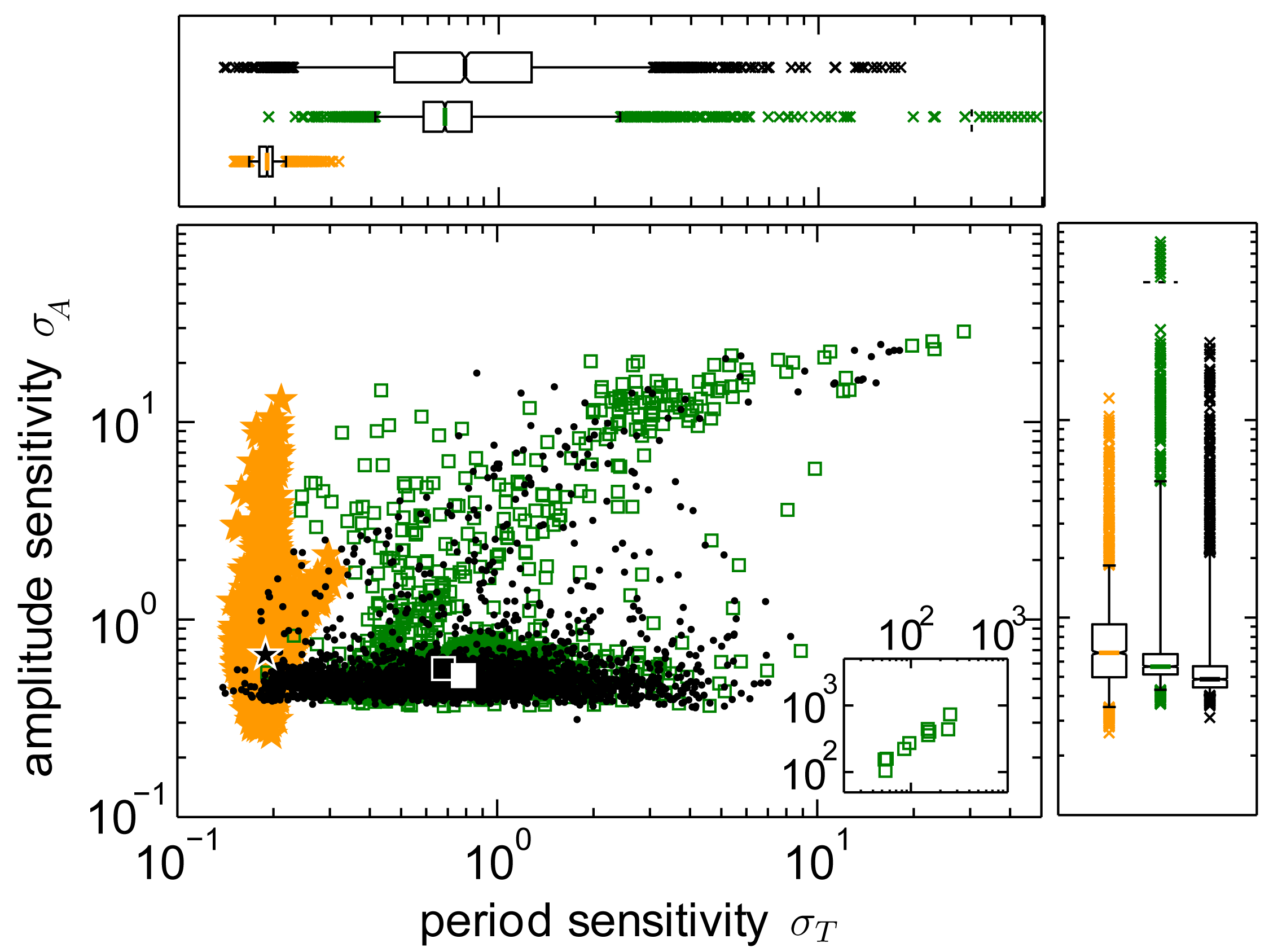

Supplement: S3 Fig — Sensitivities of the chain models with negative feedback (orange stars, median values given by the black star) and with positive feedback and Hill coefficient n = 2 (dark green squares, median values given by the black square) are compared to the sensitivities of the positive feedback chain model with Hill coefficient n = 9 (dark dots, median values given by the white square). Thus, overall tendencies of the sensitivities are kept for the comparison of the negative and positive feedback chain model if altering the Hill coefficient. (TIF) [file pcbi.1005298.s003.tif]

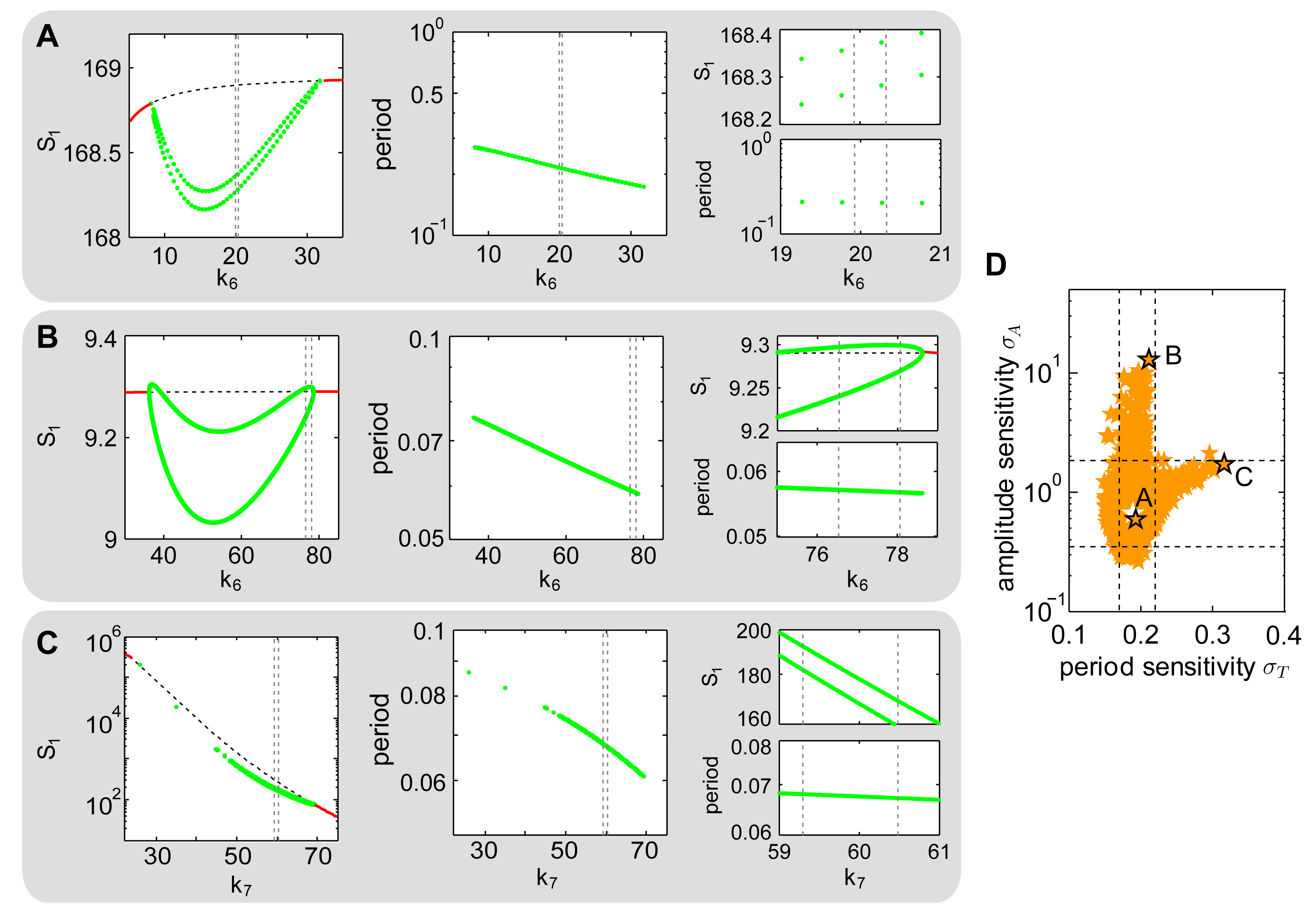

Supplement: S4 Fig — For each of the parameter sets marked by a black star in panel D, a bifurcation analysis (with XPPAUT [69]) is performed using the parameter with largest absolute period sensitivity coefficient as bifurcation parameter. Given are plots of species S1 and the period versus the bifurcation parameter. Small panels (right hand side) show details for specific intervals of the bifurcation parameter. Red lines denote stable steady states, dotted black lines unstable steady states. Green dots (often melting to a line) denote stable limit cycles which arise from Hopf bifurcations in A-C. Blue circles denote unstable limit cycles. The dotted gray vertical lines indicate the original value of the bifurcation parameter and its perturbation (+2%) for the parameter set examined. Note that the examined parameter sets leading to sensitivities in B and C occur rarely for the model as they lie outside the 90% data range (end of whiskers, indicated by dashed lines in D). Overall, the periods vary only slightly thus resulting in low period sensitivities (middle panels). The amplitudes vary smoothly between the bifurcations. In panel B, the change in the amplitude is more pronounced if the bifurcation parameter yields values close to the bifurcations. For the parameter set in B, this results in a large amplitude sensitivity. Overall, the amplitude sensitivity obtained for a particular parameter set is dependent on the distance of the parameter values to the according bifurcations. (TIF) [file pcbi.1005298.s004.tif]

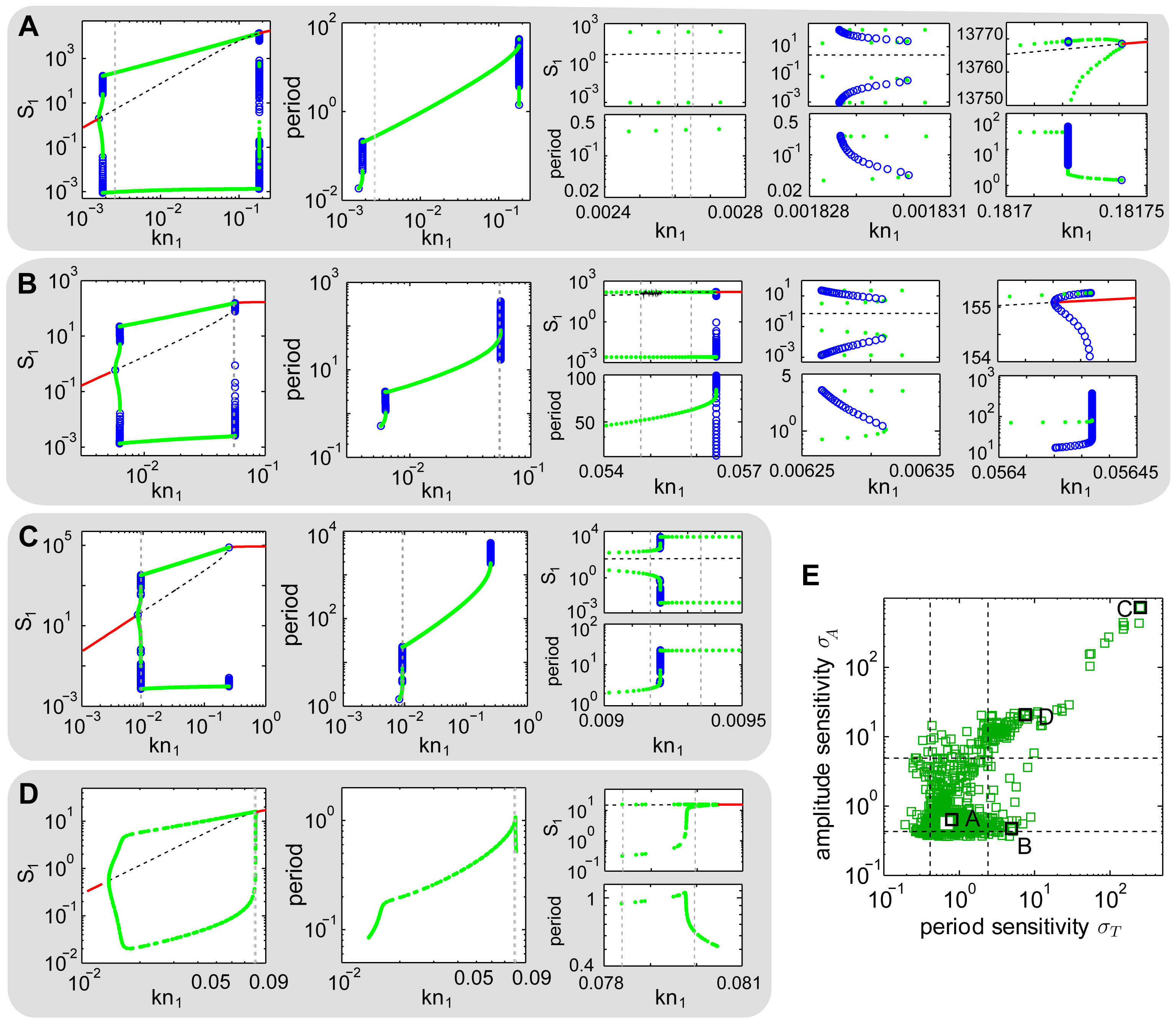

Supplement: S5 Fig — For each of the parameter sets marked by a black square in panel E, a bifurcation analysis (with XPPAUT [69]) is performed using the parameter with largest absolute period sensitivity coefficient as bifurcation parameter. Given are plots of species S1 and the period versus the bifurcation parameter. Small panels (right hand side) show details for specific ranges of the bifurcation parameter. Red lines denote stable steady states, dotted black lines unstable steady states. Green dots (often melting to a line) denote stable limit cycles, blue circles unstable limit cycles. The dotted gray vertical lines indicate the original value of the bifurcation parameter and its perturbation (+2%) for the parameter set examined. Note that the examined parameter sets leading to sensitivities in B-D occur rarely for the model as they lie outside the 90% data range (end of whiskers, indicated by dashed lines in E). In general, the periods change strongly for bifurcation parameter values close to bifurcations (e.g. for parameter sets B-D) but less for intermediate bifurcation parameter values (parameter set A). This results in high, low or intermediate period sensitivities depending on the distance of the parameter values to according bifurcations. The amplitudes vary strongly in a small range of the bifurcation parameter (mainly close to bifurcations, except for parameter set D) and remain nearly constant for a large range of the bifurcation parameter (further away from bifurcations). Therefore, in the positive feedback chain model, many parameter sets display rather low amplitude sensitivities. Bifurcation parameter variation can lead to transitions between limit cycles with different periods and/or amplitudes (panels A, B, C). This results in very high period and amplitude sensitivities for the specific parameter sets. Also regions of birhythmicity can be found (see panels A, B). (TIF) [file pcbi.1005298.s005.tif]

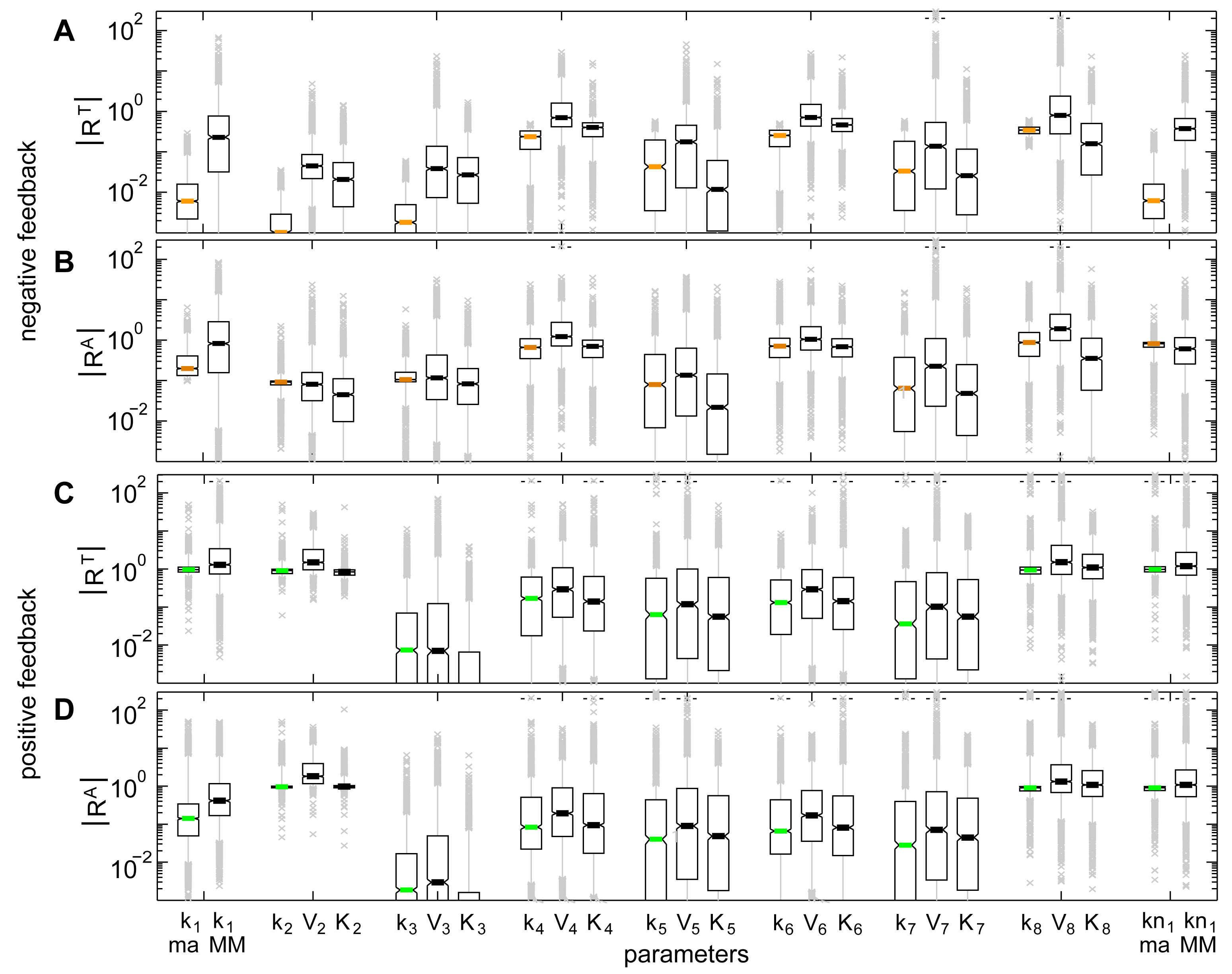

Supplement: S6 Fig — Sensitivity coefficients of the models with Michaelis-Menten kinetics in all degradation and conversion reactions compared to the models with mass action kinetics for negative feedback (panels A, B) or positive feedback (panels C, D). The absolute period sensitivity coefficient |RT| distributions (panels A, C) or absolute amplitude sensitivity |RA| distributions (panels B, D) are shown as box-plots. In each panel, the results for the rate coefficient ki of the reaction for the model with mass action kinetics (medians given in orange, light green), for the maximal reaction velocity Vi and, if applicable, for the KM-value of the reaction Ki for the model with Michaelis-Menten kinetics (medians given in black) are given next to each other to allow for direct comparison. The increase in the sensitivities in the models with Michaelis-Menten kinetics does not solely result from the introduction of the KM-values but also from an increase in the sensitivities of the rate coefficients. (TIF) [file pcbi.1005298.s006.tif]

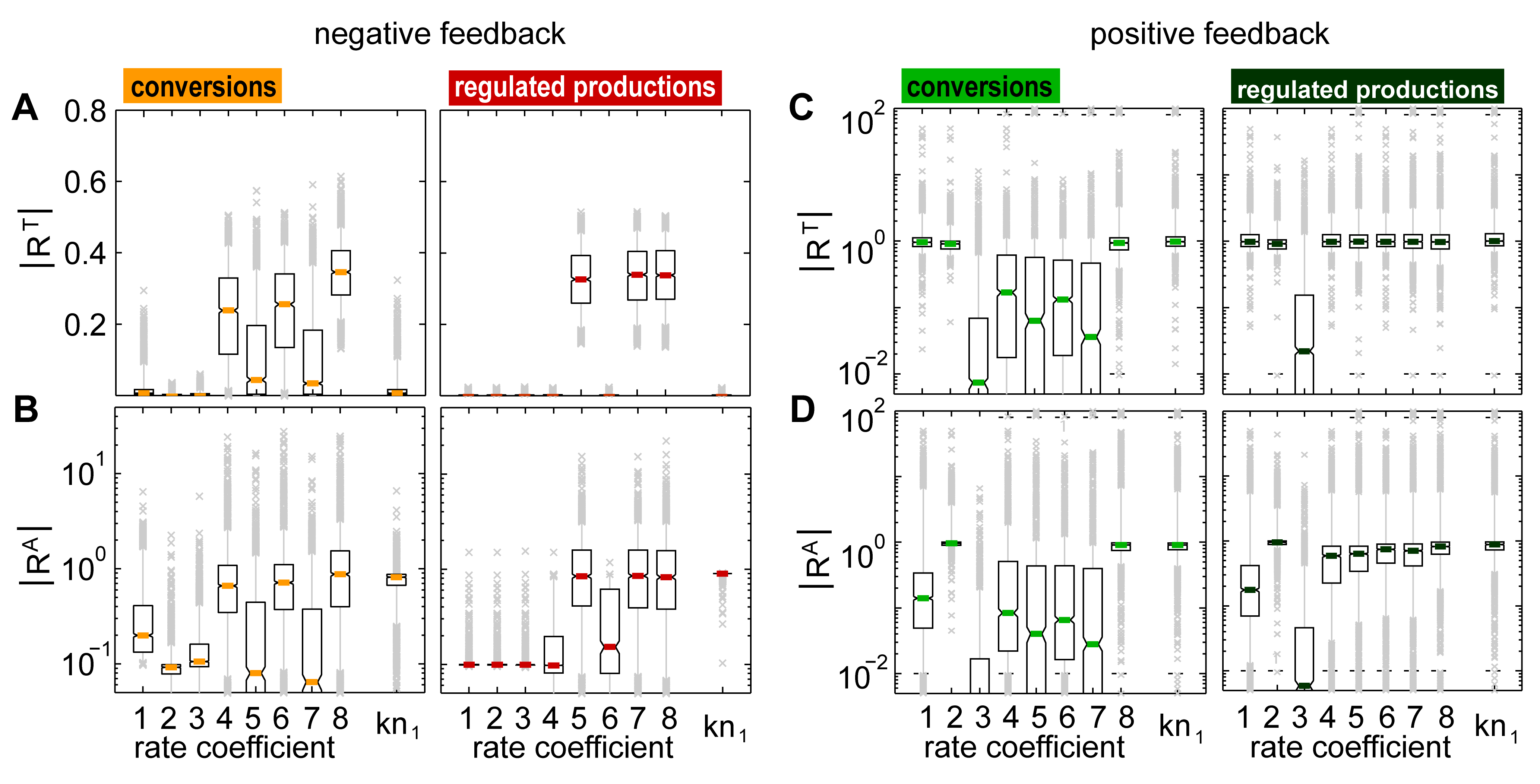

Supplement: S7 Fig — Distributions of absolute period sensitivity coefficients |RT| (A, C) and absolute amplitude sensitivity coefficients |RA| (B, D) for the rate coefficients and nl-parameter of the chain model with negative feedback (A, B) or with positive feedback (C, D). The distributions are given for the models with reactions 2, 4 and 6 being conversions (A-D, left panels) or for the models with all three of these reactions being regulated productions for the negative feedback chain model (A, B, right panels) or with reactions 4 and 6 being regulated productions for the positive feedback chain model (C, D, right panels). For the negative feedback chain model, substituting conversions by regulated productions leads to a shift in sensitivities among the parameters: In the model with regulated productions, the influence of rate coefficients 4 and 6 is redistributed to rate coefficients 5 and 7. This implies that alterations in the degradation part of a reaction are affecting the period and amplitude more than alterations in the production part. The less symmetrical distribution of sensitivity values among the parameters leads to a slight increase in the overall sensitivities which are composed as quadratic mean of the sensitivity coefficients. For the positive feedback chain model, a strong increase in sensitivity coefficients of parameters 4–7 are observed for the model with regulated productions compared to the model with reactions 4 and 6 being conversions. The reason is the decoupling of production and degradation processes in the model with regulated productions. The period and amplitude are more sensitive to changes in one individual species as in degradations or regulated production, whereas perturbations in conversions which combine the decrease of the preceding species and the increase of the subsequent species can be compensated. (TIF) [file pcbi.1005298.s007.tif]

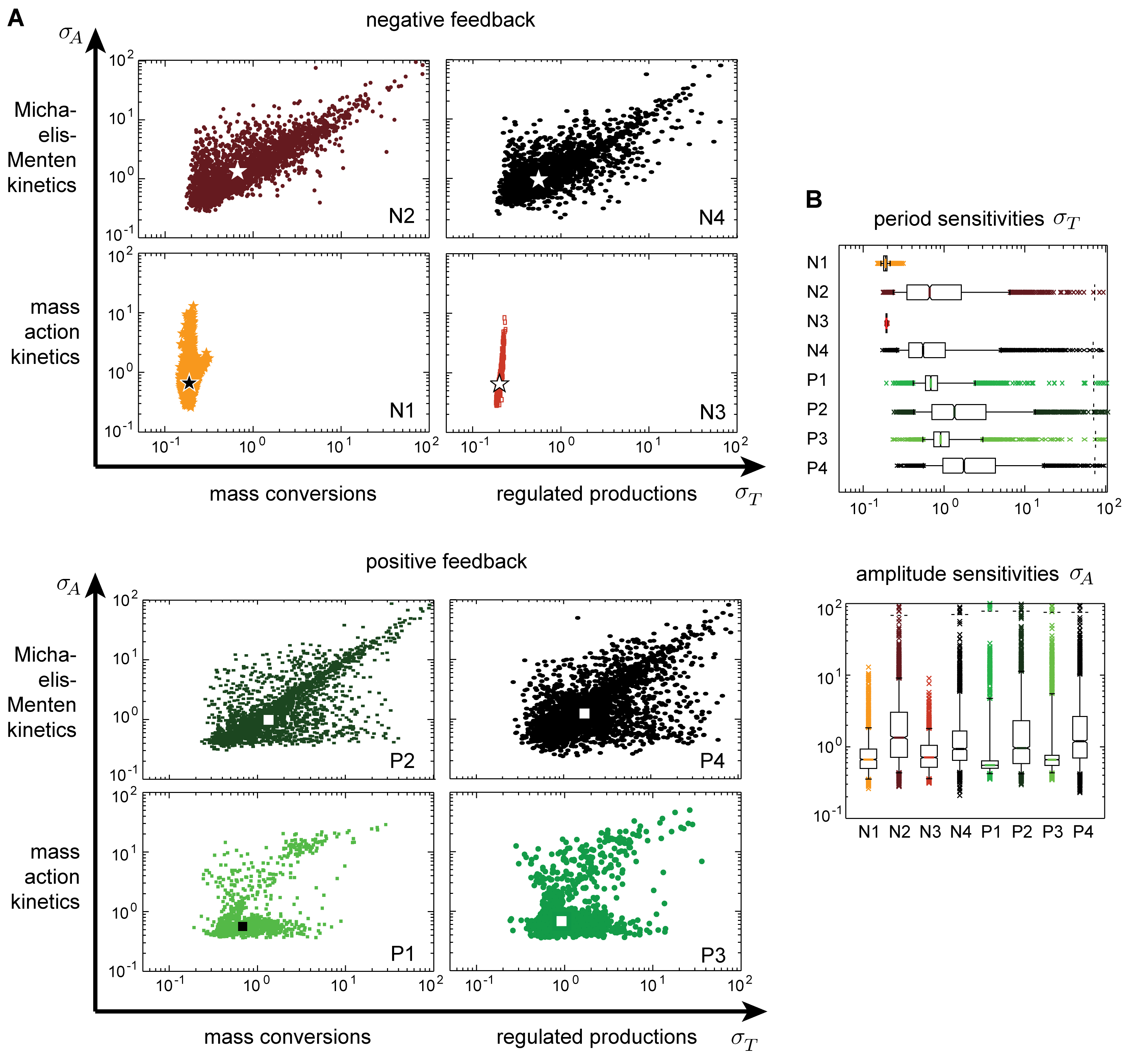

Supplement: S8 Fig — A: Sensitivities of the chain models with negative feedback (N1-N4) or positive feedback (P1-P4) with mass conversions only and mass action kinetics (N1, P1), with mass conversions only and Michaelis-Menten kinetics in all reactions (N2, P2), with regulated productions and mass action kinetics (N3, P3) or with regulated productions and Michaelis-Menten kinetics in all reactions (N4, P4). B: Box-plots of the period and amplitude sensitivity distributions from A. The introduction of reactions with Michaelis-Menten kinetics leads to increased median period as well as amplitude sensitivities irrespective of the type of feedback and the mass conservation properties of the chain model (compare N1 to N2, N3 to N4, P1 to P2 and P3 to P4). In the negative feedback chain model, the impact of altering the mass conservation properties is dependent on the implemented kinetics. In particular, for Michaelis-Menten kinetics, the model with regulated productions (N4) exhibits slightly lower sensitivities than the model including conversion reactions (N2) while the effect is a slight increase for linear kinetics (compare N1 to N3, or see Fig 4D). However, the impact of altering the mass conservation properties remains moderate irrespective of the applied kinetics (compare N1 to N3, N2 to N4, P1 to P3, P2 to P4). (TIF) [file pcbi.1005298.s008.tif]

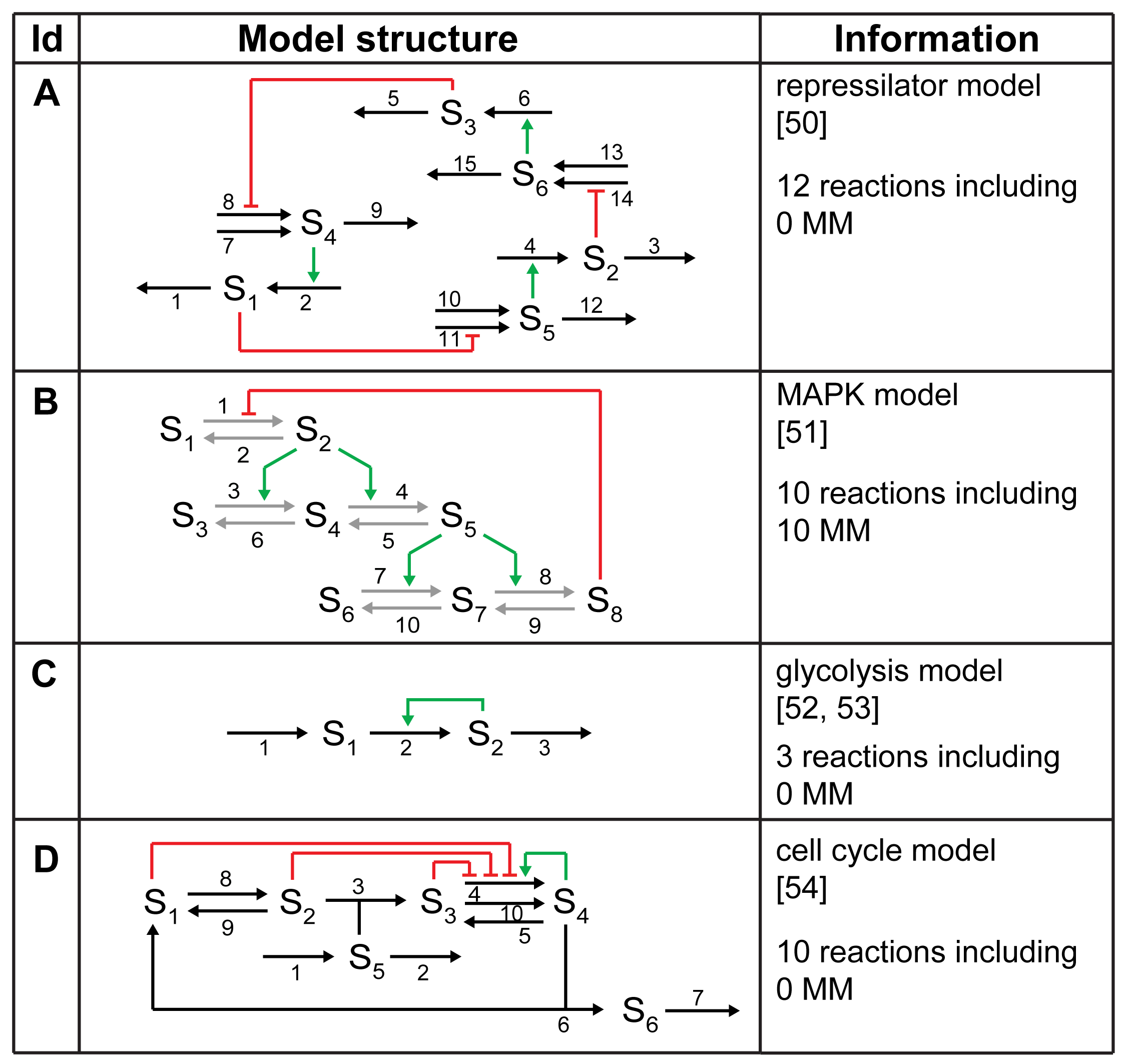

Supplement: S9 Fig — The column ‘Id’ gives the identifier of the model according to Fig 7. In the model structure column, black and gray arrows denote reactions in the models, reactions with Michaelis-Menten kinetics (MM) are thereby marked in gray. Red lines ending in T-shape indicate negative regulations, green arrows denote positive regulations. (TIF) [file pcbi.1005298.s009.tif]

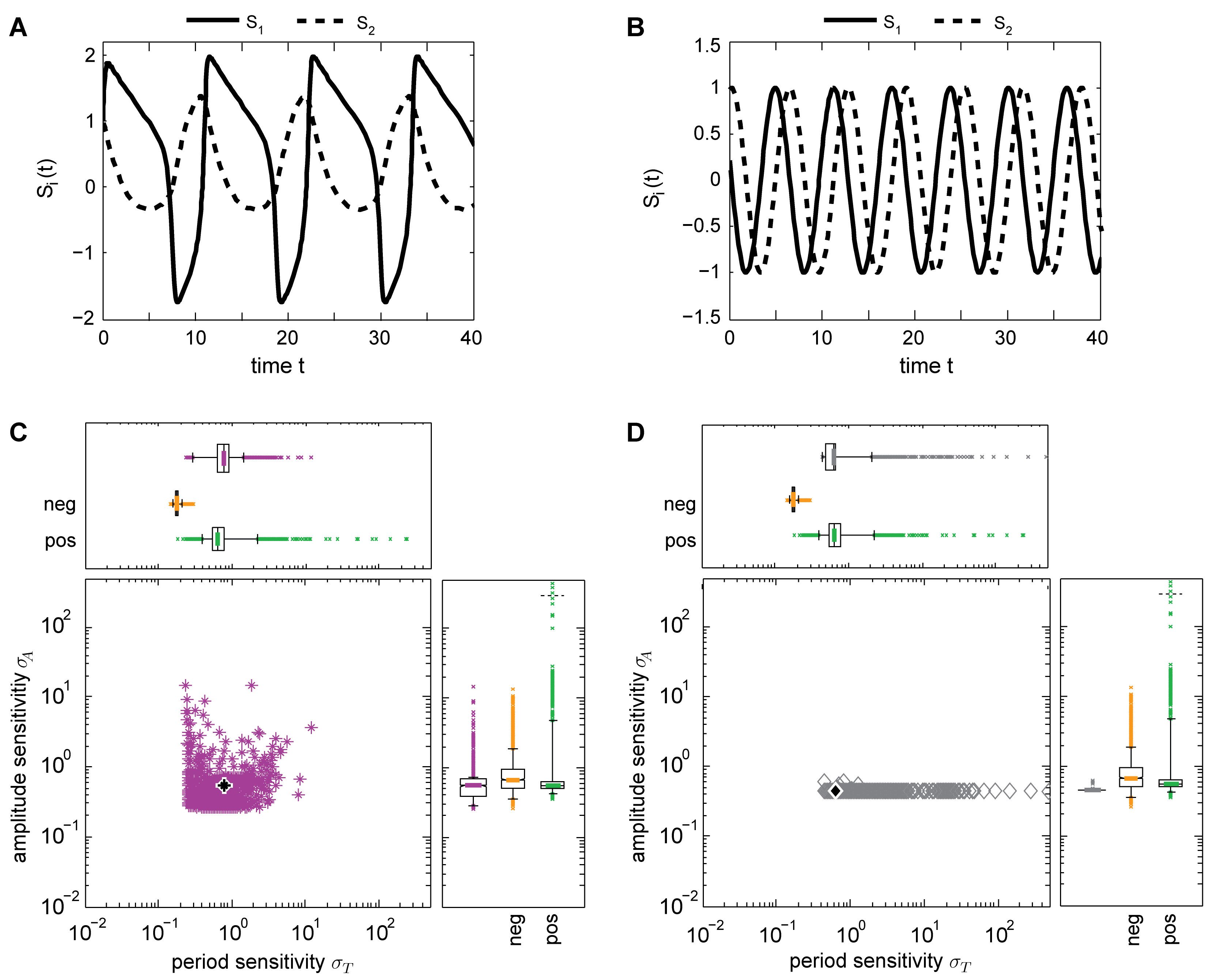

Supplement: S10 Fig — A, B: Depicted are the solutions to the model equations (see S1 Supplementary Information) following the limit cycle at the reference parameter sets of the FitzHugh-Nagumo model for neural dynamics [60] (A) and of the chosen λ-ω system (sometimes referred to as A-B system) as canonical Hopf bifurcation oscillator [61] (B). C, D: Sensitivities of the FitzHugh-Nagumo model (C) and the λ-ω oscillator (D). For comparison, the box-plots of the sensitivities of the chain models with mass action kinetics and negative feedback (orange, neg) or positive feedback (green, pos) are shown. The FitzHugh-Nagumo model and the λ-ω oscillator are not based on descriptions of biochemical reactions, but are of phenomenological nature evoking oscillatory curves of different shapes that can even take negative values. While the FitzHugh-Nagumo model delivers relaxation oscillation curves, the λ-ω system evokes regular, sine-oscillation curves (A, B and S1 Supplementary Information). We compared their sensitivities to those of the chain models with mass action kinetics. The FitzHugh-Nagumo model has higher period sensitivities and lower amplitude sensitivities than both the negative feedback chain model and the positive feedback chain model (4.1- and 1.15-fold increased median period sensitivity, respectively, 1.2- and 1.04-fold reduced median amplitude sensitivity, respectively, p-values <10−5, Tables R, S in the S1 File). Thus, the FitzHugh-Nagumo model displays rather low amplitude sensitivities and high period sensitivities. The implementation of the λ-ω oscillator shown here has period sensitivities which are higher than those of the negative feedback chain model and lower than those of the positive feedback chain model (3.3-fold increased and 1.1-fold reduced median period sensitivity, respectively, p-values <10−5, Tables R, S in the S1 File). The amplitude sensitivities are lower than for both chain models (1.5- and 1.3-fold reduced median amplitude sensitivity, respectively, p-valu [file pcbi.1005298.s010.tif]

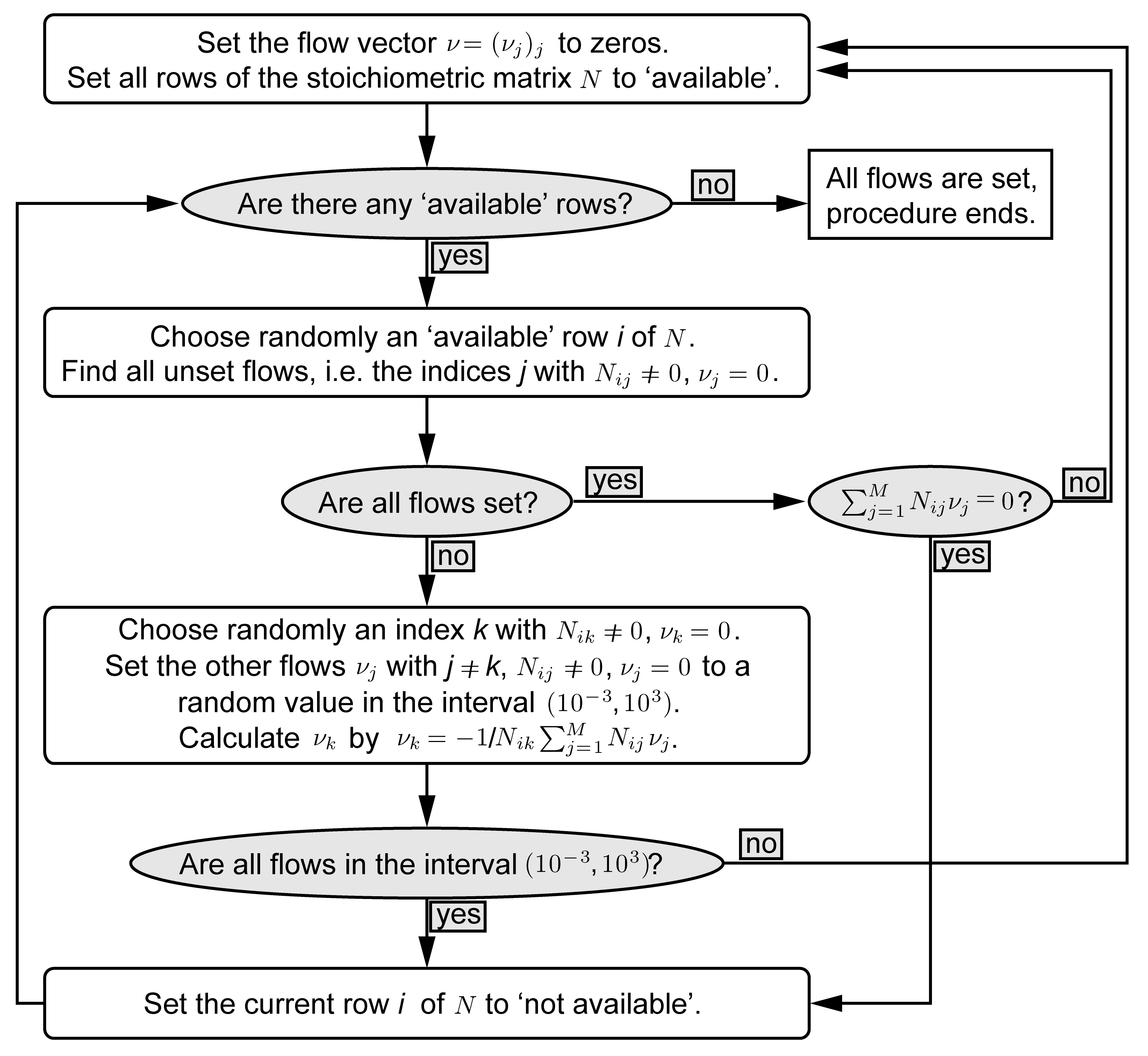

Supplement: S11 Fig — Basis is the condition N·ν = 0 for ν being the flow vector at steady state for the stoichiometric m×M-matrix N = (ηij) which is formed by the stoichiometric coefficients. The order in which the flows are set has to be chosen randomly in order to avoid bias upon the sampling results. This procedure is explained in the decision diagram. To accelerate the sampling process, independent parts of the stoichiometric matrix N and according sets of flows can be sampled separately. (TIF) [file pcbi.1005298.s011.tif]

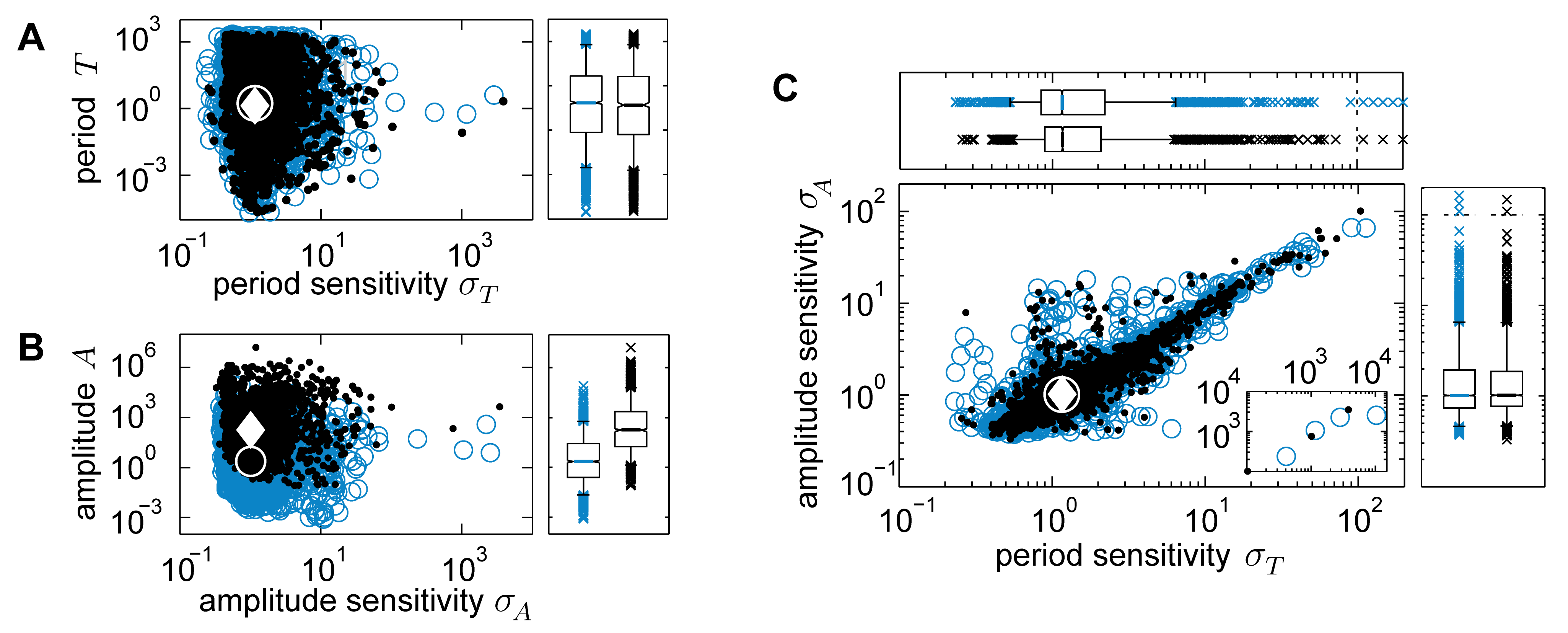

Supplement: S12 Fig — Examined is the phenomenological calcium oscillation model [38]. Blue circles, median values given by black circles: original sampling procedure in the interval (10−3, 103), black dots, median values given by white diamonds: results when sampling in the interval (10−1, 105). A: Period T versus period sensitivity σT. B: Amplitude A versus amplitude sensitivity σA. C: Period sensitivity σT versus amplitude sensitivity σA. Altering the sampling interval results in only minor alterations of the obtained periods (A) but increased amplitudes (B). The sensitivities are not influenced by the sampling interval, since they are derived from relative changes of period and amplitude (C, Tables T, U in the S1 File). (TIF) [file pcbi.1005298.s012.tif]

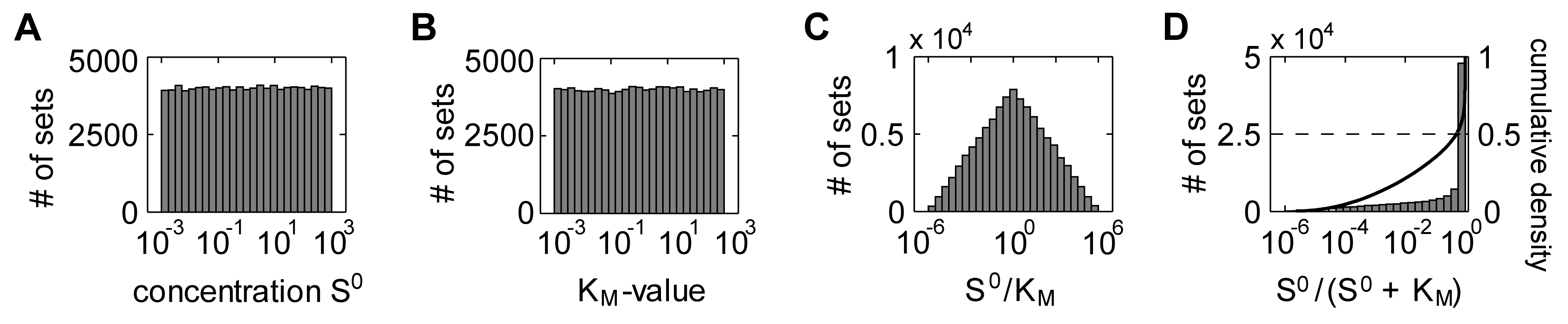

Supplement: S13 Fig — A: Histogram for a sampled steady state species concentration S0. B: Histogram for a sampled KM-value. C: Calculated value of the ratio between steady state concentration S0 and KM-value. D: Calculated Michaelis-Menten term S0/(S0 + KM) and the estimated cumulative probability function (solid line). A cumulative probability of 0.5 is indicated by the dashed line. The sampling ensures a log10-uniform distribution for the steady state concentrations and the nl-parameters. By choosing the same sampling interval and sampling distribution for these quantities, their ratios are symmetrically distributed with a central value of 1 (see C). The resulting Michaelis-Menten terms take values between 0 and 1. They are in 50% of the sampling cases lower and in 50% higher than 0.5 (see D). (TIF) [file pcbi.1005298.s013.tif]

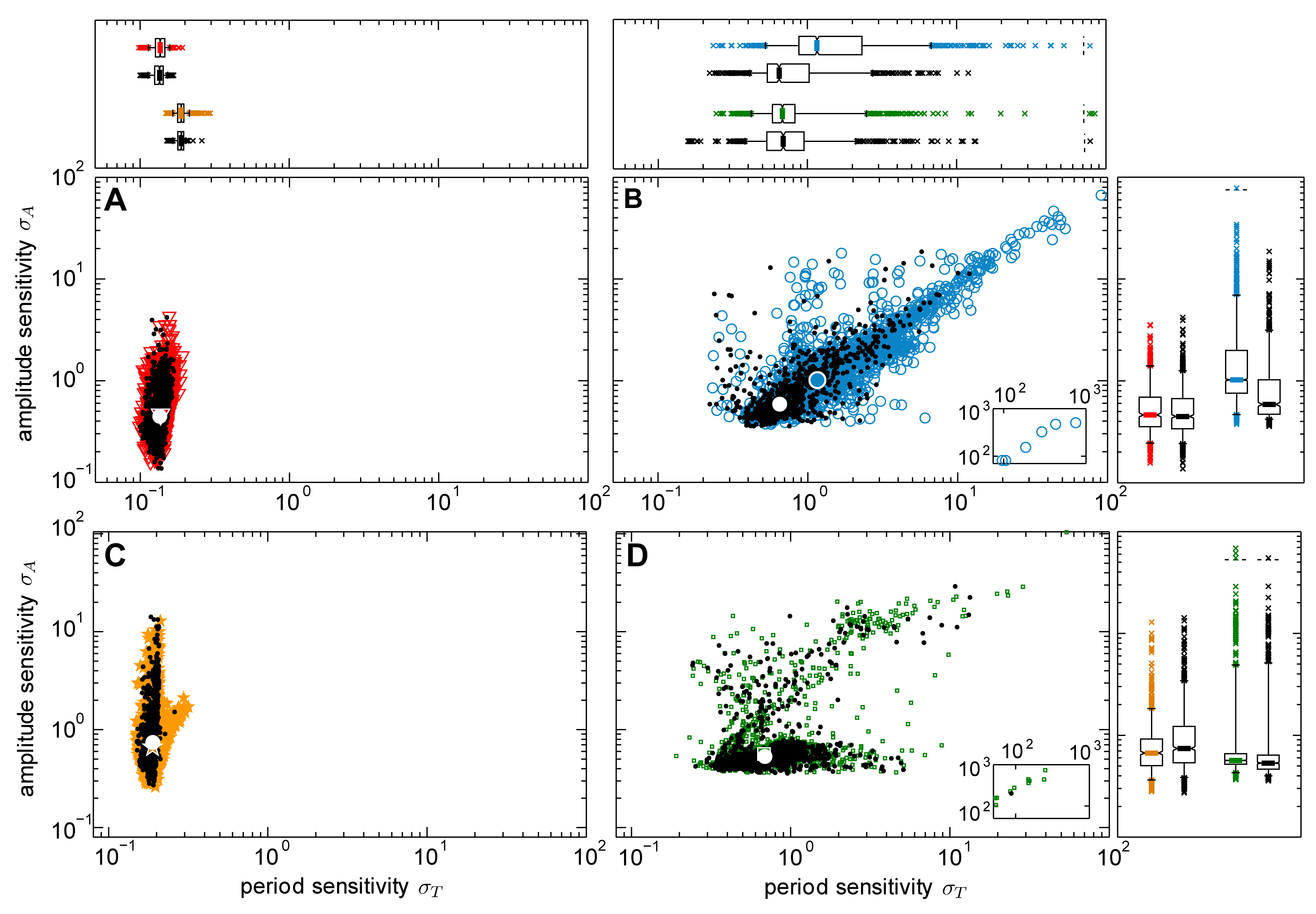

Supplement: S14 Fig — Given are the sensitivities of the mammalian circadian model [37] (A), the phenomenological calcium model [38] (B), the negative feedback chain model with linear kinetics (C) and the positive feedback chain model with linear kinetics (D) for the sampling procedure as proposed in the manuscript (colored symbols, median values are given by colored symbols with white frame) compared to the sensitivities of 1 000 parameter sets sampled using a top-down sampling approach (black dots, median values given by white circles). The top-down approach includes (i) sampling the nl-parameters, rate coefficients and initial concentrations log10-uniformly in the interval (10−3, 103), (ii) performing simulations starting from the sampled initial conditions, (iii) checking for oscillations, and (iv) perturbing the individual parameters followed by simulations to estimate the sensitivity coefficients. The box-plots show the distribution characteristics of the period sensitivities (top) and amplitude sensitivities (right) for 1 000 parameter sets. As can be seen in panels A-D the distribution characteristics are similar for both sampling approaches. The regions occupied in sensitivity space, these are the regions filled by the scatter plots, are highly consistent between the two approaches. The only slight deviation between the approaches is found for the median values in panel B. Since the sampling approach proposed here makes use of stability properties of the steady states, it requires much less effort concerning numerical integration (compare Table A in the S1 File) and is thus the more efficient method. (TIF) [file pcbi.1005298.s014.tif]

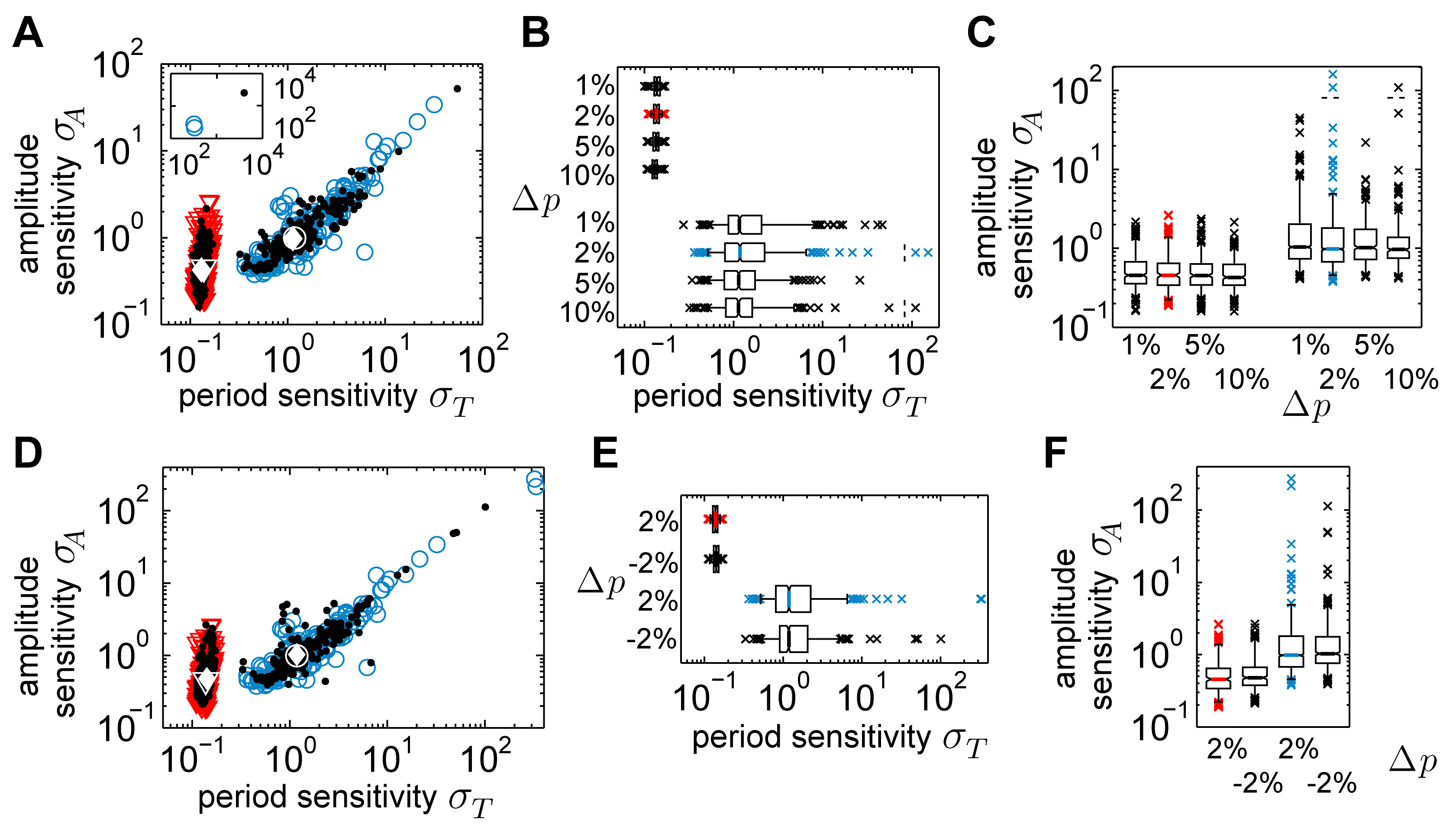

Supplement: S15 Fig — A: Results of the sensitivity analysis for the model of mammalian circadian oscillations and the phenomenological calcium model with a parameter perturbation Δp = 2% (blue: calcium model, red: circadian model, median values given by black symbols) or Δp = 10% (black dots, median values given by white diamonds). B and C: Period and amplitude sensitivity distributions, respectively, as box-plots for different parameter perturbation values (Δp = 1%, 2%, 5%, 10%). The according distribution for Δp = 2% is depicted in color (red: circadian model, blue: calcium model). D: Results of the sensitivity analysis for both models with Δp = 2% (red: circadian model, blue: calcium model, median values given by black symbols) or Δp = −2% (black dots, median values given by white diamonds). E and F: Period and amplitude sensitivity distributions, respectively, from panel D as box-plots. For all analyses, we compared sensitivity values for 250 parameter sets. Altering the parameter perturbation does not induce significant changes concerning the calcium model or the amplitude sensitivities of the circadian model (with a confidence level of 0.01, p-values of the Mann-Whitney-U test >0.035, Table W in the S1 File). The median period sensitivity of the circadian rhythm model is only slightly altered by 2.9% for Δp = -2%, and it is affected for Δp = 10%. However, no substantial gain in information is obtained for different parameter perturbations therefore only perturbations of Δp = 2% are applied. (TIF) [file pcbi.1005298.s015.tif]
