# Supplementary material for: Feedback, Mass Conservation and Reaction Kinetics Impact the Robustness of Cellular Oscillations
Source: PLoS Comput Biol. 2016 Dec 27;12(12):e1005298. doi: 10.1371/journal.pcbi.1005298 (PMC5226835; doi:10.1371/journal.pcbi.1005298)
Supplement: S1 File — The characteristics for the sensitivity distributions and the detailed statistics of the Mann-Whitney-U tests are given. (PDF) [file pcbi.1005298.s016.pdf]

S1 File, Baum et al.: Supplementary Tables

Table A: Comparison of the performance of the bottom-up and a top-down parameter sampling approach. The top-down approach includes (i) sampling the nl-parameters, rate coefficients and initial concentrations log10-uniformly in the interval $(10^{-3}, 10^{3})$, (ii) performing simulations starting from the sampled initial conditions, (iii) checking for oscillations, and (iv) perturbing the individual parameters followed by simulations to estimate the sensitivity coefficients.
The computational efforts for the two sampling methods for the mammalian circadian model (circ), the phenomenological calcium model (ca) and the chain models with conversions and linear kinetics with negative (neg) or positive (pos) feedback are compared. Indicated are the numbers of parameter sets which had to be sampled in order to obtain the sensitivities for 1000 parameter sets (# of sets sampled), the resulting success rate, i.e. the percentage of parameter sets for which the sensitivity could be estimated. Additionally, the number of parameter sets for which a numerical integration was performed (# of sets simulated) is given and the according success rate among those.
While for the top-down sampling, the models were numerically integrated for all sampled parameter sets, the bottom-up sampling required integration only for those parameter sets with unstable steady state. Consequently, the bottom-up sampling required much less computational effort as simulations had to be performed for a lower number of parameter sets than if using the top-down sampling approach. Numbers correspond to the sensitivity analyses in Fig. S14.

model	sampling method	# of sets sampled	success rate	# of sets simulated	success rate of simulated sets
circ	bottom-up	245000	40.82%	23300	4.29%
	top-down	464000	0.22%	464000	0.22%
ca	bottom-up	28600	3.50%	3060	32.68%
	top-down	848000	0.12%	848000	0.12%
neg	bottom-up	7890000	0.013%	4680	21.37%
	top-down	3650000	0.027%	3650000	0.027%
pos	bottom-up	59000	1.69%	10300	9.71%
	top-down	335000	0.30%	335000	0.30%

Sensitivity Tables

In the following tables, the detailed characteristics of the period sensitivity distributions (per.) and amplitude sensitivity distributions (ampl.) of the sensitivity analyses

performed in this work are given. The caption of each table delivers a short description of the captured models. Given are the identifier of the model (id), the median sensitivity (μ), the 95% confidence interval of the median (95% CI), the interval formed by the first and third quartiles ($[Q_{25}, Q_{75}]$), the interval formed by the fifth and 95th percentiles ($[Q_5, Q_{95}]$), the length of the 90% data range (90%R), Spearman's rank correlation coefficient (R_S) for the correlation between period and amplitude sensitivity, the number of parameter sets being sampled in order to analyze the sensitivities of 2500 parameter sets (# sets).

The detailed results of the Mann-Whitney-U (MWU) test indicating the significance of location differences between sensitivity distributions are indicated in separate tables. The p-value (MWU p), the numbers of parameter sets compared (n_X, n_Y), the calculated value of the U statistics (U) and the value for the normal distribution with according mean and standard deviation (z-val) are delivered.

Mammalian circadian model and phenomenological calcium model

Table B: Sensitivity distribution characteristics of the mammalian circadian oscillations model [1] (circ M) and the phenomenological calcium oscillations model [2] (ca phen). Data correspond to Fig. 1.

id	sens.	μ	95% CI	$[Q_{25}$,$Q_{75}]$	$[Q_5$,$Q_{95}]$	90%R	R_S	# sets
circ M	per.	0.14	[0.14,0.14]	[0.13,0.15]	[0.11,0.16]	0.05	0.52	$6.1 \cdot 10^5$
	ampl.	0.47	[0.46,0.48]	[0.35,0.68]	[0.24,1.37]	1.13		
ca phen	per.	1.16	[1.14,1.17]	[0.85,2.22]	[0.53,6.45]	5.92	0.82	$7.1 \cdot 10^4$
	ampl.	1.01	[1.00,1.03]	[0.74,1.94]	[0.46,6.54]	6.08		

Table C: Results for the MWU test comparing the mammalian circadian [1] (circ M) and the phenomenological calcium oscillations model [2] (ca phen). Data correspond to Fig. 1.

id_X		id_Y	sens.	MWU p	n_X	n_Y	U	z-val
circ M	⇔	ca phen	per.	0	2500	2500	3126250	-61.23
circ M	⇔	ca phen	ampl.	0	2500	2500	4105063	-42.05

Chain models with positive or negative feedback

Table D: Sensitivity distribution characteristics of the chain models with mass action kinetics, conversions and negative (neg fb) or positive feedback (pos fb). Data correspond to Fig. 2.

id	sens.	μ	95% CI	[Q_{25},Q_{75}]	[Q_5,Q_{95}]	90%R	R_S	# sets
neg fb	per.	0.19	0.19	[0.18,0.20]	[0.17,0.22]	0.05	0.30	$2.0 \cdot 10^7$
	ampl.	0.66	[0.65,0.68]	[0.50,0.93]	[0.35,1.84]	1.49		
pos fb	per.	0.68	[0.67, 0.69]	[0.58,0.82]	[0.41,2.40]	1.99	0.42	$1.5 \cdot 10^5$
	ampl.	0.57	[0.56,0.57]	[0.51,0.65]	[0.43,4.91]	4.48		

Table E: Results for the MWU test comparing the sensitivity distributions of the chain models with mass action kinetics, conversions and negative (neg fb) or positive feedback (pos fb). Data correspond to Fig. 2.

id_X		id_Y	sens.	MWU p	n_X	n_Y	U	z-val
neg fb	⇔	pos fb	per.	0	2500	2500	1420	-61.20
pos fb	⇔	neg fb	ampl.	$2.5 \cdot 10^{-24}$	2500	2500	2609029	-10.11

Chain models with mass action kinetics or Michaelis-Menten kinetics

Table F: Sensitivity distribution characteristics of the chain models with mass action kinetics in all eight reactions (ma), with Michaelis-Menten kinetics only in the degradation reactions (deg MM), with Michaelis-Menten kinetics only in the conversion reactions (conv MM), or with Michaelis-Menten kinetics in conversion and degradation reactions (MM) and negative (neg fb) or positive feedback (pos fb). Data correspond to Fig. 3.

	id	sens.	μ	95% CI	[Q_{25},Q_{75}]	[Q_5,Q_{95}]	90%R	R_S	# sets
neg fb	ma	per.	0.19	[0.19,0.19]	[0.18,0.20]	[0.17,0.22]	0.05	0.30	$2.0 \cdot 10^7$
		ampl.	0.66	[0.65,0.68]	[0.50,0.93]	[0.35,1.84]	1.49		
	deg MM	per.	0.30	[0.29,0.30]	[0.23,0.47]	[0.19,1.74]	1.55	0.49	$3.7 \cdot 10^5$
		ampl.	0.60	[0.57,0.62]	[0.42,1.22]	[0.32,5.41]	5.09		
	conv MM	per.	0.22	[0.21,0.22]	[0.19,0.27]	[0.16,1.15]	0.99	0.52	$7.7 \cdot 10^7$
		ampl.	0.88	[0.85,0.91]	[0.57,1.67]	[0.35,6.28]	5.93		
	MM	per.	0.66	[0.63,0.71]	[0.35,1.64]	[0.23,6.55]	6.32	0.76	$1.4 \cdot 10^6$
		ampl.	1.34	[1.26,1.41]	[0.71,3.01]	[0.43,8.97]	8.54		
pos fb	ma	per.	0.68	[0.67, 0.69]	[0.58,0.82]	[0.41,2.40]	1.99	0.42	$1.5 \cdot 10^5$
		ampl.	0.57	[0.56,0.57]	[0.51,0.65]	[0.43,4.91]	4.48		
	deg MM	per.	0.67	[0.65, 0.68]	[0.52,1.25]	[0.35,7.45]	7.10	0.10	$1.4 \cdot 10^5$
		ampl.	0.52	[0.52,0.53]	[0.43,0.62]	[0.36,1.45]	1.09		
	conv MM	per.	1.17	[1.12, 1.23]	[0.69,2.67]	[0.45,13.73]	13.28	0.75	$2.1 \cdot 10^6$
		ampl.	1.00	[0.94,1.06]	[0.58,2.71]	[0.44,13.83]	13.39		
	MM	per.	1.33	[1.26, 1.40]	[0.70,3.26]	[0.42,13.34]	12.92	0.64	$1.7 \cdot 10^6$
		ampl.	0.98	[0.94,1.03]	[0.60,2.38]	[0.43,11.71]	11.28		

Table G: Results for the MWU test comparing the sensitivity distributions of the chain models with mass action kinetics (ma) or Michaelis-Menten kinetics only in the degradation reactions (deg MM), Michaelis-Menten kinetic only in the conversion reactions (conv MM) or Michaelis-Menten kinetics in all eight reactions (MM) for negative feedback (neg fb) or positive feedback (pos fb). Data correspond to Fig. 3.

	id_X		id_Y	sens.	MWU p	n_X	n_Y	U	z-val
neg fb	ma	⇔	deg MM	per.	0	2500	2500	442898	-52.55
	deg MM	⇔	ma	ampl.	$5.9 \cdot 10^{-3}$	2500	2500	2996541	-2.52
	ma	⇔	conv MM	per.	$3.9 \cdot 10^{-233}$	2500	2500	1462234	-32.58
				ampl.	$1.1 \cdot 10^{-61}$	2500	2500	2281420	-16.53
	ma	⇔	MM	per.	0	2500	2500	39848	-60.45
				ampl.	$1.6 \cdot 10^{-207}$	2500	2500	1557243	-30.72
	deg MM	⇔	MM	per.	$5.4 \cdot 10^{-215}$	2500	2500	1528937	-31.27
				ampl.	$1.4 \cdot 10^{-156}$	2500	2500	1 1765688	-26.63
	conv MM	⇔	MM	per.	0	2500	2500	800311	-45.55
				ampl.	$3.9 \cdot 10^{-47}$	2500	2500	1 2391499	-14.37
pos fb	ma	⇔	deg MM	per.	0.29	2500	2500	3096911	-0.55
	deg MM	⇔	ma	ampl.	$4.8 \cdot 10^{-61}$	2500	2500	2285861	-16.44
	ma	⇔	conv MM	per.	$1.3 \cdot 10^{-161}$	2500	2500	1743690	-27.07
				ampl.	$4.2 \cdot 10^{-164}$	2500	2500	1732994	-27.27
	ma	⇔	MM	per.	$3.8 \cdot 10^{-173}$	2500	2500	1694628	-28.03
				ampl.	$1.2 \cdot 10^{-175}$	2500	2500	1684145	-28.23
	deg MM	⇔	MM	per.	$1.2 \cdot 10^{-104}$	2500	2500	2017862	-21.69
				ampl.	$2.6 \cdot 10^{-306}$	2500	2500	1216625	-37.39
	conv MM	⇔	MM	per.	$5.2 \cdot 10^{-3}$	2500	2500	2994171	-2.56
	MM	⇔	conv MM	ampl.	0.26	2500	2500	3092481	-0.64

Chain models with conversions or regulated productions

Table H: Sensitivity distribution characteristics of the chain models with mass action kinetics and different mass conservation properties. The models with conversion in reactions 2, 4, and 6 with negative feedback (neg fb conv) or positive feedback (pos fb conv) correspond to the chain models from Fig. 2. Models lacking mass conservation in some reactions are the model with negative feedback and regulated productions in reactions 2, 4 and 6 (neg fb reg prod) and the model with positive feedback and regulated productions in reactions 4 and 6 (pos fb reg prod). Data correspond to Fig. 4.

id	sens.	μ	95% CI	[Q_{25},Q_{75}]	[Q_5,Q_{95}]	90%R	R_S	# sets
neg fb	per.	0.189	[0.188,190]	[0.18,0.20]	[0.17,0.22]	0.05	0.30	$2.0 \cdot 10^7$
conv	ampl.	0.66	[0.65,0.68]	[0.50,0.93]	[0.35,1.84]	1.49		
neg fb	per.	0.198	0.198	[0.195,0.201]	[0.19,0.204]	0.012	0.94	$5.2 \cdot 10^6$
reg prod	ampl.	0.71	[0.69,0.73]	[0.52,1.04]	[0.36,1.79]	1.43		
pos fb	per.	0.68	[0.67,0.69]	[0.58,0.82]	[0.41,2.40]	1.99	0.42	$1.5 \cdot 10^5$
conv	ampl.	0.57	[0.56,0.57]	[0.51,0.65]	[0.43,4.91]	4.48		
pos fb	per.	0.93	[0.92,0.93]	[0.76,1.17]	[0.56,3.06]	2.50	0.14	$2.5 \cdot 10^5$
reg prod	ampl.	0.68	[0.67,0.69]	[0.56,0.79]	[0.45,5.65]	5.20		

Table I: Results for the MWU test comparing the sensitivity distributions of the chain models with mass conservation in reactions 2, 4, and 6 with negative feedback (neg fb conv) or positive feedback (pos fb conv) to the model with negative feedback and regulated productions in reactions 2, 4 and 6 (neg fb reg prod) or the model with positive feedback and regulated productions in reactions 4 and 6 (pos fb reg prod), respectively. Data correspond to Fig. 4.

id_X		id_Y	sens.	MWU p	n_X	n_Y	U	z-val
neg fb	⇔	neg fb	per.	$1.5 \cdot 10^{-259}$	2500	2500	1369600	-34.3952
conv		reg prod	ampl.	$3.6 \cdot 10^{-6}$	2500	2500	2895868	-4.4896
pos fb	⇔	pos fb	per.	$3.3 \cdot 10^{-180}$	2500	2500	1665321	-28.6009
conv		reg prod	ampl.	$7.5 \cdot 10^{-89}$	2500	2500	2106821	-19.9502

Circadian and calcium oscillations models

Table J: Table for the sensitivity distribution characteristics of the circadian rhythm models from [1] (circ M), from [3] (circ D), from [4] (circ A); of the calcium oscillation models from [2] (ca phen), from [5] (ca open), from [6] (ca closed). In addition to the characteristics provided above, the sensitivities obtained for the reference parameter set published together with the model are given (σ^*). Data correspond to Fig. 5.

id	sens.	σ^*	μ	95% CI	[Q_{25},Q_{75}]	[Q_5,Q_{95}]	90%R	R_S	# sets
circ M	per.	0.11	0.14	[0.14,0.14]	[0.13,0.15]	[0.11,0.16]	0.05	0.52	$6.1 \cdot 10^5$
	ampl.	0.62	0.47	[0.46,0.48]	[0.35,0.68]	[0.24,1.37]	1.13		
circ D	per.	0.27	0.49	[0.45,0.53]	[0.26,1.49]	[0.20,8.03]	7.83	0.81	$1.7 \cdot 10^5$
	ampl.	1.11	0.97	[0.91,1.03]	[0.52,2.45]	[0.35,9.64]	9.29		
circ A	per.	0.22	0.44	[0.42,0.46]	[0.24,1.02]	[0.15,4.23]	4.08	0.65	$3.5 \cdot 10^5$
	ampl.	2.80	0.96	[0.91,1.03]	[0.46,2.44]	[0.25,8.67]	8.42		
ca phen	per.	1.64	1.16	[1.14,1.17]	[0.85,2.22]	[0.53,6.45]	5.92	0.82	$7.1 \cdot 10^4$
	ampl.	1.59	1.01	[1.00,1.03]	[0.74,1.94]	[0.46,6.54]	6.08		
ca open	per.	0.55	0.46	[0.46,0.47]	[0.37,0.63]	[0.29,2.62]	2.33	0.61	$5.9 \cdot 10^4$
	ampl.	0.22	0.36	[0.35,0.36]	[0.26,0.48]	[0.17,1.12]	0.95		
ca closed	per.	0.30	0.55	[0.53,0.57]	[0.36,0.87]	[0.19,2.25]	2.04	0.16	$1.5 \cdot 10^5$
	ampl.	1.33	0.36	[0.35,0.37]	[0.21,0.57]	[0.12,1.79]	1.67		

Table K: Results for the MWU test comparing the period sensitivity distributions between the circadian oscillations models (circ), between the calcium oscillations models (ca), and between calcium and circadian oscillations models (ca/circ), as well as the according amplitude sensitivity distributions. Circadian models: from [1] (circ M), from [3] (circ D), from [4] (circ A). Calcium models: from [2] (ca phen), from [5] (ca open), from [6] (ca closed). Data correspond to Fig. 5.

	$\mathrm{id_X}$		$\mathrm{id_Y}$	sens.	MWU p	n_X	n_Y	U	z-val
circ	circ M	⇔	circ D	per.	0	2500	2500	6642	-61.1
	circ M	⇔	circ A	per.	0	2500	2500	238492	-56.6
	circ A	⇔	circ D	per.	$7.5 \cdot 10^{-11}$	2500	2500	2798087	-6.4
ca	ca open	⇔	ca phen	per.	0	2500	2500	850223	-44.5
	ca closed	⇔	ca phen	per.	0	2500	2500	1189860	-37.9
	ca open	⇔	ca closed	per	$5.2 \cdot 10^{-10}$	2500	2500	2813457	-6.1
ca/ circ	circ M	⇔	ca phen	per.	0	2500	2500	0	-61.23
	circ M	⇔	ca open	per.	0	2500	2500	101	-61.2
	circ M	⇔	ca closed	per.	0	2500	2500	74751	-59.8
	circ D	⇔	ca phen	per.	$6.7 \cdot 10^{-155}$	2500	2500	1773167	-26.5
	circ D	⇔	ca open	per.	0.13	2500	2500	3067850	-1.1
	circ D	⇔	ca closed	per.	0.20	2500	2500	3081382	-0.85
	circ A	⇔	ca phen	per.	$5.2 \cdot 10^{-251}$	2500	2500	1399002	-33.8
	circ A	⇔	ca open	per.	$1.0 \cdot 10^{-8}$	2500	2500	2838595	-5.6
	circ A	⇔	ca closed	per.	$3.2 \cdot 10^{-12}$	2500	2500	2774426	-6.9
circ	circ M	⇔	circ D	ampl.	$3.9 \cdot 10^{-238}$	2500	2500	1444295	-32.9
	circ M	⇔	circ A	ampl.	$4.6 \cdot 10^{-159}$	2500	2500	1754824	-26.8
	circ A	⇔	circ D	ampl.	$8.8 \cdot 10^{-4}$	2500	2500	2965299	-3.1
ca	ca open	⇔	ca phen	ampl.	0	2500	2500	522624	-51.0
	ca closed	⇔	ca phen	ampl.	0	2500	2500	824589	-45.1
	ca closed	⇔	ca open	ampl	0.089	2500	2500	3056252	-1.3
ca/ circ	circ M	⇔	ca phen	ampl.	0	2500	2500	978813	-42.1
	ca open	⇔	circ M	ampl.	$3.4 \cdot 10^{-96}$	2500	2500	2064547	-20.8
	ca closed	⇔	circ M	ampl.	$2.1 \cdot 10^{-68}$	2500	2500	2234986	-17.4
	circ D	⇔	ca phen	ampl.	$2.8 \cdot 10^{-5}$	2500	2500	2919224	-4.0
	ca open	⇔	circ D	ampl.	0	2500	2500	855800	-44.4
	ca closed	⇔	circ D	ampl.	0	2500	2500	1099431	-39.7
	circ A	⇔	ca phen	ampl.	$7.6 \cdot 10^{-9}$	2500	2500	2836190	-5.7
	ca open	⇔	circ A	ampl.	0	2500	2500	1193590	-37.8
	ca closed	⇔	circ A	ampl.	$1.3 \cdot 10^{-270}$	2500	2500	1332216	-35.1

Additional models of biological oscillations

Table L: Table for the sensitivity distribution characteristics of additional models with originally published and altered types of reaction kinetics. The repressilator with mass action kinetics [7] (repr ma), and with Michaelis-Menten kinetics (repr MM); the MAPK oscillator with Michaelis-Menten kinetics [8] (MAPK MM), and with mass action kinetics (MAPK ma); the glycolytic oscillations model with mass action kinetics [9, 10] (glyc ma), and with Michaelis-Menten kinetics (glyc MM); the cell cycle oscillations model with mass action kinetics [11] (cycle ma), and with Michaelis-Menten kinetics (cycle MM). Data correspond to Fig. 7.

id	sens.	μ	95% CI	[Q_{25},Q_{75}]	[Q_5,Q_{95}]	90%R	R_S	# sets
repr ma	per.	0.16	[0.16,0.16]	[0.13,0.20]	[0.11,0.27]	0.16	0.45	$2.4 \cdot 10^7$
	ampl.	0.92	[0.90,0.94]	[0.67,1.23]	[0.45,2.27]	1.82		
repr MM	per.	1.48	[1.38,1.57]	[0.65,3.58]	[0.29,11.41]	11.12	0.65	$5.6 \cdot 10^6$
	ampl.	3.06	[2.86,3.26]	[1.41,7.09]	[0.55,17.97]	17.42		
MAPK ma	per.	0.19	[0.19,0.19]	[0.18,0.20]	[0.16,0.23]	0.07	0.50	$6.1 \cdot 10^7$
	ampl.	0.65	[0.62,0.67]	[0.43,1.07]	[0.27,2.17]	1.9		
MAPK MM	per.	0.32	[0.31,0.33]	[0.22,0.54]	[0.16,2.00]	1.84	0.42	$1.8 \cdot 10^5$
	ampl.	0.60	[0.56,0.64]	[0.23,1.75]	[0.02,6.70]	6.68		
glyc ma	per.	4.22	[4.17,4.29]	[3.66,5.70]	[3.33,9.75]	6.42	0.03	$7.6 \cdot 10^5$
	ampl.	19.6	[19.5,19.7]	[18.6,21.6]	[18.3,27.6]	8.3		
glyc MM	per.	4.64	[4.57,4.73]	[3.86,6.15]	[3.44,9.63]	6.19	0.47	$6.3 \cdot 10^6$
	ampl.	19.3	[19.2,19.4]	[18.0,21.7]	[17.4,29.2]	11.8		
cycle ma	per.	0.65	[0.64,0.67]	[0.50,0.87]	[0.32,2.14]	1.82	0.42	$1.2 \cdot 10^5$
	ampl.	0.46	[0.46,0.47]	[0.35,0.51]	[0.29,1.42]	1.13		
cycle MM	per.	0.80	[0.77,0.83]	[0.53,1.31]	[0.28,4.17]	3.89	0.48	$3.3 \cdot 10^5$
	ampl.	0.59	[0.58,0.61]	[0.45,0.88]	[0.31,3.25]	2.94		

Table M: Results for the MWU test for comparing the sensitivities of the repressilator model with mass action kinetics [7] (repr ma) to that with Michaelis-Menten kinetics (repr MM); the MAPK oscillator with Michaelis-Menten kinetics [8] (MAPK MM) to that with mass action kinetics (MAPK ma); the glycolytic oscillations model with mass action kinetics [9, 10] (glyc ma) to that with Michaelis-Menten kinetics (glyc MM); the cell cycle oscillations model with mass action kinetics [11] (cycle ma) to that with Michaelis-Menten kinetics (cycle MM). Data correspond to Fig. 7.

id_X		id_Y	sens.	MWU p	n_X	n_Y	U	z-val
repr ma	⇔	repr MM	per.	0	2500	2500	44392	-60.4
			ampl.	0	2500	2500	1067165	-40.3
MAPK ma	⇔	MAPK MM	per.	0	2500	2500	828972	-44.99
MAPK MM	⇔	MAPK ma	ampl.	$2.8 \cdot 10^{-4}$	2500	2500	2948704	-3.45
glyc ma	⇔	glyc MM	per.	$1.3 \cdot 10^{-17}$	2500	2500	2693308	-8.46
glyc MM	⇔	glyc ma	ampl.	$3.7 \cdot 10^{-15}$	2500	2500	2727992	-7.78
cycle ma	⇔	cycle MM	per.	$2.3 \cdot 10^{-32}$	2500	2500	2523507	-11.79
			ampl.	$3.3 \cdot 10^{-139}$	2500	2500	1844568	-25.09

Tables for Supplementary Figures

Altered sample size

Table N: Sensitivity values of the phenomenological calcium model [2] (ca) and the mammalian circadian model [1] (circ) for a sample size (s.s.) of 2500 and 75000. Data correspond to Fig. S1.

id	s.s.	sens.	μ	95% CI	[Q_{25},Q_{75}]	[Q_5,Q_{95}]	90%R	R_S	# sets
ca	2500	per.	1.16	[1.14,1.17]	[0.85,2.22]	[0.53,6.45]	5.92	0.82	$7.1 \cdot 10^4$
		ampl.	1.01	[1.00,1.03]	[0.74,1.94]	[0.46,6.54]	6.08		
ca	75000	per.	1.17	[1.17,1.17]	[0.86,2.15]	[0.53,6.11]	5.58	0.83	$2.1 \cdot 10^6$
		ampl.	1.01	[1.16,1.18]	[0.86,2.14]	[0.53,6.07]	5.54		
circ	2500	per.	0.14	[0.14,0.14]	[0.13,0.15]	[0.11,0.16]	0.05	0.52	$6.1 \cdot 10^5$
		ampl.	0.47	[0.46,0.48]	[0.35,0.68]	[0.24,1.37]	1.13		
circ	75000	per.	0.14	[0.14,0.14]	[0.13,0.15]	[0.12,0.16]	0.04	0.52	$1.9 \cdot 10^7$
		ampl.	0.46	[0.46,0.47]	[0.35,0.69]	[0.24,1.36]	1.12		

Positive feedback chain model with Hill coefficient of $n = 9$

Table O: Sensitivity distribution characteristics of the chain model with positive feedback, mass action kinetics, mass conversions and a Hill coefficient of $n = 9$ (pos $n = 9$) instead of $n = 2$. Data correspond to Fig. S2.

id	sens.	μ	95% CI	[Q_{25},Q_{75}]	[Q_5,Q_{95}]	90%R	R_S	# sets
pos	per.	0.78	[0.76,0.81]	[0.47,1.27]	[0.23,3.04]	2.81	0.09	$9.9 \cdot 10^5$
$n = 9$	ampl.	0.49	[0.48,0.49]	[0.44,0.57]	[0.40,2.13]	1.73		

Chain models with altered mass conservation properties and kinetics

Table P: Sensitivity distribution characteristics of the chain models with mass action kinetics (ma) or Michaelis-Menten kinetics (MM) in all reactions and different mass conservation properties. The models with mass conversions in reactions 2, 4, and 6 with negative feedback (N1, N2) or positive feedback (P1, P2) correspond to the chain models with mass action kinetics (N1, P1, see also Fig. 2 and according tables) or Michaelis-Menten kinetics (N2, P2, see also Fig. 3 and according tables). Models lacking mass conservation in some reactions are the models with negative feedback and regulated productions in reactions 2, 4 and 6 (N3, N4) and the models with positive feedback and regulated productions in reactions 4 and 6 (P3, P4) with mass action kinetics (N3, P3, see also Fig. 4 and according tables) or Michaelis-Menten kinetics (N4, P4). Data correspond to Fig. S8.

id	sens.	μ	95% CI	$[Q_{25},Q_{75}]$	$[Q_5,Q_{95}]$	90%R	R_S	# sets
N1	per.	0.19	[0.19,0.19]	[0.18,0.20]	[0.17,0.22]	0.05	0.30	$2.0 \cdot 10^7$
	ampl.	0.66	[0.65,0.68]	[0.50,0.93]	[0.35,1.84]	1.49		
N2	per.	0.66	[0.63,0.71]	[0.35,1.64]	[0.23,6.55]	6.32	0.76	$1.4 \cdot 10^6$
	ampl.	1.34	[1.26,1.41]	[0.71,3.01]	[0.43,8.97]	8.54		
N3	per.	0.198	0.198	[0.195,0.201]	[0.19,0.204]	0.012	0.94	$5.2 \cdot 10^6$
	ampl.	0.71	[0.69,0.73]	[0.52,1.04]	[0.36,1.79]	1.43		
N4	per.	0.56	[0.54,57]	[0.37,1.04]	[0.26,5.15]	4.89	0.67	$4.8 \cdot 10^5$
	ampl.	0.96	[0.93,1.00]	[0.67,1.71]	[0.45,6.09]	5.64		
P1	per.	0.68	[0.67, 0.69]	[0.58,0.82]	[0.41,2.40]	1.99	0.42	$1.5 \cdot 10^5$
	ampl.	0.57	[0.56,0.57]	[0.51,0.65]	[0.43,4.91]	4.48		
P2	per.	1.33	[1.26, 1.40]	[0.70,3.26]	[0.42,13.34]	12.92	0.64	$1.7 \cdot 10^6$
	ampl.	0.98	[0.94,1.03]	[0.60,2.38]	[0.43,11.71]	11.28		
P3	per.	0.93	[0.92,0.93]	[0.76,1.17]	[0.56,3.06]	2.50	0.14	$2.5 \cdot 10^5$
	ampl.	0.68	[0.67,0.69]	[0.56,0.79]	[0.45,5.65]	5.20		
P4	per.	1.74	[1.67,1.82]	[0.96,4.25]	[0.55,17.05]	16.50	0.49	$4.0 \cdot 10^6$
	ampl.	1.23	[1.17,1.28]	[0.72,2.72]	[0.43,10.58]	10.15		

Table Q: Results for the MWU test comparing the sensitivity distributions of the chain models with mass action kinetics (ma) or Michaelis-Menten kinetics (MM) and different mass conservation properties. The models with mass conversions in reactions 2, 4, and 6 with negative feedback (N1, N2) or positive feedback (P1, P2) correspond to the chain models with mass action kinetics (N1, P1, see also Fig. 2 and according tables) or Michaelis-Menten kinetics (N2, P2, see also Fig. 3 and according tables). Models lacking mass conservation in some reactions are the models with negative feedback and regulated productions in reactions 2, 4 and 6 (N3, N4) and the models with positive feedback and regulated productions in reactions 4 and 6 (P3, P4) with mass action kinetics (N3, P3, see also Fig. 4 and according tables) or Michaelis-Menten kinetics (N4, P4). Data correspond to Fig. S8.

id_X		id_Y	sens.	MWU p	n_X	n_Y	U	z-val
N1	⇔	N2	per.	0	2500	2500	39848	-60.45
			ampl.	$1.6 \cdot 10^{-207}$	2500	2500	1557243	-30.72
N1	⇔	N3	per.	$1.5 \cdot 10^{-259}$	2500	2500	1369600	-34.3952
			ampl.	$3.6 \cdot 10^{-6}$	2500	2500	2895868	-4.4896
N4	⇔	N2	per.	$2.16 \cdot 10^{-4}$	2500	2500	2945338	-3.52
			ampl.	$2.11 \cdot 10^{-26}$	2500	2500	2585674	-10.57
N3	⇔	N4	per.	0	2500	2500	13377	-60.97
			ampl.	$1.12 \cdot 10^{-92}$	2500	2500	2084585	-20.39
P1	⇔	P2	per.	$3.8 \cdot 10^{-173}$	2500	2500	1694628	-28.03
			ampl.	$1.2 \cdot 10^{-175}$	2500	2500	1684145	-28.23
P1	⇔	P3	per.	$3.3 \cdot 10^{-180}$	2500	2500	1665321	-28.6009
			ampl.	$7.5 \cdot 10^{-89}$	2500	2500	2106821	-19.9502
P2	⇔	P4	per.	$2.26 \cdot 10^{-24}$	2500	2500	2608537	-10.12
			ampl.	$1.25 \cdot 10^{-10}$	2500	2500	2802090	-6.33
P3	⇔	P4	per.	$1.38 \cdot 10^{-169}$	2500	2500	1709593	-27.73
			ampl.	$5.63 \cdot 10^{-175}$	2500	2500	1686965	-28.18

FitzHugh-Nagumo model and λ-ω oscillator

Table R: Table for the sensitivity distribution characteristics of the FitzHugh-Nagumo model of neural dynamics [12] (fitz) and an implementation of a λ-ω oscillator [13] (lambda). For comparison, data for the chain models with negative (neg fb ma) or positive feedback (pos fb ma) and mass action kinetics are shown. Data correspond to Fig. S10.

id	sens.	μ	95% CI	[Q_{25},Q_{75}]	[Q_5,Q_{95}]	90%R	R_S	# sets
fitz	per.	0.78	[0.77,0.79]	[0.63,0.89]	[0.29,1.45]	1.16	0.27	$3.8 \cdot 10^4$
	ampl.	0.55	[0.53,0.56]	[0.39,0.68]	[0.28,0.70]	0.42		
lambda	per.	0.62	[0.62,0.62]	[0.50,0.66]	[0.44,2.09]	1.65	0.02	$2.5 \cdot 10^3$
	ampl.	0.447	[0.447,0.447]	[0.447,0.447]	[0.446,0.448]	0.002		
neg fb ma	per.	0.19	0.19	[0.18,0.20]	[0.17,0.22]	0.05	0.30	$2.0 \cdot 10^7$
	ampl.	0.66	[0.65,0.68]	[0.50,0.93]	[0.35,1.84]	1.49		
pos fb ma	per.	0.68	[0.67, 0.69]	[0.58,0.82]	[0.41,2.40]	1.99	0.42	$1.5 \cdot 10^5$
	ampl.	0.57	[0.56,0.57]	[0.51,0.65]	[0.43,4.91]	4.48		

Table S: Results for the MWU test comparing the sensitivities of the FitzHugh-Nagumo model of neural dynamics [12] (fitz) and the sensitivities of an implementation of a λ-ω oscillator [13] (lambda) to those of the chain models with mass action kinetics and negative feedback (neg fb ma) or positive feedback (pos fb ma). Data correspond to Fig. S10.

id_X		id_Y	sens.	MWU p	n_X	n_Y	U	z-val
neg fb ma	⇔	fitz	per.	0	2500	2500	1799	-61.2
fitz	⇔	neg fb ma	ampl.	$1.2 \cdot 10^{-110}$	2500	2500	1985824	-22.3
pos fb ma	⇔	fitz	per.	$5.2 \cdot 10^{-19}$	2500	2500	2674315	-8.8
fitz	⇔	pos fb ma	ampl.	$1.8 \cdot 10^{-25}$	2500	2500	2595941	-10.4
neg fb ma	⇔	lambda	per.	0	2500	2500	0	-61.2
lambda	⇔	neg fb ma	ampl.	0	2500	2500	1079652	-40.1
lambda	⇔	pos fb ma	per.	$2.8 \cdot 10^{-54}$	2500	2500	2335520	-15.5
			ampl.	0	2500	2500	492780	-51.6

Altered sampling interval

Table T: Sensitivity values of the phenomenological calcium oscillations model [2] for two different sampling intervals: the standard interval $(10^{-3}, 10^{3})$ or the altered interval $(10^{-1}, 10^{5})$. Data correspond to Fig. S12.

id	sens.	μ	95% CI	[Q_{25},Q_{75}]	[Q_5,Q_{95}]	90%R	R_S	# sets
$(10^{-3}, 10^{3})$	per.	1.158	[1.14,1.17]	[0.85,2.22]	[0.53,6.45]	5.92	0.82	$7.1 \cdot 10^4$
	ampl.	1.014	[1.00,1.03]	[0.74,1.94]	[0.46,6.54]	6.08		
$(10^{-1}, 10^{5})$	per.	1.166	[1.15,1.19]	[0.89,2.09]	[0.55,6.30]	5.75	0.83	$6.7 \cdot 10^4$
	ampl.	1.018	[1.01,1.03]	[0.77,1.87]	[0.47,6.48]	6.01		

Table U: Results for the MWU test comparing the sensitivity distributions of the phenomenological calcium oscillations model [2] for the standard interval $(10^{-3}, 10^{3})$ and the altered interval $(10^{-1}, 10^{5})$. Data correspond to Fig. S12.

id_X		id_Y	sens.	MWU p	n_X	n_Y	U	z-val
$(10^{-3}, 10^{3})$	$\Leftrightarrow$	$(10^{-1}, 10^{5})$	per.	0.09	2500	2500	3056538	-1.3414
			ampl.	0.17	2500	2500	3077044	-0.9396

Sensitivity for top-down sampling

Table V: Sensitivity values of the mammalian circadian oscillations model [1] (circ), the phenomenological calcium oscillations model [2] (ca), and the chain models with mass action kinetics and negative (neg) or positive feedback (pos) for a top-down sampling approach (TD). In this approach, the nl-parameters, rate coefficients and initial concentrations were directly sampled. The sensitivity data for the bottom-up sampling (BU), also given in Tables B and D, are given for comparison. Data correspond to Fig. S14.

id	sens.	μ	95% CI	[Q_{25},Q_{75}]	[Q_5,Q_{95}]	90%R	R_S
circ TD	per.	0.136	[0.134,0.136]	[0.126,0.143]	[0.11,0.15]	0.04	0.38
	ampl.	0.45	[0.43,0.46]	[0.34,0.67]	[0.24,1.25]	1.01	
circ BU	per.	0.14	[0.14,0.14]	[0.13,0.15]	[0.11,0.16]	0.05	0.52
	ampl.	0.47	[0.46,0.48]	[0.35,0.68]	[0.24,1.37]	1.13	
ca TD	per.	0.65	[0.63,0.66]	[0.54,1.03]	[0.41,2.74]	2.32	0.66
	ampl.	0.59	[0.56, 0.61]	[0.47,1.03]	[0.42,3.14]	2.72	
ca BU	per.	1.16	[1.14,1.17]	[0.85,2.22]	[0.53,6.45]	5.92	0.82
	ampl.	1.01	[1.00,1.03]	[0.74,1.94]	[0.46,6.54]	6.08	
neg TD	per.	0.189	[0.188,0.190]	[0.179,0.196]	[0.17,0.20]	0.03	0.36
	ampl.	0.74	[0.71, 0.77]	[0.54,1.22]	[0.38,3.43]	3.05	
neg BU	per.	0.19	0.19	[0.18,0.20]	[0.17,0.22]	0.05	0.30
	ampl.	0.66	[0.65,0.68]	[0.50,0.93]	[0.35,1.84]	1.49	
pos TD	per.	0.69	[0.67,0.71]	[0.53,0.95]	[0.38,2.11]	1.73	0.32
	ampl.	0.53	[0.53, 0.54]	[0.46,0.64]	[0.40,5.13]	4.73	
pos BU	per.	0.68	[0.67, 0.69]	[0.58,0.82]	[0.41,2.40]	1.99	0.42
	ampl.	0.57	[0.56,0.57]	[0.51,0.65]	[0.43,4.91]	4.48	

Different parameter perturbations

Table W: Results for the MWU test comparing the sensitivity distributions of the phenomenological calcium oscillations model [2] and the mammalian circadian oscillations model [1] for different parameter perturbations Δp. All sensitivity distributions are compared to the sensitivity distribution with $\Delta p = 2\%$. Here, # sets denotes the total number of parameter sets sampled in each sensitivity analysis to obtain 250 sensitivity data points. Every distribution is composed of 250 data points, i.e. $n_X = n_Y = 250$. Data correspond to Fig. S15.

Δp	period sensitivity				amplitude sensitivity				
	μ	MWU p	U	z-val	μ	MWU p	U	z-val	# sets
calcium oscillations model (blue)									
$\Delta p = 1\%$	1.161	0.29	-0.56	30347	1.034	0.035	-1.81	28325	7365
$\Delta p = 2\%$	1.183				0.984				6781
$\Delta p = 5\%$	1.146	0.15	-1.05	29550	1.015	0.19	-0.88	29836	7764
$\Delta p = 10\%$	1.133	0.19	-0.88	29830	1.034	0.38	-0.29	30777	9341
$\Delta p = -2\%$	1.179	0.49	-0.029	31203	1.026	0.11	-1.25	29225	7671
circadian oscillations model (red)									
$\Delta p = 1\%$	0.138	0.12	-1.16	29369	0.455	0.18	-0.93	29745	55032
$\Delta p = 2\%$	0.137				0.454				66388
$\Delta p = 5\%$	0.136	0.053	-1.62	28634	0.451	0.46	-0.11	31071	68491
$\Delta p = 10\%$	0.130	$4.4 \cdot 10^{-9}$	-5.75	21960	0.426	0.268	-0.64	30222	74777
$\Delta p = -2\%$	0.141	0.003	-2.75	26814	0.476	0.039	-1.76	28403	65630

References

[1] Becker-Weimann S, Wolf J, Herzel H, Kramer A (2004) Modeling feedback loops of the mammalian circadian oscillator. Biophys J 87: 3023-34.

[2] Goldbeter A, Dupont G, Berridge MJ (1990) Minimal model for signal-induced Ca2+ oscillations and for their frequency encoding through protein phosphorylation. Proc Natl Acad Sci U S A 87: 1461-5.

[3] Goldbeter A (1995) A model for circadian oscillations in the Drosophila period protein (PER). Proc Biol Sci 261: 319-24.

[4] Locke JCW, Southern MM, Kozma-Bognar L, Hibberd V, Brown PE, et al. (2005) Extension of a genetic network model by iterative experimentation and mathematical analysis. Mol Sys Biol 1.

[5] Sneyd J, Tsaneva-Atanasova K, Yule DI, Thompson JL, Shuttleworth TJ (2004) Control of calcium oscillations by membrane fluxes. Proc Natl Acad Sci U S A 101: 1392-6.

[6] De Young GW, Keizer J (1992) A single-pool inositol 1,4,5-trisphosphate-receptor-based model for agonist-stimulated oscillations in Ca2+ concentration. Proc Natl Acad Sci U S A 89: 9895-9.

[7] Elowitz MB, Leibler S (2000) A synthetic oscillatory network of transcriptional regulators. Nature 403: 335-8.

[8] Kholodenko BN (2000) Negative feedback and ultrasensitivity can bring about oscillations in the mitogen-activated protein kinase cascades. Eur J Biochem 267: 1583-8.

[9] Sel'kov EE (1968) Self-oscillations in glycolysis. 1. A simple kinetic model. Eur J Biochem 4: 79-86.

[10] Wolf J, Heinrich R (1997) Dynamics of two-component biochemical systems in interacting cells; synchronization and desynchronization of oscillations and multiple steady states. Biosystems 43: 1-24.

[11] Tyson JJ (1991) Modeling the cell division cycle: cdc2 and cyclin interactions. Proc Natl Acad Sci U S A 88: 7328-32.

[12] FitzHugh R (1961) Impulses and physiological states in theoretical models of nerve membrane. Biophys J 1: 445-66.

[13] Winfree AT (1980) The Geometry of Biological Time, volume 8 of *Biomathematics*. New York: Springer-Verlag.
