# Supplementary material for: Feedback, Mass Conservation and Reaction Kinetics Impact the Robustness of Cellular Oscillations
Source: PLoS Comput Biol. 2016 Dec 27;12(12):e1005298. doi: 10.1371/journal.pcbi.1005298 (PMC5226835; doi:10.1371/journal.pcbi.1005298)
Supplement: S1 Supplementary Information — The descriptions and equations for the three circadian models, the three calcium models, the chain models, the repressilator, the models for MAPK, glycolysis and the cell cycle, as well as the FitzHugh-Nagumo model and the λ-ω system are given. (PDF) [file pcbi.1005298.s017.pdf]

S1 Supplementary Information, Baum et al.: Model descriptions and equations

Mammalian circadian rhythm model

The ODE system describes the circadian rhythm of mammals [1]. Therein, the heterodimer formed by PER2 and CRY (but not the separate entities) and the protein BMAL1 are considered. The nuclear PER2/CRY complex inhibits transcription of the *per2* and/or *cry* genes and positively regulates the transcription of the *bmal1* gene. BMAL1 protein is produced in the cytosol, reversibly transported to the nucleus and reversibly activated. The activated BMAL1 protein enhances *per2/cry* mRNA production. Cytosolic PER2/CRY complex is produced from the *per2/cry* mRNA and can reversibly enter the nucleus. Overall, the model comprises 7 different species, 17 rate coefficients, 4 nl-parameters and 3 cooperativity parameters.

$$
\begin{aligned}
\frac{\mathrm{d}S_1}{\mathrm{d}t} &= k_1 \cdot (S_7 + kn_1)/(kn_2 \cdot (1 + (S_3/kn_3)^{n_1}) + S_7 + kn_1) - k_2 \cdot S_1 \\
\frac{\mathrm{d}S_2}{\mathrm{d}t} &= k_3 \cdot S_1^{n_2} - k_4 \cdot S_2 - k_5 \cdot S_2 + k_6 \cdot S_3 \\
\frac{\mathrm{d}S_3}{\mathrm{d}t} &= k_5 \cdot S_2 - k_6 \cdot S_3 - k_7 \cdot S_3 \\
\frac{\mathrm{d}S_4}{\mathrm{d}t} &= k_8 \cdot S_3^{n_3}/(kn_4^{n_3} + S_3^{n_3}) - k_9 \cdot S_4 \\
\frac{\mathrm{d}S_5}{\mathrm{d}t} &= k_{10} \cdot S_4 - k_{11} \cdot S_5 - k_{12} \cdot S_5 + k_{13} \cdot S_6 \\
\frac{\mathrm{d}S_6}{\mathrm{d}t} &= k_{12} \cdot S_5 - k_{13} \cdot S_6 - k_{14} \cdot S_6 - k_{15} \cdot S_6 + k_{16} \cdot S_7 \\
\frac{\mathrm{d}S_7}{\mathrm{d}t} &= k_{15} \cdot S_6 - k_{16} \cdot S_7 - k_{17} \cdot S_7
\end{aligned}
$$

In the table below, the notation we used is given together with the notation used in the original publication [1], and we provide the reference parameter set published together with the model.

S_1	$y1$		k_5	k_{2t}	$0.24\ h^{-1}$	k_{16}	k_{7a}	$0.003\ h^{-1}$
S_2	$y2$		k_6	k_{3t}	$0.02\ h^{-1}$	k_{17}	k_{7d}	$0.09\ h^{-1}$
S_3	$y3$		k_7	k_{3d}	$0.12\ h^{-1}$	kn_1	c	$0.01\ nM$
S_4	$y4$		k_8	ν_{4b}	$3.6\ nMh^{-1}$	kn_2	k_{1b}	$1\ nM$
S_5	$y5$		k_9	k_{4d}	$0.75\ h^{-1}$	kn_3	k_{1i}	$0.56\ nM$
S_6	$y6$		k_{10}	k_{5b}	$0.24\ h^{-1}$	kn_4	k_{4b}	$2.16\ nM$
S_7	$y7$		k_{11}	k_{5d}	$0.06\ h^{-1}$	n_1	p	8
k_1	ν_{1b}	$9\ nMh^{-1}$	k_{12}	k_{5t}	$0.45\ h^{-1}$	n_2	q	2
k_2	k_{1d}	$0.12\ h^{-1}$	k_{13}	k_{6t}	$0.06\ h^{-1}$	n_3	r	3
k_3	k_{2b}	$0.3\ nM^{-1}h^{-1}$	k_{14}	k_{6d}	$0.12\ h^{-1}$			
k_4	k_{2d}	$0.05\ h^{-1}$	k_{15}	k_{6a}	$0.09\ h^{-1}$			

Drosophila melanogaster circadian rhythm model

The ODE system describes the circadian rhythm of the fruit fly (*Drosophila melanogaster*) [2]. This model considers the *per* mRNA and the PER protein. PER protein is reversibly phosphorylated twice and reversibly transported into the nucleus where it negatively feeds back on the transcription of the *per* mRNA. The model contains 5 different species, 10 rate coefficients, 7 nl-parameters and 1 cooperativity parameter.

$$
\begin{aligned}
\frac{\mathrm{d}S_1}{\mathrm{d}t} &= k_1 \cdot kn_1^{n_1}/(kn_1^{n_1} + S_5^{n_1}) - k_2 \cdot S_1/(kn_2 + S_1) \\
\frac{\mathrm{d}S_2}{\mathrm{d}t} &= k_3 \cdot S_1 - k_4 \cdot S_2/(kn_3 + S_2) + k_5 \cdot S_3/(kn_4 + S_3) \\
\frac{\mathrm{d}S_3}{\mathrm{d}t} &= k_4 \cdot S_2/(kn_3 + S_2) - k_5 \cdot S_3/(kn_4 + S_3) - k_6 \cdot S_3/(kn_5 + S_3) \\
&\quad + k_7 \cdot S_4/(kn_6 + S_4) \\
\frac{\mathrm{d}S_4}{\mathrm{d}t} &= k_6 \cdot S_3/(kn_5 + S_3) - k_7 \cdot S_4/(kn_6 + S_4) - k_8 \cdot S_4 + k_9 \cdot S_5 \\
&\quad - k_{10} \cdot S_4/(kn_7 + S_4) \\
\frac{\mathrm{d}S_5}{\mathrm{d}t} &= k_8 \cdot S_4 - k_9 \cdot S_5
\end{aligned}
$$

In the table below, the notation we used is given together with the notation used in the original publication [2], and we provide the reference parameter set published together with the model.

S_1	M	$0.1\ \mu M$	k_4	V_1	$3.2\ \mu M h^{-1}$	kn_2	K_m	$0.5\ \mu M$
S_2	P_0	$0.25\ \mu M$	k_5	V_2	$1.58\ \mu M h^{-1}$	kn_3	K_1	$2\ \mu M$
S_3	P_1	$0.25\ \mu M$	k_6	V_3	$5\ \mu M h^{-1}$	kn_4	K_2	$2\ \mu M$
S_4	P_2	$0.25\ \mu M$	k_7	V_4	$2.5\ \mu M h^{-1}$	kn_5	K_3	$2\ \mu M$
S_5	P_N	$0.25\ \mu M$	k_8	k_1	$1.9\ h^{-1}$	kn_6	K_4	$2\ \mu M$
k_1	ν_s	$0.76\ \mu M h^{-1}$	k_9	k_2	$1.3\ h^{-1}$	kn_7	K_d	$0.2\ \mu M$
k_2	ν_m	$0.65\ \mu M h^{-1}$	k_{10}	ν_d	$0.95\ \mu M h^{-1}$	n_1	n	4
k_3	k_s	$0.38\ h^{-1}$	kn_1	K_I	1 μM			

Arabidopsis thaliana circadian rhythm model

This model encodes the circadian rhythm of arabidopsis (mouse-ear cress, *Arabidopsis thaliana*) and is published as model2, the interlocked feedback model, in [3]. Therein, light enters as explicit parameter Θ. Here, the model is investigated for constant light or darkness condition. In the case of constant light, species 13 ($c_P^{(n)}$) of the original model is only degraded, it gets eventually zero for all possible parameter sets, and thus does not enter into any of the other equations after a certain integration time. In the case of constant darkness, the concentration of species 13 is completely decoupled from the rest of the ODE system. Therefore, the model is restricted to the 12 other species. The light term (being constant in time) is not explicitly included.

The proteins LHY (late elongated hypocotyl), TOC1 (timing of cab 1) as well as two unknown proteins X and Y, each with their corresponding mRNA and their cytosolic and nuclear concentrations are modeled. For all four proteins, the according mRNA is produced, translated into protein in the cytosol which is then reversibly transported into the nucleus. The mRNA as well as the nuclear and cytosolic proteins are subject to degradations. The mRNA, cytosolic and nuclear proteins of LHY, TOC1 and X are connected via a negative feedback loop in which nuclear LHY protein acts negatively

on *toc1* mRNA, and nuclear TOC1 protein or nuclear X protein act positively on *x* mRNA or *lhy* mRNA, respectively. The *y* mRNA production is inhibited by both nuclear LHY protein and nuclear TOC1 protein. Nuclear Y protein acts positively on *toc1* mRNA production. The model contains 12 species, 28 rate coefficients, 18 nl-parameters and 6 cooperativity parameters.

$$\frac{dS_1}{dt} = k_1 \cdot S_9^{n_1}/({kn_1}^{n_1} + S_9^{n_1}) - k_2 \cdot S_1/(S_1 + kn_2)$$
$$\frac{dS_2}{dt} = k_3 \cdot S_1 - k_4 \cdot S_2 + k_5 \cdot S_3 - k_6 \cdot S_2/(S_2 + kn_3)$$
$$\frac{dS_3}{dt} = k_4 \cdot S_2 - k_5 \cdot S_3 - k_7 \cdot S_3/(S_3 + kn_4)$$
$$\frac{dS_4}{dt} = k_8 \cdot S_{12}^{n_2}/({kn_5}^{n_2} + S_{12}^{n_2}) \cdot {kn_6}^{n_3}/({kn_6}^{n_3} + S_3^{n_3}) - k_9 \cdot S_4/(S_4 + kn_7)$$
$$\frac{dS_5}{dt} = k_{10} \cdot S_4 - k_{11} \cdot S_5 + k_{12} \cdot S_6 - k_{13} \cdot S_5/(S_5 + kn_8)$$
$$\frac{dS_6}{dt} = k_{11} \cdot S_5 - k_{12} \cdot S_6 - k_{14} \cdot S_6/(S_6 + kn_9)$$
$$\frac{dS_7}{dt} = k_{15} \cdot S_6^{n_4}/({kn_{10}}^{n_4} + S_6^{n_4}) - k_{16} \cdot S_7/(S_7 + kn_{11})$$
$$\frac{dS_8}{dt} = k_{17} \cdot S_7 - k_{18} \cdot S_8 + k_{19} \cdot S_9 - k_{20} \cdot S_8/(S_8 + kn_{12})$$
$$\frac{dS_9}{dt} = k_{18} \cdot S_8 - k_{19} \cdot S_9 - k_{21} \cdot S_9/(S_9 + kn_{13})$$
$$\frac{dS_{10}}{dt} = k_{22} \cdot {kn_{14}}^{n_5}/({kn_{14}}^{n_5} + S_6^{n_5}) \cdot {kn_{15}}^{n_6}/({kn_{15}}^{n_6} + S_3^{n_6}) - k_{23} \cdot S_{10}/(S_{10} + kn_{16})$$
$$\frac{dS_{11}}{dt} = k_{24} \cdot S_{10} - k_{25} \cdot S_{11} + k_{26} \cdot S_{12} - k_{27} \cdot S_{11}/(S_{11} + kn_{17})$$
$$\frac{dS_{12}}{dt} = k_{25} \cdot S_{11} - k_{26} \cdot S_{12} - k_{28} \cdot S_{12}/(S_{12} + kn_{18})$$

In the table below, our notation and that of the original publication [3], and the reference parameter set are given (units: nMh^{-1} or h^{-1} for rate coefficients, nM for nl-parameters, no unit for cooperativity parameters). Reactions which in the original model include the light term Θ are here merged with the according basal reactions. Each of these reaction pairs gives rise to only one rate coefficient (k_{13}, k_{14}, k_{22}). The value for each species gives the calculated concentration (in nM) at the unstable steady state for $\Theta = const = 0$ (constant dark).

S_1	$c_L^{(m)}$	0.1960	k_{10}	p_2	4.3240	kn_3	k_2	1.5644
S_2	$c_L^{(c)}$	0.0056	k_{11}	r_3	0.3166	kn_4	k_3	1.2765
S_3	$c_L^{(n)}$	0.0317	k_{12}	r_4	2.1509	kn_5	g_2	0.0368
S_4	$c_T^{(m)}$	1.1746	k_{13}	$(1-\Theta(t))m_5+m_6$	0.0013, 3.1741	kn_6	g_3	0.2658
S_5	$c_T^{(c)}$	12.1230	k_{14}	$(1-\Theta(t))m_7+m_8$	0.0492, 4.0424	kn_7	k_4	2.5734
S_6	$c_T^{(n)}$	0.6269	k_{15}	n_3	0.2431	kn_8	k_5	2.7454
S_7	$c_X^{(m)}$	0.0886	k_{16}	m_9	10.1132	kn_9	k_6	0.4033
S_8	$c_X^{(c)}$	1.0209	k_{17}	p_3	2.1470	kn_{10}	g_4	0.5388
S_9	$c_X^{(n)}$	0.3025	k_{18}	r_5	1.0352	kn_{11}	k_7	6.5585
S_{10}	$c_Y^{(m)}$	0.0416	k_{19}	r_6	3.3017	kn_{12}	k_8	0.6632
S_{11}	$c_Y^{(c)}$	0.0071	k_{20}	m_{10}	0.2179	kn_{13}	k_9	17.1111
S_{12}	$c_Y^{(n)}$	0.0295	k_{21}	m_{11}	3.3442	kn_{14}	g_5	1.1780
k_1	n_1	5.1694	k_{22}	$\Theta(t)n_4+n_5$	0.0857, 0.1649	kn_{15}	g_6	0.0645
k_2	m_1	1.5283	k_{23}	m_{12}	4.2970	kn_{16}	k_{10}	1.7303
k_3	p_1	0.8295	k_{24}	p_4	0.2485	kn_{17}	k_{11}	1.8258
k_4	r_1	16.8363	k_{25}	r_7	2.2123	kn_{18}	k_{12}	1.8066
k_5	r_2	0.1687	k_{26}	r_8	0.2002	n_1	a	3.3064
k_6	m_2	20.4400	k_{27}	m_{13}	0.1347	n_2	b	1.0258
k_7	m_3	3.6888	k_{28}	m_{14}	0.6114	n_3	c	1.0258
k_8	n_2	3.0087	kn_1	g_1	0.8767	n_4	d	1.4422
k_9	m_4	3.8231	kn_2	k_1	1.8170	n_5	e	3.6064
						n_6	f	1.0237

Phenomenological calcium oscillations model

The model [4] is a phenomenological model of cellular calcium oscillations. The calcium concentrations in the cytosol and in the endoplasmic reticulum (ER) are considered as variables. Cytosolic calcium is constantly provided (by two constant rates, one of which depends on a saturation function β by which the effect of IP_3 is included) and linearly removed from the cell. One rate represents the pumping of

calcium from the cytosol to the ER, it depends on a Hill-function of cytosolic calcium. Two processes represent calcium release from the ER to the cytosol: one linear basal rate (‘leaky transport’) and one rate depending on both cytosolic (‘activation’ part) and endoplasmic (‘release’ part) calcium in a Hill-type manner. The model comprises 2 species, 6 rate coefficients, 3 nl-parameters and 3 cooperativity parameters. The equations for the reactions or flows constituting the model are given by

$$\begin{aligned}
\nu_1 &= k_1 \\
\nu_2 &= k_2 \\
\nu_3 &= k_3 \cdot \frac{S_1^{n_1}}{{kn_1}^{n_1} + S_1^{n_1}} \\
\nu_4 &= k_4 \cdot \frac{S_2^{n_2} \cdot S_1^{n_3}}{({kn_2}^{n_2} + S_2^{n_2}) \cdot ({kn_3}^{n_3} + S_1^{n_3})} \\
\nu_5 &= k_5 \cdot S_2 \\
\nu_6 &= k_6 \cdot S_1.
\end{aligned}$$

The model is composed from these six reactions by

$$\begin{aligned}
\frac{\mathrm{d}S_1}{\mathrm{d}t} &= \nu_1 + \nu_2 - \nu_3 + \nu_4 + \nu_5 - \nu_6 \\
\frac{\mathrm{d}S_2}{\mathrm{d}t} &= \nu_3 - \nu_4 - \nu_5.
\end{aligned}$$

In the table below, the notation we used is given together with the notation used in the original publication [4], and we provide the reference parameter set published together with the model. The saturation β is assumed to take a value of 0.4 for the estimation of the sensitivities at the reference parameter set. Species concentrations give steady state concentrations (of the unstable steady state) for the given reference parameter set.

S_1	Z	$0.3920\ \mu M$	k_4	V_{M3}	$500\ \mu M s^{-1}$	kn_3	K_A	$0.9\ \mu M$
S_2	Y	$1.6456\ \mu M$	k_5	k_f	$1\ s^{-1}$	n_1	n	2
k_1	ν_0	$1\ \mu M s^{-1}$	k_6	k	$10\ s^{-1}$	n_2	m	2
k_2	$\nu_1 \cdot \beta$	$7.3\ \mu M s^{-1}$, $(0.3, 0.77)$	kn_1	K_2	$1\ \mu M$	n_3	p	4
k_3	V_{M2}	$65\ \mu M s^{-1}$	kn_2	K_R	$2\ \mu M$			

Closed cell calcium oscillations model

The model proposed in [5] focuses on intracellular calcium oscillations arising from the change in loci of calcium from the ER to the cytosol through IP_3-receptor channels. Influx and efflux to or from the cell are neglected, i.e. the sum of cytosolic and endoplasmic calcium is constant. We use the full model in which IP_3 enters as a constant and is represented as nl-parameter. Cytosolic calcium is modeled as variable. The IP_3-receptor/channel is assumed to have three subunits with three binding sites each, one for IP_3 and two for calcium (one of which for activation, one for inhibition). The 8 possible states of the IP_3-receptor channel are included as variables. Since the values of receptor state variables have to be interpreted as probabilities, they sum up to one ($\sum_{i=2}^{9} S_i = 1$). Influx of calcium into the cytosol through the IP_3-receptor channel is only possible if IP_3 and a calcium molecule is bound at the activation site (S_6). The authors assumed that the probability of the calcium channel to be open is proportional to $S_6^{n_1}$ with $n_1 = 3$. There is one calcium flux from ER to cytosol depending only on the concentration difference between the two compartments ('leak flux'), and one flux through the IP_3-receptor/channels depending additionally on $S_6^{n_1}$. Calcium flux from cytosol to ER is assumed to follow Hill-type kinetics and represents the function of ATP-dependent pumps. The model consists of 9 variables, 27 rate coefficients, 4 nl-parameters and 2 cooperativity parameters.

$$\frac{\mathrm{d}S_1}{\mathrm{d}t} = k_1 \cdot S_6^{n_1} \cdot (kn_3 - S_1 - kn_4 \cdot S_1) + k_2 \cdot (kn_3 - S_1 - kn_4 \cdot S_1) - \frac{k_3 \cdot S_1^{n_2}}{S_1^{n_2} + kn_1^{n_2}}$$
$$\frac{\mathrm{d}S_2}{\mathrm{d}t} = k_5 \cdot S_3 + k_{21} \cdot S_4 + k_{17} \cdot S_5 - (k_4 \cdot kn_2 + k_{16} \cdot S_1 + k_{20} \cdot S_1) \cdot S_2$$
$$\frac{\mathrm{d}S_3}{\mathrm{d}t} = k_4 \cdot kn_2 \cdot S_2 + k_{23} \cdot S_6 + k_9 \cdot S_7 - (k_8 \cdot S_1 + k_5 + k_{22} \cdot S_1) \cdot S_3$$
$$\frac{\mathrm{d}S_4}{\mathrm{d}t} = k_{20} \cdot S_1 \cdot S_2 + k_7 \cdot S_6 + k_{19} \cdot S_8 - (k_{18} \cdot S_1 + k_6 \cdot kn_2 + k_{21}) \cdot S_4$$
$$\frac{\mathrm{d}S_5}{\mathrm{d}t} = k_{16} \cdot S_1 \cdot S_2 + k_{13} \cdot S_7 + k_{25} \cdot S_8 - (k_{12} \cdot kn_2 + k_{17} + k_{24} \cdot S_1) \cdot S_5$$
$$\frac{\mathrm{d}S_6}{\mathrm{d}t} = k_{22} \cdot S_1 \cdot S_3 + k_6 \cdot kn_2 \cdot S_4 + k_{11} \cdot S_9 - (k_{10} \cdot S_1 + k_7 + k_{23}) \cdot S_6$$
$$\frac{\mathrm{d}S_7}{\mathrm{d}t} = k_8 \cdot S_1 \cdot S_3 + k_{12} \cdot kn_2 \cdot S_5 + k_{27} \cdot S_9 - (k_9 + k_{13} + k_{26} \cdot S_1) \cdot S_7$$

$$\frac{dS_8}{dt} = k_{18} \cdot S_1 \cdot S_4 + k_{24} \cdot S_1 \cdot S_5 + k_{15} \cdot S_9 - (k_{14} \cdot kn_2 + k_{19} + k_{25}) \cdot S_8$$
$$\frac{dS_9}{dt} = k_{10} \cdot S_1 \cdot S_6 + k_{26} \cdot S_1 \cdot S_7 + k_{14} \cdot kn_2 \cdot S_8 - (k_{15} + k_{11} + k_{27}) \cdot S_9$$

In the table below, the notation we used is given together with the notation used in the original publication [5], and we provide the reference parameter set published together with the model. For IP_3, a concentration of $0.5\ \mu M$ is used for determining the sensitivity at the reference parameter set.

S_1	$[Ca_i^{2+}]$		k_6	a_1	$400\ \mu M^{-1}s^{-1}$	k_{20}	a_5	$20\ \mu M^{-1}s^{-1}$
S_2	S_{000}		k_7	b_1	$52\ s^{-1}$	k_{21}	b_5	$1.6468\ s^{-1}$
S_3	S_{100}		k_8	a_2	$0.2\ \mu M^{-1}s^{-1}$	k_{22}	a_5	$20\ \mu M^{-1}s^{-1}$
S_4	S_{010}		k_9	b_2	$0.2098\ s^{-1}$	k_{23}	b_5	$1.6468\ s^{-1}$
S_5	S_{001}		k_{10}	a_2	$0.2\ \mu M^{-1}s^{-1}$	k_{24}	a_5	$20\ \mu M^{-1}s^{-1}$
S_6	S_{110}		k_{11}	b_2	$0.2098\ s^{-1}$	k_{25}	b_5	$1.6468\ s^{-1}$
S_7	S_{101}		k_{12}	a_3	$400\ \mu M^{-1}s^{-1}$	k_{26}	a_5	$20\ \mu M^{-1}s^{-1}$
S_8	S_{011}		k_{13}	b_3	$377.36\ s^{-1}$	k_{27}	b_5	$1.6468\ s^{-1}$
S_9	S_{111}		k_{14}	a_3	$400\ \mu M^{-1}s^{-1}$	kn_1	k_3	$0.1\ \mu M$
k_1	ν_1	$6\ s^{-1}$	k_{15}	b_3	$377.36\ s^{-1}$	kn_2	$[IP3]$	$(0.37\mu M, 0.62\mu M)$
k_2	ν_2	$0.11\ s^{-1}$	k_{16}	a_4	$0.2\ \mu M^{-1}s^{-1}$	kn_3	c_0	$2.0\ \mu M$
k_3	ν_3	$0.9\ \mu M^{-1}s^{-1}$	k_{17}	b_4	$0.0289\ s^{-1}$	kn_4	c_1	0.185
k_4	a_1	$400\ \mu M^{-1}s^{-1}$	k_{18}	a_4	$0.2\ \mu M^{-1}s^{-1}$	n_1		3
k_5	b_1	$52\ s^{-1}$	k_{19}	b_4	$0.0289\ s^{-1}$	n_2		2

Open cell calcium oscillations model

The calcium oscillations model considers the cytosolic calcium concentration as well as its total concentration in the cell [6]. It is an open cell model which means that it allows for an influx of calcium into the cell (at two constant rates, one of which depends on the constant concentration of IP_3) and an efflux from the cell (modeled by a Hill-kinetics term depending on the cytosolic calcium concentration). Six IP_3-receptor states are considered whose probabilities add up to one. Resolving this conservation condition, five independent receptor state probabilities are incorporated

as variables into the model. The receptor allows for calcium flux to the cytosol (from the ER or comparable compartments) in only two states, S_4 and S_5. This calcium flux is assumed to be proportional to $(0.1 \cdot S_4 + 0.9 \cdot S_5)^{n_1}$. The model contains 7 variables, 24 rate coefficients, 7 nl-parameters and 3 cooperativity parameters.

$$
\begin{aligned}
\frac{\mathrm{d}S_1}{\mathrm{d}t} &= k_1 \cdot (0.1 \cdot S_4 + 0.9 \cdot S_5)^{n_1} \cdot (\frac{S_2}{kn_7} - (1 + \frac{1}{kn_7}) \cdot S_1) \\
&+ k_2 \cdot (\frac{S_2}{kn_7} - (1 + \frac{1}{kn_7}) \cdot S_1) - k_3 \cdot \frac{S_1}{kn_1 + S_1} \cdot \frac{kn_7}{S_2 - S_1} \\
&+ k_4 + k_5 \cdot kn_3 - k_6 \cdot \frac{S_1^{n_2}}{kn_2^{n_2} + S_1^{n_2}} \\
\frac{\mathrm{d}S_2}{\mathrm{d}t} &= k_4 + k_5 \cdot kn_3 - k_6 \cdot \frac{S_1^{n_3}}{kn_2^{n_3} + S_1^{n_3}} \\
\frac{\mathrm{d}S_3}{\mathrm{d}t} &= k_7 \cdot \frac{kn_6}{kn_6 + S_1} \cdot S_4 + k_8 \cdot \frac{kn_6 \cdot S_1}{kn_6 + S_1} \cdot S_4 - k_9 \cdot \frac{kn_4 \cdot kn_5 \cdot kn_3 \cdot S_3}{kn_4 \cdot kn_5 + S_1 \cdot (kn_4 + kn_5)} \\
&- k_{10} \cdot \frac{kn_4 \cdot S_1 \cdot S_3 \cdot kn_3}{kn_4 \cdot kn_5 + S_1 \cdot (kn_4 + kn_5)} + k_{11} \cdot S_6 + k_{12} \cdot S_6 \\
&- k_{13} \cdot \frac{kn_4 \cdot kn_5 \cdot S_1 \cdot S_3}{kn_4 \cdot kn_5 + S_1 \cdot (kn_4 + kn_5)} - k_{14} \cdot \frac{kn_5 \cdot S_1 \cdot S_3}{kn_4 \cdot kn_5 + S_1 \cdot (kn_4 + kn_5)} \\
\frac{\mathrm{d}S_4}{\mathrm{d}t} &= k_9 \cdot \frac{kn_4 \cdot kn_5 \cdot kn_3 \cdot S_3}{kn_4 \cdot kn_5 + S_1 \cdot (kn_4 + kn_5)} + k_{10} \cdot \frac{kn_4 \cdot S_1 \cdot S_3 \cdot kn_3}{kn_4 \cdot kn_5 + S_1 \cdot (kn_4 + kn_5)} \\
&- k_7 \cdot \frac{kn_6}{kn_6 + S_1} \cdot S_4 - k_8 \cdot \frac{kn_6 \cdot S_1}{kn_6 + S_1} \cdot S_4 - k_{15} \cdot \frac{kn_6 \cdot S_1 \cdot S_4}{kn_6 + S_1} - k_{16} \cdot \frac{S_1 \cdot S_4}{kn_6 + S_1} \\
&- k_{17} \cdot \frac{kn_6 \cdot S_4}{kn_6 + S_1} + k_{18} \cdot \frac{kn_4 \cdot S_5}{kn_4 + S_1} + k_{19} \cdot \frac{kn_4 \cdot S_5}{kn_4 + S_1} \\
&+ k_{20} \cdot (1 - S_3 - S_4 - S_5 - S_6 - S_7) \\
\frac{\mathrm{d}S_5}{\mathrm{d}t} &= k_{15} \cdot \frac{kn_6 \cdot S_1 \cdot S_4}{kn_6 + S_1} + k_{16} \cdot \frac{S_1 \cdot S_4}{kn_6 + S_1} - k_{18} \cdot \frac{kn_4 \cdot S_5}{kn_4 + S_1} - k_{19} \cdot \frac{kn_4 \cdot S_5}{kn_4 + S_1} \\
&- k_{21} \cdot \frac{kn_4 \cdot S_1 \cdot S_5}{kn_4 + S_1} - k_{22} \cdot \frac{S_1 \cdot S_5}{kn_4 + S_1} + k_{23} \cdot S_7 + k_{24} \cdot S_7 \\
\frac{\mathrm{d}S_6}{\mathrm{d}t} &= k_{13} \cdot \frac{kn_4 \cdot kn_5 \cdot S_1 \cdot S_3}{kn_4 \cdot kn_5 + S_1 \cdot (kn_4 + kn_5)} + k_{14} \cdot \frac{kn_5 \cdot S_1 \cdot S_3}{kn_4 \cdot kn_5 + S_1 \cdot (kn_4 + kn_5)} \\
&- k_{11} \cdot S_6 - k_{12} \cdot S_6 \\
\frac{\mathrm{d}S_7}{\mathrm{d}t} &= k_{21} \cdot \frac{kn_4 \cdot S_1 \cdot S_5}{kn_4 + S_1} + k_{22} \cdot \frac{S_1 \cdot S_5}{kn_4 + S_1} - k_{23} \cdot S_7 - k_{24} \cdot S_7
\end{aligned}
$$

In the table below, the notation we used is given together with the notation used

in the original publication [6], and we provide the reference parameter set published together with the model.

S_1	C		k_8	l_{-4}	$2.5\ \mu M^{-1}s^{-1}$	k_{22}	l_2	$1.7\ s^{-1}$
S_2	C_t		k_9	k_2	$37.4\ \mu M^{-1}s^{-1}$	k_{23}	k_{-1}	$0.04\ s^{-1}$
S_3	R		k_{10}	l_4	$1.7\ \mu M^{-1}s^{-1}$	k_{24}	l_{-2}	$0.8\ s^{-1}$
S_4	O		k_{11}	k_{-1}	$0.04\ s^{-1}$	kn_1	K_S	$0.18\ \mu M$
S_5	A		k_{12}	l_{-2}	$0.8\ s^{-1}$	kn_2	K_p	$0.42\ \mu M$
S_6	I_1		k_{13}	k_1	$0.64\ s^{-1}$	kn_3	p	10
S_7	I_2		k_{14}	l_2	$1.7\ s^{-1}$	kn_4	L_1	$0.12\ \mu M$
k_1	k_f	$0.96\ s^{-1}$	k_{15}	k_4	$4\ \mu M^{-1}s^{-1}$	kn_5	L_3	$0.025\ \mu M$
k_2	g_1	$0.002\ s^{-1}$	k_{16}	l_6	$4707\ s^{-1}$	kn_6	L_5	$54.7\ \mu M$
k_3	V_s	$120\ \mu M^2 s^{-1}$	k_{17}	k_3	$0.11\ \mu M s^{-1}$	kn_7	$1/\gamma$	$1/5.4$
k_4	$\delta \cdot \alpha_1$	$0.01 \cdot 0.03\ \mu M s^{-1}$	k_{18}	k_{-4}	$0.54\ s^{-1}$	n_1	n	4
k_5	$\delta \cdot \alpha_2$	$0.01 \cdot 0.2\ s^{-1}$,	k_{19}	l_{-6}	$11.4\ s^{-1}$	n_2	m	2
k_6	$\delta \cdot V_p$	$0.01 \cdot 28\ \mu M s^{-1}$	k_{20}	k_{-3}	$29.8\ s^{-1}$			
k_7	k_{-2}	$1.4\ s^{-1}$	k_{21}	k_1	$0.64\ s^{-1}$			

Chain models with mass action kinetics

The chain models have been introduced in [7]. They are prototypes of oscillator models based on a single positive or a single negative feedback. The 4 modeled species ($S_1, \ldots, S_4$) are connected in a chain of irreversible reactions. Each species is degraded. The fourth species is feeding back on the reaction between the first and the second species (ν_2). The feedback term (fb) is therefore depending on the species concentration S_4, as well as on an nl-parameter and on a cooperativity parameter. The models contain 4 variables ($S_1, \ldots, S_4$), 8 rate coefficients ($k_1, \ldots, k_8$),

1 nl-parameter (kn_1) and 1 cooperativity parameter (n_1).

$$\frac{\text{d}S_1}{\text{d}t} = k_1 - k_2 \cdot S_1 \cdot fb - k_3 \cdot S_1$$
$$\frac{\text{d}S_2}{\text{d}t} = k_2 \cdot S_1 \cdot fb - k_4 \cdot S_2 - k_5 \cdot S_2$$
$$\frac{\text{d}S_3}{\text{d}t} = k_4 \cdot S_2 - k_6 \cdot S_3 - k_7 \cdot S_3$$
$$\frac{\text{d}S_4}{\text{d}t} = k_6 \cdot S_3 - k_8 \cdot S_4$$

The feedback term fb enters only in reaction $\nu_2 = k_2 \cdot S_1 \cdot fb$ and is given by

$$fb = \begin{cases} 1/(1 + (\frac{S_4}{kn_1})^{n_1}) & \text{for the negative feedback} \\ 1 + \left(\frac{S_4}{kn_1}\right)^{n_1} & \text{for the positive feedback.} \end{cases}$$

If not stated otherwise, the cooperativity parameter is set to $n_1 = 9$ or $n_1 = 2$ for the negative feedback or positive feedback model, respectively.

Chain models with Michaelis-Menten kinetics in the degradation reactions

The degradation reactions $\nu_3, \nu_5, \nu_7, \nu_8$ of the chain models are modeled by Michaelis-Menten kinetics. The feedback terms and the cooperativity parameter are exactly as in the chain models with mass action kinetics. The chain models with Michaelis-Menten kinetics in the degradation reactions comprise 4 species ($S_1, \ldots, S_4$), 8 rate coefficients ($k_1, k_2, V_3, k_4, V_5, k_6, V_7, V_8$), 5 nl-parameters ($kn_1, K_3, K_5, K_7, K_8$) and 1

cooperativity parameter (n_1).

$$\frac{\mathrm{d}S_1}{\mathrm{d}t} = k_1 - k_2 \cdot S_1 \cdot fb - V_3 \cdot \frac{S_1}{S_1 + K_3}$$
$$\frac{\mathrm{d}S_2}{\mathrm{d}t} = k_2 \cdot S_1 \cdot fb - k_4 \cdot S_2 - V_5 \cdot \frac{S_2}{S_2 + K_5}$$
$$\frac{\mathrm{d}S_3}{\mathrm{d}t} = k_4 \cdot S_2 - k_6 \cdot S_3 - V_7 \cdot \frac{S_3}{S_3 + K_7}$$
$$\frac{\mathrm{d}S_4}{\mathrm{d}t} = k_6 \cdot S_3 - V_8 \cdot \frac{S_4}{S_4 + K_8}$$

Chain models with Michaelis-Menten kinetics in the conversion reactions

The conversion reactions ν_2, ν_4, ν_6 of the chain models are modeled by Michaelis-Menten kinetics. The feedback terms and the cooperativity parameter are exactly as in the chain models with mass action kinetics. The chain models with Michaelis-Menten kinetics in the conversion reactions comprise 4 species ($S_1, \ldots, S_4$), 8 rate coefficients (k_1, V_2, k_3, V_4, k_5, V_6, k_7, k_8), 4 nl-parameters (kn_1, K_2, K_4, K_6) and 1 cooperativity parameter (n_1).

$$\frac{\mathrm{d}S_1}{\mathrm{d}t} = k_1 - V_2 \cdot \frac{S_1}{S_1 + K_2} \cdot fb - k_3 \cdot S_1$$
$$\frac{\mathrm{d}S_2}{\mathrm{d}t} = V_2 \cdot \frac{S_1}{S_1 + K_2} \cdot fb - V_4 \cdot \frac{S_2}{S_2 + K_4} - k_5 \cdot S_2$$
$$\frac{\mathrm{d}S_3}{\mathrm{d}t} = V_4 \cdot \frac{S_2}{S_2 + K_4} - V_6 \cdot \frac{S_3}{S_3 + K_6} - k_7 \cdot S_3$$
$$\frac{\mathrm{d}S_4}{\mathrm{d}t} = V_6 \cdot \frac{S_3}{S_3 + K_6} - k_8 \cdot S_4$$

Chain models with Michaelis-Menten kinetics in all reactions

All reactions of the chain models except for the constant production ν_1 are modeled by Michaelis-Menten kinetics. The feedback terms and the cooperativity parameter are exactly as in the chain models with mass action kinetics. The chain models with Michaelis-Menten kinetics comprise 4 species ($S_1, \ldots, S_4$), 8 rate coefficients

$(k_1, V_2, \ldots, V_8)$, 8 nl-parameters $(kn_1, K_2, \ldots, K_8)$ and 1 cooperativity parameter (n_1).

$$\frac{\mathrm{d}S_1}{\mathrm{d}t} = k_1 - V_2 \cdot \frac{S_1}{S_1 + K_2} \cdot fb - V_3 \cdot \frac{S_1}{S_1 + K_3}$$
$$\frac{\mathrm{d}S_2}{\mathrm{d}t} = V_2 \cdot \frac{S_1}{S_1 + K_2} \cdot fb - V_4 \cdot \frac{S_2}{S_2 + K_4} - V_5 \cdot \frac{S_2}{S_2 + K_5}$$
$$\frac{\mathrm{d}S_3}{\mathrm{d}t} = V_4 \cdot \frac{S_2}{S_2 + K_4} - V_6 \cdot \frac{S_3}{S_3 + K_6} - V_7 \cdot \frac{S_3}{S_3 + K_7}$$
$$\frac{\mathrm{d}S_4}{\mathrm{d}t} = V_6 \cdot \frac{S_3}{S_3 + K_6} - V_8 \cdot \frac{S_4}{S_4 + K}$$

Chain models with different mass conservation properties

Chain models are investigated in which some or all of the conversions (reactions 2, 4, 6) are replaced by regulated productions. The feedback terms and the cooperativity parameter are exactly as in the chain models with mass action kinetics. All models comprise 4 species $(S_1, \ldots, S_4)$, 8 rate coefficients $(k_1, \ldots, k_8)$, 1 nl-parameter (kn_1) and 1 cooperativity parameter (n_1).

For the negative feedback model, the model with regulated productions in reaction 2, 4 and 6 is examined:

$$\frac{\mathrm{d}S_1}{\mathrm{d}t} = k_1 - k_3 \cdot S_1$$
$$\frac{\mathrm{d}S_2}{\mathrm{d}t} = k_2 \cdot S_1 \cdot fb - k_5 \cdot S_2$$
$$\frac{\mathrm{d}S_3}{\mathrm{d}t} = k_4 \cdot S_2 - k_7 \cdot S_3$$
$$\frac{\mathrm{d}S_4}{\mathrm{d}t} = k_6 \cdot S_3 - k_8 \cdot S_4.$$

For the positive feedback model, reaction 2 being a regulated production renders the occurrence of sustained oscillations impossible. Thus, we examine the model

with regulated productions in reactions 4 and 6:

$$\frac{\mathrm{d}S_1}{\mathrm{d}t} = k_1 - k_2 \cdot S_1 \cdot fb - k_3 \cdot S_1$$
$$\frac{\mathrm{d}S_2}{\mathrm{d}t} = k_2 \cdot S_1 \cdot fb - k_5 \cdot S_2$$
$$\frac{\mathrm{d}S_3}{\mathrm{d}t} = k_4 \cdot S_2 - k_7 \cdot S_3$$
$$\frac{\mathrm{d}S_4}{\mathrm{d}t} = k_6 \cdot S_3 - k_8 \cdot S_4.$$

For the chain models with regulated productions and Michaelis-Menten kinetics, the mass action kinetics for reactions $\nu_2, \ldots, \nu_8$ in the two models above are replaced by Michaelis-Menten terms (see chain models with Michaelis-Menten kinetics in all reactions).

Model of the repressilator

The repressilator proposed in [8] is constructed by three gene-protein modules using the genes *lacI* of *E. coli*, *tetR* and *cI* from λ phage. In each module, the mRNA is transcribed and degraded and the protein is translated from the mRNA and also degraded. The proteins repress the transcription of one other mRNA such that a circular regulation is established. The model contains 6 variables, 15 rate coefficients, 3 nl-parameters and 3 cooperativity parameters.

$$\frac{\mathrm{d}S_1}{\mathrm{d}t} = -k_1 \cdot S_1 + k_2 \cdot S_4$$
$$\frac{\mathrm{d}S_2}{\mathrm{d}t} = -k_3 \cdot S_2 + k_4 \cdot S_5$$
$$\frac{\mathrm{d}S_3}{\mathrm{d}t} = -k_5 \cdot S_3 + k_6 \cdot S_6$$
$$\frac{\mathrm{d}S_4}{\mathrm{d}t} = k_7 + k_8 \cdot \frac{kn_1^{n_1}}{kn_1^{n_1} + S_3^{n_1}} - k_9 \cdot S_4$$
$$\frac{\mathrm{d}S_5}{\mathrm{d}t} = k_{10} + k_{11} \cdot \frac{kn_2^{n_2}}{kn_2^{n_2} + S_1^{n_2}} - k_{12} \cdot S_5$$
$$\frac{\mathrm{d}S_6}{\mathrm{d}t} = k_{13} + k_{14} \cdot \frac{kn_3^{n_3}}{kn_3^{n_3} + S_2^{n_3}} - k_{15} \cdot S_6$$

In the table below, the notation we used is given together with the notation used in the original publication [8], and we provide the initial conditions and reference parameter set published together with the model.

S_1	p_{lacI}	0	k_4	β_1	$0.1155\ s^{-1}$	k_{13}	α_0	$5 \cdot 10^{-4}\ s^{-1}$
S_2	p_{tetR}	0	k_5	β	$0.0012\ s^{-1}$	k_{14}	α_1	$0.5\ s^{-1}$
S_3	p_{cI}	0	k_6	β_1	$0.1155\ s^{-1}$	k_{15}	k_1	$0.0058\ s^{-1}$
S_4	m_{lacI}	1	k_7	α_0	$5 \cdot 10^{-4}\ s^{-1}$	kn_1	K_M	40
S_5	m_{tetR}	0	k_8	α_1	$0.5\ s^{-1}$	kn_2	K_M	40
S_6	m_{cI}	0	k_9	k_1	$0.0058\ s^{-1}$	kn_3	K_M	40
k_1	β	$0.0012\ s^{-1}$	k_{10}	α_0	$5 \cdot 10^{-4}\ s^{-1}$	n_1	n	2
k_2	β_1	$0.1155\ s^{-1}$	k_{11}	α_1	$0.5\ s^{-1}$	n_2	n	2
k_3	β	$0.0012\ s^{-1}$	k_{12}	k_1	$0.0058\ s^{-1}$	n_3	n	2

Repressilator with Michaelis-Menten kinetics

The three linear protein productions and all six linear degradations are replaced by Michaelis-Menten terms. The model contains 6 variables, 15 rate coefficients, 12 nl-parameters and 3 cooperativity parameters.

$$\frac{\mathrm{d}S_1}{\mathrm{d}t} = -k_1 \cdot \frac{S_1}{S_1 + kn_1} + k_2 \cdot \frac{S_4}{S_4 + kn_2}$$
$$\frac{\mathrm{d}S_2}{\mathrm{d}t} = -k_3 \cdot \frac{S_2}{S_2 + kn_3} + k_4 \cdot \frac{S_5}{S_5 + kn_4}$$
$$\frac{\mathrm{d}S_3}{\mathrm{d}t} = -k_5 \cdot \frac{S_3}{S_3 + kn_5} + k_6 \cdot \frac{S_6}{S_6 + kn_6}$$
$$\frac{\mathrm{d}S_4}{\mathrm{d}t} = k_7 + k_8 \cdot \frac{kn_7^{n_1}}{kn_7^{n_1} + S_3^{n_1}} - k_9 \cdot \frac{S_4}{S_4 + kn_8}$$
$$\frac{\mathrm{d}S_5}{\mathrm{d}t} = k_{10} + k_{11} \cdot \frac{kn_9^{n_2}}{kn_9^{n_2} + S_1^{n_2}} - k_{12} \cdot \frac{S_5}{S_5 + kn_{10}}$$
$$\frac{\mathrm{d}S_6}{\mathrm{d}t} = k_{13} + k_{14} \cdot \frac{kn_{11}^{n_3}}{kn_{11}^{n_3} + S_2^{n_3}} - k_{15} \cdot \frac{S_6}{S_6 + kn_{12}}$$

MAPK cascade model

The model for the mitogen-activated phosphorylation kinase (MAPK) cascade proposed in [9] captures the first, second and third level of a phosphorylation cascade which occurs for example in epidermal growth factor (EGF) signaling. The kinase on the first level can be reversibly phosphorylated once, the kinases on levels 2 and 3 can be reversibly phosphorylated twice. The phosphorylated kinase of the first level acts positively on the phosphorylations on the second level, the double phosphorylated kinase of the second level acts positively on the phosphorylations on the third level. The double phosphorylated kinase of the third level acts negatively on the phosphorylation on the first level. All phosphorylations are governed by Michaelis-Menten kinetics. The model contains 8 variables, 10 rate coefficients, 11 nl-parameters and 1 cooperativity parameter.

$$
\begin{aligned}
\frac{\mathrm{d}S_1}{\mathrm{d}t} &= -k_1 \cdot \frac{S_1}{kn_2 + S_1} \cdot \frac{kn_1^{n_1}}{kn_1^{n_1} + S_8^{n_1}} + k_2 \cdot \frac{S_2}{S_2 + kn_3} \\
\frac{\mathrm{d}S_2}{\mathrm{d}t} &= k_1 \cdot \frac{S_1}{kn_2 + S_1} \cdot \frac{kn_1^{n_1}}{kn_1^{n_1} + S_8^{n_1}} - k_2 \cdot \frac{S_2}{S_2 + kn_3} \\
\frac{\mathrm{d}S_3}{\mathrm{d}t} &= k_6 \cdot \frac{S_4}{kn_7 + S_4} - k_3 \cdot \frac{S_2 \cdot S_3}{kn_4 + S_3} \\
\frac{\mathrm{d}S_4}{\mathrm{d}t} &= k_3 \cdot \frac{S_2 \cdot S_3}{kn_4 + S_3} - k_6 \cdot \frac{S_4}{kn_7 + S_4} + k_5 \cdot \frac{S_5}{kn_6 + S_5} - k_4 \cdot \frac{S_2 \cdot S_4}{kn_5 + S_4} \\
\frac{\mathrm{d}S_5}{\mathrm{d}t} &= k_4 \cdot \frac{S_2 \cdot S_4}{kn_5 + S_4} - k_5 \cdot \frac{S_5}{kn_6 + S_5} \\
\frac{\mathrm{d}S_6}{\mathrm{d}t} &= k_{10} \cdot \frac{S_7}{kn_{11} + S_7} - k_7 \cdot \frac{S_5 \cdot S_6}{kn_8 + S_6} \\
\frac{\mathrm{d}S_7}{\mathrm{d}t} &= k_7 \cdot \frac{S_5 \cdot S_6}{kn_8 + S_6} - k_{10} \cdot \frac{S_7}{kn_{11} + S_7} - k_8 \cdot \frac{S_5 \cdot S_7}{kn_9 + S_7} + k_9 \cdot \frac{S_8}{kn_{10} + S_8} \\
\frac{\mathrm{d}S_8}{\mathrm{d}t} &= k_8 \cdot \frac{S_5 \cdot S_7}{kn_9 + S_7} - k_9 \cdot \frac{S_8}{kn_{10} + S_8}
\end{aligned}
$$

In the table below, the notation we used is given together with the notation used in the original publication [9], and we provide the initial conditions and reference parameter set published together with the model.

S_1	$MKKK$	$100\ nM$	k_3	k_3	$0.025\ s^{-1}$	kn_3	K_2	$8\ nM$
S_2	$MKKK-P$	0	k_4	k_4	$0.025\ s^{-1}$	kn_4	K_3	$15\ nM$
S_3	MKK	$300\ nM$	k_5	V_5	$0.75\ nM \cdot s^{-1}$	kn_5	K_4	$15\ nM$
S_4	$MKK-P$	0	k_6	V_6	$0.75\ nM \cdot s^{-1}$	kn_6	K_5	$15\ nM$
S_5	$MKK-PP$	0	k_7	k_7	$0.025\ s^{-1}$	kn_7	K_6	$15\ nM$
S_6	$MAPK$	$300\ nM$	k_8	k_8	$0.025\ s^{-1}$	kn_8	K_7	$15\ nM$
S_7	$MAPK-P$	0	k_9	V_9	$0.5\ nM \cdot s^{-1}$	kn_9	K_8	$15\ nM$
S_8	$MAPK-PP$	0	k_{10}	V_{10}	$0.5\ nM \cdot s^{-1}$	kn_{10}	K_9	$15\ nM$
k_1	V_1	$2.5\ nM \cdot s^{-1}$	kn_1	K_I	$9\ nM$	kn_{11}	K_{10}	$15\ nM$
k_2	V_2	$0.25\ nM \cdot s^{-1}$	kn_2	K_1	$10\ nM$	n_1	n	1

MAPK cascade model with mass action kinetics

In all 10 phosphorylations and dephosphorylations the Michaelis-Menten kinetics are replaced by linear mass action kinetics. Note that in order to obtain sustained oscillations, the cooperativity parameter was set to $n_1 = 3$. The model contains 8 variables, 10 rate coefficients, 1 nl-parameters and 1 cooperativity parameter.

$$\frac{\mathrm{d}S_1}{\mathrm{d}t} = -k_1 \cdot S_1 \cdot \frac{kn_1^{n_1}}{kn_1^{n_1} + S_8^{n_1}} + k_2 \cdot S_2$$
$$\frac{\mathrm{d}S_2}{\mathrm{d}t} = k_1 \cdot S_1 \cdot \frac{kn_1^{n_1}}{kn_1^{n_1} + S_8^{n_1}} - k_2 \cdot S_2$$
$$\frac{\mathrm{d}S_3}{\mathrm{d}t} = k_6 \cdot S_4 - k_3 \cdot S_2 \cdot S_3$$
$$\frac{\mathrm{d}S_4}{\mathrm{d}t} = k_3 \cdot S_2 \cdot S_3 - k_6 \cdot S_4 + k_5 \cdot S_5 - k_4 \cdot S_2 \cdot S_4$$
$$\frac{\mathrm{d}S_5}{\mathrm{d}t} = k_4 \cdot S_2 \cdot S_4 - k_5 \cdot S_5$$
$$\frac{\mathrm{d}S_6}{\mathrm{d}t} = k_{10} \cdot S_7 - k_7 \cdot S_5 \cdot S_6$$
$$\frac{\mathrm{d}S_7}{\mathrm{d}t} = k_7 \cdot S_5 \cdot S_6 - k_{10} \cdot S_7 - k_8 \cdot S_5 \cdot S_7 + k_9 \cdot S_8$$
$$\frac{\mathrm{d}S_8}{\mathrm{d}t} = k_8 \cdot S_5 \cdot S_7 - k_9 \cdot S_8$$

Glycolysis model

The model for glycolysis proposed in [10] considers that ATP is supplied by a certain source. The reaction mediated by the enzyme phosphofructokinase consumes ATP and generates ADP. This reaction is activated by ADP thus establishing a substrate-depletion oscillator. ADP is removed linearly from the system. For simplicity, we consider the representation of the model as proposed in [11]. The model contains 2 variables, 3 rate coefficients, no nl-parameter and 1 cooperativity parameter.

$$\frac{\mathrm{d}S_1}{\mathrm{d}t} = k_1 - k_2 \cdot S_1 \cdot S_2^{n_1}$$
$$\frac{\mathrm{d}S_2}{\mathrm{d}t} = k_2 \cdot S_1 \cdot S_2^{n_1} - k_3 \cdot S_2$$

In the table below, the notation we used is given together with the notation used in the original publication [11]. Parameter values and initial conditions are one example for which the system shows sustained oscillations.

S_1	X	1	k_1	ν_1	3	k_3	k_3	4
S_2	Y	1	k_2	k_2	7	n_1	γ	2

Glycolysis model with Michaelis-Menten kinetics

In the second and third reaction of the glycolysis model from above, the linear mass action kinetics are replaced by Michaelis-Menten kinetics. The model contains 2 variables, 3 rate coefficients, 2 nl-parameters and 1 cooperativity parameter.

$$\frac{\mathrm{d}S_1}{\mathrm{d}t} = k_1 - k_2 \cdot \frac{S_1}{S_1 + kn_1} \cdot S_2^{n_1}$$
$$\frac{\mathrm{d}S_2}{\mathrm{d}t} = k_2 \cdot \frac{S_1}{S_1 + kn_1} \cdot S_2^{n_1} - k_3 \cdot \frac{S_2}{S_2 + kn_2}$$

Cell cycle model

The cell cycle model proposed in [12] captures the relationship between cdc2 and cyclin which form together the maturation promoting factor (MPF) in the cell. In detail,

cyclin forms complexes with phosphorylated cdc2, in which cyclin can be reversibly phosphorylated and cdc2 becomes dephosphorylated. The complex with phosphorylated cyclin and dephosphorylated cdc2 is the active form of MPF, which enhances its own formation. After disassemblance of the complex, phosphorylated cyclin is degraded, unphosphorylated cdc2 is released and can be reversibly phosphorylated. The model contains 6 variables, 10 rate coefficients, 1 nl-parameter and 1 cooperativity parameter.

$$\frac{dS_1}{dt} = k_6 \cdot S_4 - k_8 \cdot kn_1 \cdot S_1 + k_9 \cdot S_2$$
$$\frac{dS_2}{dt} = -k_3 \cdot S_2 \cdot S_5 + k_8 \cdot kn_1 \cdot S_1 - k_9 \cdot S_2$$
$$\frac{dS_3}{dt} = k_3 \cdot S_2 \cdot S_5 - k_{10} \cdot S_3 - k_4 \cdot S_3 \cdot \frac{S_4^{n_1}}{(S_1 + S_2 + S_3 + S_4)^{n_1}} + k_5 \cdot kn_1 \cdot S_4$$
$$\frac{dS_4}{dt} = k_{10} \cdot S_3 + k_4 \cdot S_3 \cdot \frac{S_4^{n_1}}{(S_1 + S_2 + S_3 + S_4)^{n_1}} - k_5 \cdot kn_1 \cdot S_4 - k_6 \cdot S_4$$
$$\frac{dS_5}{dt} = k_1 - k_2 \cdot S_5 - k_3 \cdot S_2 \cdot S_5$$
$$\frac{dS_6}{dt} = k_6 \cdot S_4 - k_7 \cdot S_6$$

In the table below, the notation we used is given together with the notation used in the original publication [12], and we provide the initial conditions and reference parameter set published together with the model.

S_1	$C2$	0	k_1	$k_1 \cdot [aa]$	$0.015\ min^{-1}$	k_7	k_7	$0.6\ min^{-1}$
S_2	CP	0.25	k_2	k_2	0	k_8	k_8	$10^6\ min^{-1}$
S_3	pM	0	k_3	k_3	$200\ min^{-1}$	k_9	k_9	$1000\ min^{-1}$
S_4	M	0.75	k_4	k_4	$180\ min^{-1}$	k_{10}	k_4'	$0.018\ min^{-1}$
S_5	Y	0	k_5	k_5	0	kn_1	P	1
S_6	Yp	0	k_6	k_6	$1\ min^{-1}$	n_1	n	2

Cell cycle model with Michaelis-Menten kinetics

All reaction rates with mass action kinetics are replaced by Michaelis-Menten kinetics. The bilinear complex formation (depending on CP and Y or S_2 and S_5) is replaced by two Michaelis-Menten terms. The model contains 6 variables, 10 rate coefficients, 11 nl-parameters and 1 cooperativity parameter.

$$
\begin{aligned}
\frac{\mathrm{d}S_1}{\mathrm{d}t} &= k_6 \cdot \frac{S_4}{kn_7 + S_4} - k_8 \cdot kn_1 \cdot \frac{S_1}{kn_9 + S_1} + k_9 \cdot \frac{S_2}{kn_{10} + S_2} \\
\frac{\mathrm{d}S_2}{\mathrm{d}t} &= -k_3 \cdot \frac{S_2}{kn_3 + S_2} \cdot \frac{S_5}{kn_4 + S_5} + k_8 \cdot kn_1 \cdot \frac{S_1}{kn_9 + S_1} - k_9 \cdot \frac{S_2}{kn_{10} + S_2} \\
\frac{\mathrm{d}S_3}{\mathrm{d}t} &= k_3 \cdot \frac{S_2}{kn_3 + S_2} \cdot \frac{S_5}{kn_4 + S_5} - k_{10} \cdot \frac{S_3}{kn_{11} + S_3} \\
&\quad - k_4 \cdot \frac{S_3}{kn_5 + S_3} \cdot \frac{S_4^{n_1}}{(S_1 + S_2 + S_3 + S_4)^{n_1}} + k_5 \cdot kn_1 \cdot \frac{S_4}{kn_6 + S_4} \\
\frac{\mathrm{d}S_4}{\mathrm{d}t} &= k_{10} \cdot \frac{S_3}{kn_{11} + S_3} + k_4 \cdot \frac{S_3}{kn_5 + S_3} \cdot \frac{S_4^{n_1}}{(S_1 + S_2 + S_3 + S_4)^{n_1}} \\
&\quad - k_5 \cdot kn_1 \cdot \frac{S_4}{kn_6 + S_4} - k_6 \cdot \frac{S_4}{kn_7 + S_4} \\
\frac{\mathrm{d}S_5}{\mathrm{d}t} &= k_1 - k_2 \cdot \frac{S_5}{kn_2 + S_5} - k_3 \cdot \frac{S_2}{kn_3 + S_2} \cdot \frac{S_5}{kn_4 + S_5} \\
\frac{\mathrm{d}S_6}{\mathrm{d}t} &= k_6 \cdot \frac{S_4}{kn_7 + S_4} - k_7 \cdot \frac{S_6}{kn_8 + S_6}
\end{aligned}
$$

FitzHugh-Nagumo model of neural dynamics

The FitzHugh-Nagumo model of neural dynamics is based on theoretical considerations of an oscillator which shows similar temporal characteristics as action potentials, i.e. one slow and one fast variable which generate excitable behavior with refractory phases [13]. The first variable is voltage-like. Except for k_4, which is considered to be the stimulating current, the terms defining the differential equation system do not have a direct biological counterpart. In order to have positive parameter values, we changed the sign of k_4. The model contains 2 variables and 8 parameters.

$$\frac{\mathrm{d}S_1}{\mathrm{d}t} = k_1 \cdot S_1 - k_2 \cdot S_1^{n_1} + k_3 \cdot S_2 - k_4$$
$$\frac{\mathrm{d}S_2}{\mathrm{d}t} = -k_5 \cdot S_1 - k_6 \cdot S_2 + k_7$$

In the table below, the notation used here is given together with the notation used in the original publication [13], and we provide the initial conditions and reference parameter set published together with the model.

S_1	x	-1	k_2	$c/3$	1	k_5	$1/c$	0.3333
S_2	y	0.5	k_3	k_2	0	k_6	b/c	0.2667
k_1	c	3	k_4	$c \cdot z$	1.2	k_7	$a \cdot c$	0.2333
n_1	n	3						

λ-ω oscillator

λ-ω oscillators, also referred to as A-B-oscillators or λ-ω systems, are a class of mathematical models of two variables generating Hopf bifurcations and thus limit cycle oscillations. Their solution can be obtained analytically by transformation into polar coordinates. The λ-ω oscillators are comprised of two functions, $\lambda : [0, \infty) \to \mathbb{R}$ and $\omega : [0, \infty) \to \mathbb{R}$, which are connected in an ordinary differential equation system as given below.

$$\frac{\mathrm{d}x}{\mathrm{d}t} = \lambda(\sqrt{x^2 + y^2}) \cdot x - \omega(\sqrt{x^2 + y^2}) \cdot y$$
$$\frac{\mathrm{d}y}{\mathrm{d}t} = \omega(\sqrt{x^2 + y^2}) \cdot x + \lambda(\sqrt{x^2 + y^2}) \cdot y$$

Thereby, $\sqrt{x^2 + y^2}$ is the radius of the 2-dimensional vector (x, y) in polar coordinates, and the analytical solution of such a system is given by

$$x(t) = r_0 \cdot \cos(w_0 \cdot t)$$
$$y(t) = r_0 \cdot \sin(w_0 \cdot t)$$

with $\lambda(r_0) = 0$, $\omega(r_0) = w_0$. Consequently, the amplitude is the same for x and y and is given by $r_0 = \lambda^{-1}(0)$, the period is given by $2\pi/w_0$. Note that the limit cycle is stable only for $\lambda'(r_0) < 0$.

We used the implementation of a λ-ω oscillator which has frequently been employed for various biological oscillations [14]. It employs the functions $\lambda(r) = k_1 \cdot (k_2 - r) \cdot (r - k_3)$ and $\omega(r) = k_4 - k_5 \cdot r$. Applying this, the model reads as given below. It contains 2 variables and 5 parameters.

$$\frac{\mathrm{d}S_1}{\mathrm{d}t} = k_1 \cdot (k_2 - \sqrt{S_1^2 + S_2^2}) \cdot (\sqrt{S_1^2 + S_2^2} - k_3) \cdot S_1 - (k_4 - k_5 \cdot \sqrt{S_1^2 + S_2^2}) \cdot S_2$$

$$\frac{\mathrm{d}S_2}{\mathrm{d}t} = (k_4 - k_5 \cdot \sqrt{S_1^2 + S_2^2}) \cdot S_1 + k_1 \cdot (k_2 - \sqrt{S_1^2 + S_2^2}) \cdot (\sqrt{S_1^2 + S_2^2} - k_3) \cdot S_2$$

In the table below, we provide the initial conditions and parameter values used in the original publication [14].

S_1	1	k_2	1	k_5	0.1
S_2	1	k_3	0.5		
k_1	5	k_4	1.1		

References

[1] Becker-Weimann S, Wolf J, Herzel H, Kramer A (2004) Modeling feedback loops of the mammalian circadian oscillator. Biophys J 87: 3023-34.

[2] Goldbeter A (1995) A model for circadian oscillations in the Drosophila period protein (PER). Proc Biol Sci 261: 319-24.

[3] Locke JCW, Southern MM, Kozma-Bognar L, Hibberd V, Brown PE, et al. (2005) Extension of a genetic network model by iterative experimentation and mathematical analysis. Mol Sys Biol 1.

[4] Goldbeter A, Dupont G, Berridge MJ (1990) Minimal model for signal-induced Ca2+ oscillations and for their frequency encoding through protein phosphorylation. Proc Natl Acad Sci U S A 87: 1461-5.

[5] De Young GW, Keizer J (1992) A single-pool inositol 1,4,5-trisphosphate-receptor-based model for agonist-stimulated oscillations in Ca2+ concentration. Proc Natl Acad Sci U S A 89: 9895-9.

[6] Sneyd J, Tsaneva-Atanasova K, Yule DI, Thompson JL, Shuttleworth TJ (2004) Control of calcium oscillations by membrane fluxes. Proc Natl Acad Sci U S A 101: 1392-6.

[7] Wolf J, Becker-Weimann S, Heinrich R (2005) Analysing the robustness of cellular rhythms. Syst Biol (Stevenage) 2: 35-41.

[8] Elowitz MB, Leibler S (2000) A synthetic oscillatory network of transcriptional regulators. Nature 403: 335-8.

[9] Kholodenko BN (2000) Negative feedback and ultrasensitivity can bring about oscillations in the mitogen-activated protein kinase cascades. Eur J Biochem 267: 1583-8.

[10] Sel'kov EE (1968) Self-oscillations in glycolysis. 1. A simple kinetic model. Eur J Biochem 4: 79-86.

[11] Wolf J, Heinrich R (1997) Dynamics of two-component biochemical systems in interacting cells; synchronization and desynchronization of oscillations and multiple steady states. Biosystems 43: 1-24.

[12] Tyson JJ (1991) Modeling the cell division cycle: cdc2 and cyclin interactions. Proc Natl Acad Sci U S A 88: 7328-32.

[13] FitzHugh R (1961) Impulses and physiological states in theoretical models of nerve membrane. Biophys J 1: 445-66.

[14] Winfree AT (1980) The Geometry of Biological Time, volume 8 of *Biomathematics*. New York: Springer-Verlag.
